# Ligand field design enables quantum manipulation of spins in Ni^2+^ complexes†

**DOI:** 10.1039/d3sc04919a

**Published:** 2023-12-08

**Authors:** Michael K. Wojnar, Krishnendu Kundu, Arailym Kairalapova, Xiaoling Wang, Andrew Ozarowski, Timothy C. Berkelbach, Stephen Hill, Danna E. Freedman

**Affiliations:** a Department of Chemistry, Massachusetts Institute of Technology Cambridge Massachusetts 02139 USA danna@mit.edu; b National High Magnetic Field Laboratory Tallahassee Florida 32310 USA; c Department of Physics, Florida State University Florida 32306 USA; d Department of Chemistry, Columbia University New York New York 10027 USA

## Abstract

Creating the next generation of quantum systems requires control and tunability, which are key features of molecules. To design these systems, one must consider the ground-state and excited-state manifolds. One class of systems with promise for quantum sensing applications, which require water solubility, are d^8^ Ni^2+^ ions in octahedral symmetry. Yet, most Ni^2+^ complexes feature large zero-field splitting, precluding manipulation by commercial microwave sources due to the relatively large spin–orbit coupling constant of Ni^2+^ (630 cm^−1^). Since low lying excited states also influence axial zero-field splitting, *D*, a combination of strong field ligands and rigidly held octahedral symmetry can ameliorate these challenges. Towards these ends, we performed a theoretical and computational analysis of the electronic and magnetic structure of a molecular qubit, focusing on the impact of ligand field strength on *D*. Based on those results, we synthesized 1, [Ni(ttcn)_2_](BF_4_)_2_ (ttcn = 1,4,7-trithiacyclononane), which we computationally predict will have a small *D* (*D*_calc_ = +1.15 cm^−1^). High-field high-frequency electron paramagnetic resonance (EPR) data yield spin Hamiltonian parameters: *g*_*x*_ = 2.1018(15), *g*_*x*_ = 2.1079(15), *g*_*x*_ = 2.0964(14), *D* = +0.555(8) cm^−1^ and *E* = +0.072(5) cm^−1^, which confirm the expected weak zero-field splitting. Dilution of 1 in the diamagnetic Zn analogue, [Ni_0.01_Zn_0.99_(ttcn)_2_](BF_4_)_2_ (1′) led to a slight increase in *D* to ∼0.9 cm^−1^. The design criteria in minimizing *D* in 1*via* combined computational and experimental methods demonstrates a path forward for EPR and optical addressability of a general class of *S* = 1 spins.

## Abstract 2

Molecular qubits with the potential for optical read-out require careful ligand design to control zero-field splitting, *D*, for quantum manipulation. We find Ni^2+^ in a sulfur ligand field in a near-ideal octahedral environment enables quantum control.

## Graphical abstract

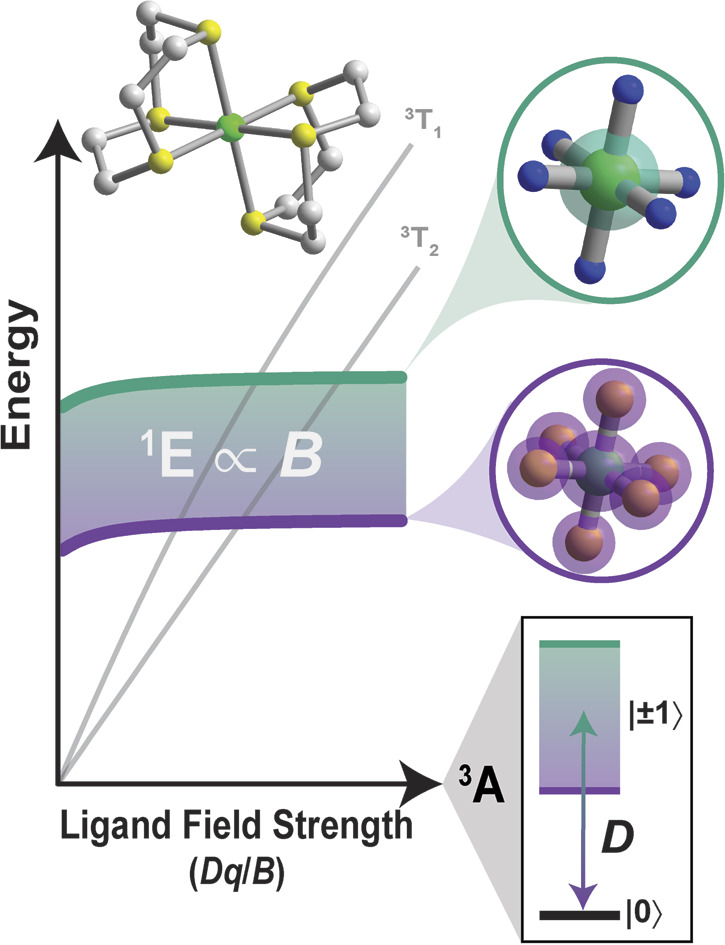

## Introduction

The emerging field of quantum information science (QIS) promises to revolutionize myriad areas, including computation, metrology, networking, and sensing.^1,2^ The fundamental unit of these advancing technologies revolves around the quantum bit, or qubit. Among the well-established qubit candidates are electron spin bearing defects, such as the anionic nitrogen-vacancy pair defect in diamond^3,4^ and the neutral divacancy in silicon carbide,^5–7^ where the state of the spin may be prepared and read out with optical light, even to the single-spin level. This spin–optical interface has resulted in rapid progress in the field of nanoscale and quantum sensing, in which these defects detect minute changes in temperature,^8,9^ pressure,^10^ magnetic fields,^11–13^ and electric fields.^14–16^

Translating the electronic structure of these optically-addressable solid-state spin defects (an *S* = 1 ground state with radiative decay from the excited state) to molecules allows for the combination of the sensitivity of spin with the tunability of molecules. Recent studies of a molecular bottom-up approach to QIS demonstrated molecular control over coherence properties, spectral resolution, interspin distance, as well as optical addressability in molecular spins.^17–22^ Molecular electronic spin-based qubits may also improve on previously mentioned sensing applications, as the molecular sensor can be placed in direct proximity to the target analyte with Ångstrom-scale precision. A spin–optical interface also primes these tunable “molecular color centers” for potential integration into quantum networks.^23^ We are interested in implementing an atom-by-atom synthetic approach and engineering the energy levels (ground-state magnetic spin sublevels, excited states tuned by ligand fields) of electronic spin-based transition metal complexes for the manipulation of the molecular spin.

Octahedral (*O*_h_) *S* = 1 Ni^2+^ complexes represent an enticing class of molecular quantum sensors due to the potential biocompatibility, as indicated by their chemical stability in the presence of air and water of this general class of molecules and the redox stability of the metal ion. While they feature the required electronic structure for a spin–optical interface, transition metal complexes exhibit large values (>90 GHz) of zero-field splitting (ZFS), due to the large spin–orbit coupling (SOC) constant (630 cm^−1^ for Ni^2+^), hindering the measurement of ground-state spin properties of these systems with commercial microwave sources (9 GHz to 90 GHz). Consequently, there is a dearth of information on the dynamic spin properties of Ni^2+^ spins, without which we lack fundamental understanding of the coherence behavior of the electronic spin.^24^ Better understanding the characteristics of this class of molecules, in addition to the added potential for optical addressability, may enable the use of these spin-based qubits for biological quantum sensing.

The criteria for a molecular quantum sensor include having discrete, well-defined quantum states that are addressable for manipulation (microwave, radiofrequency excitations). For *S* = 1 transition-metal-based electronic spin systems, EPR addressability is achieved by minimizing axial ZFS, *D*. The challenge for late transition metals relates to their large SOC constants, which requires careful tuning of the ligand field in order to minimize the ZFS. In the orbitally non-degenerate case, this can be achieved *via* incorporation of strong field ligands, as *D* is inversely proportional to the energetic separation of excited states from the ground state.^25^ Previously, the only examples of commercial frequency (9–35 GHz) continuous-wave (cw) EPR investigations on *O*_h_ Ni^2+^ spins have focused on [Ni(pyrazole)_6_]^2+^, [Ni(H_2_O)_6_]^2+^ and [Ni(NH_3_)_6_]^2+^,^26–33^ which are exceptionally host- and counterion-dependent, due to the weak field monodentate ligands and the flexible primary coordination sphere. As a result, for a nickel coordination complex with the same coordinating ligands, *D* can vary by an order of magnitude as a function of counterion.^34^ Moreover, the weak ligand field also does not exhibit the required electronic structure for a spin–optical interface.

In order to tune the axial ZFS, *D*, into a desirable microwave energy regime, we can use coordination chemistry to control both the excited state contributions as well as the effective SOC constant, *ε*_eff_.^35,36^ A strong interaction between metal and ligands is necessary so that contributions to *D* are minimized.^37^ Strong field ligands ensure that the triplet excited states are high in energy, reducing the energetic separation of the ground-state *M*_S_ = 0 and *M*_S_ = ±1 spin triplet sublevels. We recently demonstrated this in Cr^4+^, V^3+^, and Ni^2+^ transition metal complexes with aryl, amide, and polypyridine ligands, respectively.^21,38–40^ We can chemically tune the effective SOC constant through the relativistic nephelauxetic effect or interelectronic repulsion/metal–ligand covalency. The latter effect is typically quantified by the Racah parameter, *B*,^41–43^ which decreases with decreasing electronic repulsion. Therefore, in systems where polarizable ligands minimize electron repulsion by electron delocalization (*i.e.*, increased covalency), *B* decreases.^44,45^ Ni^2+^ ions are perfectly geared for ground- and excited-state engineering and characterization of the electronic state manifold due to the abundance of literature on *O*_h_ Ni^2+^ ligand field strength.^46–50^ Moreover, out of the first-row transition metals that can host a ground-state *S* = 1 spin, Ni^2+^ has the largest SOC constant, and thus should exhibit the largest changes in *D* as a function of ligand environment.^43^

## Results and discussion

**Fig. 1:**
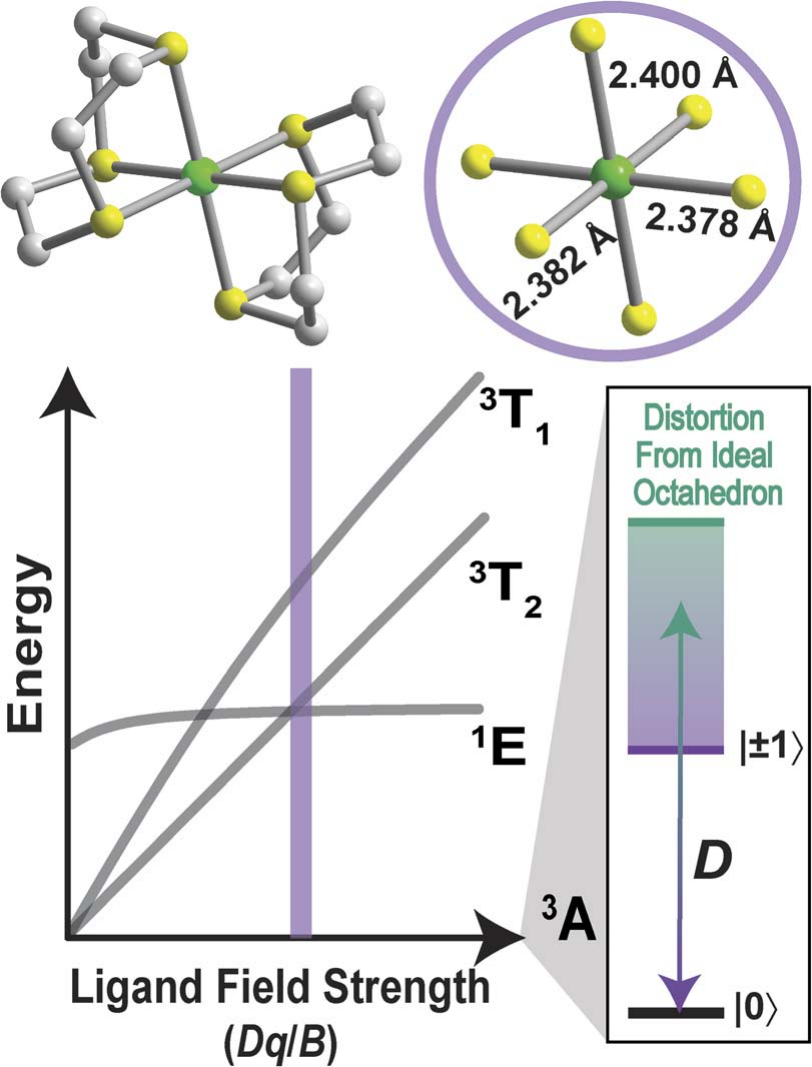
Molecular structure, as found in the crystal structure, of [Ni(ttcn)_2_]^2+^ (1) with Ni–S bond lengths highlighting the small distortion from an ideal octahedron and a simplified Tanabe–Sugano diagram of 1, illustrating the energetic ordering of excited states. The scale designates the distortion from an ideal octahedron and its impact on axial zero-field splitting, *D*. Green, yellow, blue, and gray spheres represent Ni, S, N, and C atoms, respectively.

To probe the relativistic nephelauxetic effect in *O*_h_ Ni^2+^ and its effect on the ground-state spin, we elected for a sulfur-rich coordination environment around the ion (Fig. 1). We selected a Ni^2+^ complex, [Ni(ttcn)_2_](BF_4_)_2_ (compound 1),^51,52^ with the thioether-based ttcn ligand (ttcn = 1,4,7-trithiacyclononane) for these investigations because it meets our criteria for an EPR-addressable electronic spin-based qubit by combatting the two main contributions to *D* (SOC and low-lying excited states): (1) the soft sulfur donors give rise to increased covalency, thus reducing metal-based SOC; (2) the ligand field strength of the thioether donors is strong (similar to pyridine donors), pushing the triplet excited states to higher energy, thus minimizing excited state contributions to *D*; (3) the ethylene spacers in the ttcn ligand allow for minimal distortion of the octahedral coordination environment around Ni^2+^ (Fig. 1).^53^ The strong metal–ligand interaction in 1 is evident from the crystal structure data, where the Ni–S bond lengths range from 2.378–2.400 Å, the shortest Ni–S bond lengths in the CCDC database for a NiS_6_ coordination environment. The primary coordination sphere around the metal ion is close to a perfect octahedron: the average deviations from 90° for the 12 *cis* angles is *Σ*_mean_ = 1.48° (*Σ*_sum_ = 17.76°).

**Fig. 2:**
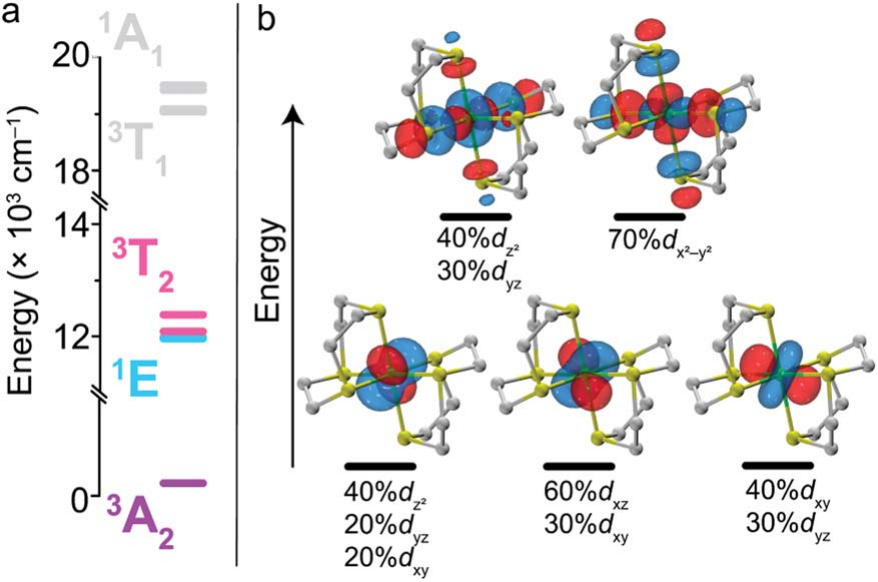
(a) CASSCF-NEVPT2 (12,12) calculation of the energy level diagram of 1, with colors chosen for visual contrast. (b) Five partially-occupied orbitals from CASSCF (12,12) calculation with isosurface value of 0.03 a.u. Ni-atom d-orbital contributions greater than 20% are listed (for simplicity, rounded to the nearest 10%).

We began by employing computational methods to screen for and predict the ground- and excited-state properties of [Ni(ttcn)_2_]^2+^, to see if 1 meets our requirements for a qubit (small ZFS and lowest-lying singlet state for potential spin–flip luminescence). Due to the multiconfigurational nature of the electronic states of 1, we calculate ground- and excited-state energies using the complete active space self-consistent field (CASSCF) method,^54^ including corrections with second-order *n*-electron valence state perturbation theory (NEVPT2) method.^55^ We have performed calculations using both the minimal (8,5) active space for 1, which has eight electrons in the five 3d-orbitals of Ni^2+^, and a larger (12,12) active space, which adds two doubly-occupied orbitals corresponding to a π bond (with Ni d-orbital character) and five unoccupied 4d-orbitals of Ni^2+^ (Table S7†). All calculations are performed with the ORCA program,^56^ and computational details are available in the ESI.† The Ni^2+^ 3d-orbitals from CASSCF (12,12) are illustrated in Fig. 2. Their energy distribution shows a nearly ideal *O*_h_ ligand field splitting: three t_2g_ orbitals (with >95% metal character) lie 21 000 cm^−1^ below two e_g_ orbitals, although the singly-occupied e_g_ orbitals have nonnegligible ligand character (about 15%) due to mixing with sulfur p-orbitals.

As expected, our calculations find that 1 is a ground-state *S* = 1 triplet, ^3^A_2g_. The calculated triplet excited states for each T state in *O*_h_ symmetry (^3^T_2g_, ^3^T_1g_) fall within 450 cm^−1^ of each other, which again reflects the nearly ideal octahedral environment. This symmetry causes the calculated transition dipole moments among the d-orbitals to be minuscule (10^−4^*D*), resulting in zero oscillator strength and absorption intensity. The lowest lying triplet manifold, ^3^T_2g_, has a mean energy of 12 158 cm^−1^ (822.5 nm) above the ground state. The next triplet manifold, ^3^T_1g_, has a mean energy of 19 208 cm^−1^ (520.6 nm). These high-energy states illustrate the ligand field strength of ttcn resulting in the ligand field splitting, *Dq*, of about 1216 cm^−1^ corresponding to 1/10 energy of the ^3^A_2g_ → ^3^T_2g_ transition. These triplet excited-state energies are in good agreement with the experimental absorption spectra discussed below (Table S8†). Additionally, we calculate the energy of the lowest lying singlet state, ^1^E_g_, to be 11 950 cm^−1^ (836.8 nm). The ^1^E_g_ and ^3^T_2g_ states have similar energies, possibly explaining the broadening of ^3^T_2g_ state and the absence of a separate ^1^E_g_ peak in the experimental absorption spectrum (Fig. S32†). Using Tanabe and Sugano's analytical expressions for excited state energies, we estimate the Racah parameters as *B* = 897 cm^−1^, *C* = 2574 cm^−1^ and *C*/*B* is 2.87.^57^ Despite the triplet energies being very close to the experimental absorption spectra discussed below, the theoretical and experimental Racah parameters are quite different (Table 1). This highlights that Racah parameters are highly sensitive to the absorption energies.^58^ In the ESI,† we provide results for higher lying states (Table S8†) and CASSCF configurations of each excited triplet and singlet state in terms of the five d-orbitals from Fig. 2 (Tables S9 and S10†).

**Table 1:** Simulated spin Hamiltonian parameters, optical properties, and Racah parameters for 1

	*g*_iso_	*D*, cm^−1^	|*E*| (cm^−1^)	*Dq* (cm^−1^)	*B* (cm^−1^)	*C* (cm^−1^)	*C*/*B*
1	2.10	+0.555(8)	0.072(5)	1279	673	2190	3.29
1^calc^	2.16	+1.15	0.3	1216	897	2574	2.87

We calculate the excited-state contributions to ZFS, which provide a manner in which to rationalize and optimize the magnitudes of ZFS in *O*_h_ Ni^2+^ spins to screen 1 as a molecular spin with low ZFS for commercial microwave addressability. When the symmetry of a complex reduces from a perfect *O*_h_, the ground state triplet splits into three spin sublevels due to the coupling to the excited states. Since the excited state energies for 1 are relatively high for Ni^2+^ complexes, we expect to observe small *D* values. Within perturbation theory, the elements of the *D* tensor are1
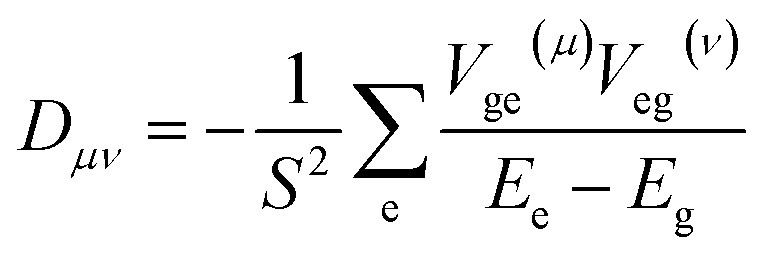
where *S* is the quantum spin, *E* is the energy of the ground (g) or excited (e) state, and *V*_ge_^(*μ*)^ are matrix elements of the spin–orbit coupling operator: 
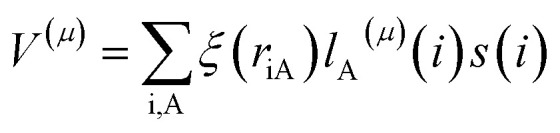
 for electron i and nucleus A separated by *r*_iA_, where *ξ*(*r*_iA_) is the radial operator, *l*_A_^(*μ*)^(*i*) is the *μ* component of the orbital angular momentum, and *s*(*i*) is the *z* component of the spin operator.^59,60^ The ZFS parameters are obtained from the eigenvalues of the *D* tensor.

**Fig. 3:**
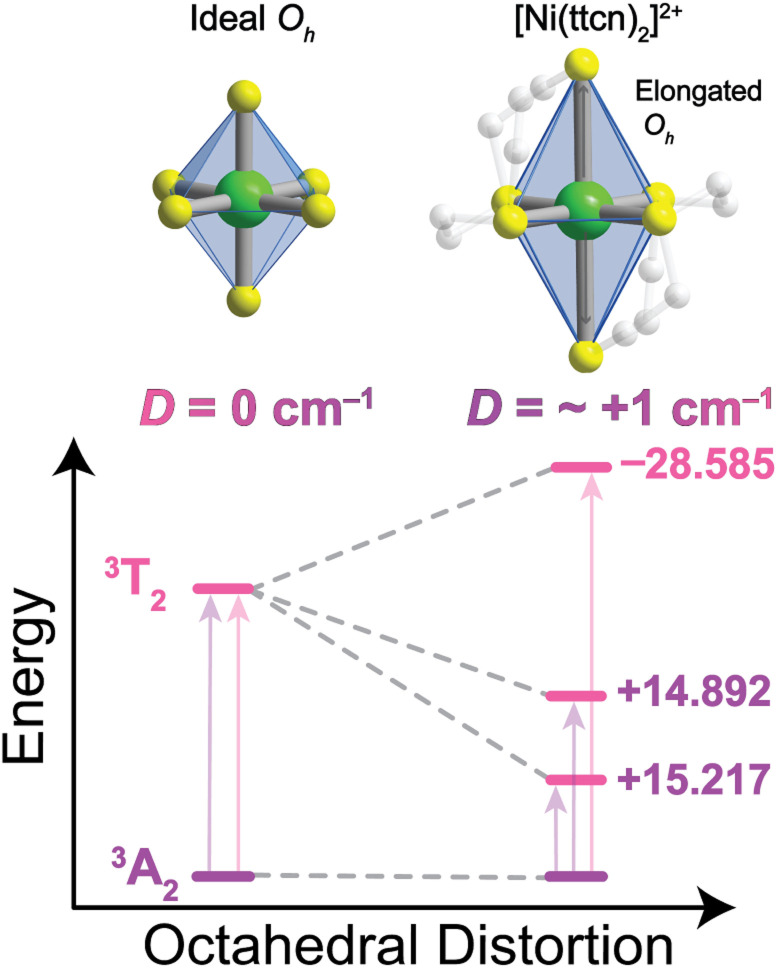
CASSCF-NEVPT2 (12,12) calculation of ground- and excited-state energies, demonstrating the octahedral distortion contributions to axial ZFS, *D*. This figure only considers values from the ^3^T_2g_ state, indicating the different positive (purple) and negative (pink) largest contributions to *D* that yield a *D*_calc_ = +1.52 cm^−1^. Most of the difference with the final value comes from mixing with the ^1^T_2g_ state, which is of opposite sign, with only very minor contributions from other states.

The calculated ZFS parameters are *D*_calc_ = +1.15 cm^−1^ and *E*/*D* = 0.26, which are comparable to our experimentally measured values (described below). As can be seen from (1), the excited states with higher energies have less significant contributions to the ZFS.^61^ We find that the contributions to *D*_calc_ predominantly come from the six lowest-lying orbital states with the necessary symmetry: three triplet ^3^T_2g_ and three singlet ^1^T_2g_; the positive sign indicates that *M*_S_ = 0 is lower in energy than *M*_S_ = ±1 (Table S8†). The electron configurations of these six orbital states are nearly identical (Tables S9 and S10†), but the triplet and singlet states contribute with opposite signs due to the nature of the coupling of the spin operators (see ESI† for further discussion).^62,63^ The largest contributions to *D*_calc_ come from the three triplet ^3^T_2g_ states (Fig. 3), with +15.22 cm^−1^, +14.89 cm^−1^, −28.59 cm^−1^ (*D*_sum_ = +1.52 cm^−1^), and the next largest contributions come from the three singlet ^1^T_2g_ states with −7.34 cm^−1^, −7.28 cm^−1^, and +14.23 cm^−1^ (*D*_sum_ = −0.39 cm^−1^). The transition energy to the ^1^T_2g_ state is more than twice the transition energy to the ^3^T_2g_ state, causing the ZFS contributions to be half as large. Taken together, these six roots lead to a computed axial ZFS value of +1.13 cm^−1^, 98% of the total value. The higher lying ^3^T_1g_ excited states have minimal contributions to *D* (<0.01 cm^−1^, Table S8†) which was previously observed for other Ni^2+^ complexes.^62^ The energetically lower ^1^E_g_ and ^1^A_1g_ singlet states contribute less than 0.1 cm^−1^ because they have the wrong symmetry. The results demonstrate that 1 has a computationally-predicted low ZFS.

To computationally study the impact of the ligand, we performed calculations with the minimal (8,5) active space for both the near-ideal *O*_h_ “soft” sulfur-rich [Ni(ttcn)_2_]^2+^ and the distorted “hard” nitrogen-rich [Ni(tacn)_2_]^2+^ complexes (tacn = 1,4,7-triazacyclononane). We confirm that for 1, our results are qualitatively unchanged with this smaller active space. Specifically, we find that the value of *D*_calc_ (1.07 cm^−1^) and the six main contributions from triplet ^3^T_2g_ and singlet ^1^T_2g_ states are similar for (8,5) and (12,12) active spaces (Tables S11 and S12†). The calculation for the [Ni(tacn)_2_]^2+^ system predicts a ZFS of 4.27 cm^−1^, compared to the experimental value of 2.95 cm^−1^.^62^ The same six states contribute to the *N*-substituted compound, although the triplet contributions are different. Three sublevels comprising the ^3^T_2g_ state effectively cancel one another in [Ni(ttcn)_2_]^2+^, while they do not cancel in [Ni(tacn)_2_]^2+^, leading to the larger ZFS value (Table S12†). As [Ni(tacn)_2_]^2+^ benefits from a slightly larger ligand field strength compared to [Ni(ttcn)_2_]^2+^, this study provided an *ab initio* demonstration that *D* decreases (from 4.27 cm^−1^ to 1.07 cm^−1^) when we employ a softer ligand in a more rigorously octahedral environment. While stronger ligand fields generally minimize *D*, we highlight minimizing octahedral distortion will push *D* towards rigorously zero.

**Fig. 4:**
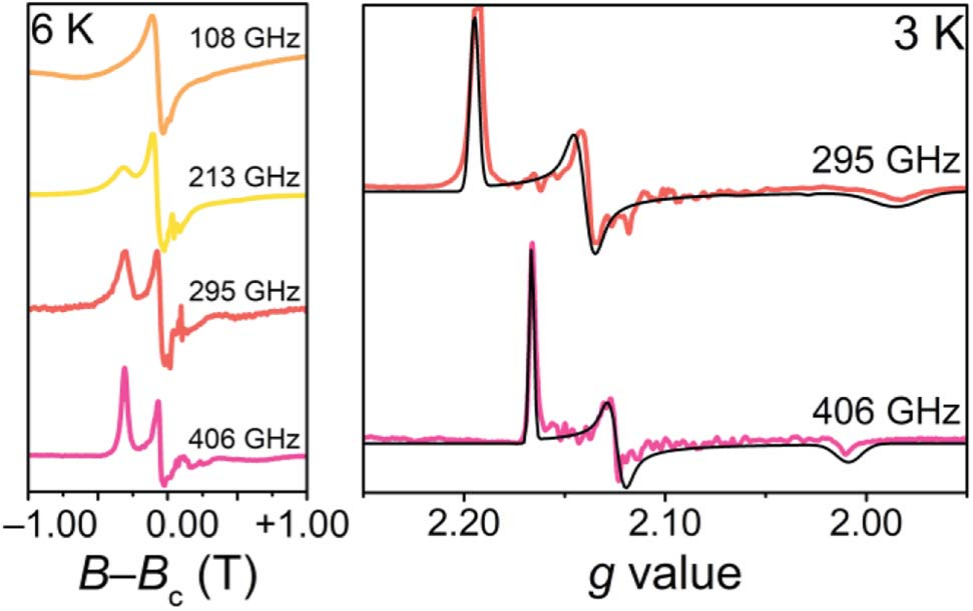
High-field high-frequency EPR spectra for 1 at 108 GHz, 213 GHz, 295 GHz, and 406 GHz at 6 K (left), plotted as a function of the deviation, *B*–*B*_c_, from the center field of each spectrum, *B*_c_. High-field high-frequency EPR spectra for 1 at 295 GHz and 406 GHz and 3 K (right), plotted as a function of *g* value. The best simulations are shown in black spectra.

To confirm the ground-state magnetic properties and EPR addressability of 1 that we screened for computationally, we utilized high-field high-frequency cw-EPR spectroscopy (Fig. 4). The observed spectra were modelled with EasySpin and the spin Hamiltonian, 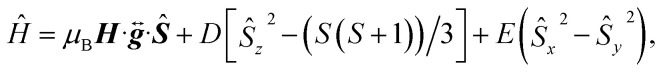 which provides the energies of the *M*_S_ levels for the spin **S** as a function of *D* and *E* as well as the applied dc magnetic field (**H**). In this Hamiltonian, *Ŝ*_*i*_ (*i* = *x*, *y*, and *z*) are the spin component operators, *g*_*i*_ (*i* = *x*, *y*, and *z*), the components of the diagonal ***g***-tensor, and *μ*_B_, the Bohr magneton.^64^ EPR spectroscopy corroborates the *S* = 1 ground state, and enabled quantification of the axial and rhombic ZFS parameters, *D* and *E*. While the resonances broaden with increasing temperature, indicative of competing long-range spin interactions and thermal fluctuations,^65^ resolved magnetic resonances could be observed and accurately simulated at 3 K. The best simulations for 1 are *g*_*x*_ = 2.1018(15), *g*_*y*_ = 2.1079(15), *g*_*z*_ = 2.0964(14), *D* = +0.555(8) cm^−1^, *E* = +0.072(5) cm^−1^ (*E*/*D* = 0.13) (Fig. 4). We simulate the spectra best with a positive value for *D*. The *g*_iso_ (2.1018) and ZFS (*D* = +0.555 cm^−1^, 18 GHz) parameters in 1 represent some of the lowest values for *O*_h_ Ni^2+^, with the other low ZFS nickel complexes incorporating monodentate weak-field ligands, such as aqua or pyrazole ligands, in rigorously symmetric octahedral coordination environments.^26,66–68^ The *g*_iso_ value in 1 is closer to *g*_e_ than in other Ni complexes that exhibit *g* values from ∼2.11 to 2.30.^69^ This indicates electron delocalization onto the ligands, resulting in the reduction of the effective spin–orbit coupling constant. A similar effect of reduced *g* values was observed for the [Co(ttcn)_2_]^2+^ (*g*_iso_ = 2.067)^53^ and [Cu(ttcn)_2_]^2+^ (*g*_iso_ = 2.06)^70,71^ complexes. Moreover, the large contribution of ligand character into the frontier molecular orbitals, *i.e.*, the unpaired electrons are more delocalized, is also apparent in the EPR spectrum and the associated spin Hamiltonian parameters of the isolated [Ni(ttcn)_2_]^3+^ complex (*g*_iso_ = 2.065).^72^ We attribute the *g* values closer to *g*_e_ and the low ZFS in 1 to the previously mentioned interconnected variables: reduction of the metal-based SOC constant of nickel *via* the relativistic nephelauxetic effect^59^ of the covalent Ni–S interactions. The strong ligand field and near *O*_h_ geometry around nickel pushes the excited states to higher energy. We can also compare the low ZFS in 1 with the magnetic parameters in [Ni(tacn)_2_]^2+^, in which *D* = +2.95 cm^−1^ (88.4 GHz).^62^ The magnitudes of the ZFS parameters in 1 confirm the EPR addressability of the complex with commercial microwave sources.

To evaluate the coherence properties of 1 and determine the field and frequency dependence of the spin coherence, we employed multifrequency pulse EPR at both W-band (94 GHz) and Q-band (34 GHz). All pulse EPR experiments were performed on diamagnetic dilutions of [Ni(ttcn)_2_](BF)_2_ into the zinc analogue, [Ni_0.01_Zn_0.99_(ttcn)_2_](BF_4_)_2_ (1′), to mitigate the detrimental coupling between Ni^2+^ centers, as was observed by SQUID magnetometry (Fig. S5†). We first measured the echo-detected field-swept (EDFS) spectra for 1′ at both W- and Q-band by applying a two-pulse Hahn-echo detection sequence (π/2–*τ*–π–*τ*–echo) at a fixed *τ* value while sweeping magnetic field. At W-band, the resultant EDFS spectrum of 1 exhibits an intense and broad transition at 2.50 T and a less intense yet sharp transition at 1.33 T (Fig. S18–S21†). At Q-band, the transitions shift to lower fields, with an intense and broad transition at 0.69 T, with a less intense and broad transition at 0.25 T (Fig. S22 and S23†). Variable-temperature (12 K, 20 K, 30 K) EDFS spectra at Q-band also do not exhibit magnetic field shifts in transitions, indicative of the rigid ttcn ligand, as well as the crystalline matrix, minimizing changes in ZFS as a function of temperature. This is in stark contrast to EPR investigations on other *O*_h_ Ni^2+^ complexes, like [Ni(H_2_O)_6_]^2+^, where changes in temperature and pressure caused dramatic increases in ZFS.^66,73^ The low field feature in both spectra corresponds to the spin-forbidden, Δ*M*_S_ = 2 transitions, while the broader features originate from spin-allowed, Δ*M*_S_ = 1.

While the EDFS spectra for 1′ are consistent with predicted spectra based on the magnetic parameters obtained from cw-EPR measurements, there are discrepancies in the ZFS values between 1 and 1′. We attribute these differences in magnetic properties to the crystallization and dilution of 1 in the zinc analogue, as well as cooling to cryogenic temperatures. 1 crystallizes in the monoclinic *P*2_1_/*c* space group at 200 K, as two crystallographically independent complexes that lie on an inversion center.^74^ The zinc complex, [Zn(ttcn)_2_](BF_4_)_2_·2(MeNO_2_) crystallizes in the orthorhombic *Pbca* space group at 295 K, with one independent complex that lies on an inversion center.^75^ Variation in lattice parameters and ZFS upon dilution has previously been demonstrated in Ni^2+^ quantum magnets as a consequence of anisotropic expansion of M–L bond distances;^76,77^ enhanced spin coherence in Cr^4+^ spins has also been shown upon dilution.^22^ Changes in crystal packing incurred through cooling and dilution in a different symmetry space group for [Zn(ttcn)_2_]^2+^ result in an increase in |*D*| from 0.60 cm^−1^ (18 GHz)in 1 to two distinct species in 1′ with |*D*| values of ∼0.9 cm^−1^ (27 GHz) and ∼1.15 cm^−1^ (34 GHz), increases of 50% and 110%, respectively, based on simulations of the EDFS spectra (Fig. S20 and S21†).^22^ The non-zero echo intensity at both 94 GHz and 34 GHz obtained for 1′ demonstrates that we can manipulate multiple transitions in the spin manifold coherently.

**Fig. 5:**
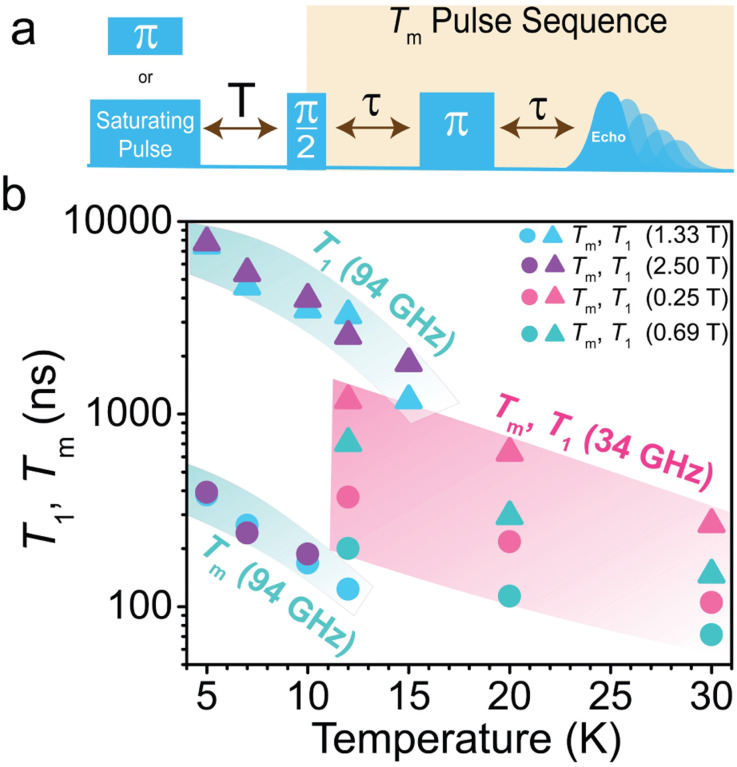
(a) Pulse sequence of pulse EPR experiments measuring *T*_1_ and *T*_m_. For *T*_1_, we implement a saturation recovery pulse sequence (long pulse–*T*–π/2–*τ*–π–*τ*–echo) at 94 GHz, and an inversion recovery pulse sequence (π–*T*–π/2–*τ*–π–*τ*–echo) at 34 GHz. For *T*_m_, we implement a standard Hahn-echo pulse sequence (π/2–*τ*–π–*τ*–echo) (b) Temperature dependence of *T*_1_ and *T*_m_ at 94 GHz and 34 GHz. Teal and pink highlights denote coherence times at 94 GHz and 34 GHz, respectively.

We proceeded to determine the phase memory time (*T*_m_), which encompasses all processes that contribute to decoherence or loss of phase memory, including the electron spin *T*_2_. We extract these parameters from fitting the decay of the intensity of the two-pulse Hahn-echo sequence (π/2–*τ*–π–*τ*–echo) with increasing interpulse delay time *τ* at both W- and Q-band. We fit the echo decays to mono-exponential functions (*I* = *I*_0_e^−2*τ*/*T*_m_^). For 1′, *T*_m_ values decay rapidly with increasing temperatures (we provide a full discussion of *T*_m_ in the ESI†). We next measured the spin–lattice relaxation time, *T*_1_, the time it takes for an ensemble of spins to relax to thermal equilibrium (Fig. 5). To measure *T*_1_, we employed two separate pulse sequences for W- and Q-band, due to instrumental limitations. For W-band, we implemented a saturation recovery pulse sequence (long pulse–*T*–π/2–*τ*–π–*τ*–echo), where a long microwave pulse saturates the spin transition. Following the initial pulse saturation, we applied two microwave pulses to detect the realignment of the spin with the dc field as a function of delay time, *T*, after the long pulse. For Q-band pulse experiments, we employed an inversion recovery pulse sequence (π–*T*–π/2–*τ*–π–*τ*–echo). We acknowledge that spin–lattice relaxation is dependent on the experimental parameters. Spectral diffusion is a convoluting factor in inversion recovery pulse sequences, in which magnetic interactions modify the resonant frequency of excited spins, thereby shortening *T*_1_. We fit both saturation and inversion recovery curves with a monoexponential function, *I* = −*I*_0_(e^−*T*/*T*_1_^ − 1). Spin–lattice relaxation is fast in comparison to other transition-metal-based *S* = 1 spins^21,38^ and drops precipitously with increasing temperature and decreasing field (Fig. 5, Table S2†). At 94 GHz and 5 K, *T*_1_ is 7.5–7.8 μs and drops to <2 μs at 12 K. At 34 GHz and 12 K, *T*_1_ is 0.71–1.2 μs and drops to <0.3 μs at 30 K. This is consistent with the direct and Raman processes limiting *T*_1_ at W- and Q-band, respectively which was previously seen in pulse EPR data on *S* = 1 Ni^2+^.^39^ This combination of direct and Raman-driven relaxation is also in agreement with *T*_1_ data on *O*_h_*S* = 1 Ni^2+^ data derived from frequency-dependent alternating current (ac) susceptibility experiments at high fields as observed here.^78^ While both the direct and Raman processes are inversely dependent on field, we observe an increase in *T*_1_ at higher magnetic fields in 1′. We attribute this observation to either spectral diffusion associated with the inversion recovery pulse sequence or to *M*_S_ spin sublevel mixing, which is more prominent at lower frequency and field. This was previously demonstrated for a high-spin Fe^3+^ system.^79^

The electronic absorption spectroscopy data corroborates the computationally-predicted ligand field strength in 1 and provides critical information on the excited state manifold. We observe two peaks in the UV-Vis spectrum of 1 in acetonitrile (Fig. S32†), which correspond to the spin-allowed ligand field transitions: (1) ^3^A_2g_ → ^3^T_2g_ at 782 nm (12 788 cm^−1^) (2) ^3^A_2g_ → ^3^T_1g_ at 527 nm (18 975 cm^−1^), consistent with *O*_h_ Ni^2+^. We did not observe ^1^E excited state absorption or photoluminescence. With the two observed transitions, we calculate the parameters for *Dq* and *B*.^57,80^ The ^3^A_2g_ → ^3^T_2g_ transition energy is equal to 10 *Dq* as it describes the promotion of an electron from the non-bonding t_2g_ set of orbitals to the anti-bonding e_g_ orbitals; we calculate *Dq* to be 1279 cm^−1^. This confirms a strong interaction between the metal d orbitals and the sulfur p orbitals (for comparison, *Dq* for [Ni(tacn)_2_]^2+^ is 1250 cm^−1^ and for [Ni(bipyridine)_3_]^2+^*Dq* is 1270 cm^−1^).^58,81,82^ We calculate the Racah *B* parameter (covalency parameter) to be 673 cm^−1^.^57,80,83,84^ This value is 66% of the free-ion value (1030 cm^−1^),^83^ and significantly smaller in comparison to other Ni^2+^ complexes, including [Ni(tacn)_2_]^2+^ (*B*_solution_ = 993 cm^−1^ and *B*_single crystal_ = 840 cm^−1^).^81,85–89^ The electronic absorption spectroscopy data demonstrates that the thioether ligand provides a strong ligand field around the nickel ion and leads to a reduction in the interelectronic repulsion parameter, *B*.

## Conclusions

The results provide a joint computational and experimental approach toward targeting *O*_h_ Ni^2+^ spins with low axial ZFS to allow for spin manipulation at W/Q-band coupled with the requisite electronic structure for a spin–optical interface. The soft and rigid coordination environment provided by the tridentate thioether ligand leads to a strong field environment around the ion, together with decreased SOC, which contributes to the small ZFS. Nearly ideal octahedral coordination environments have been realized with other Ni^2+^ complexes with harder nitrogen donors, with much larger values of axial ZFS (∼3 cm^−1^), highlighting the combination of geometry and SOC in minimizing *D*.^39^ Moreover, the rigidity of the ttcn ligand maintains a low ZFS across host matrix, previously not observed for other low ZFS Ni^2+^ qubits, such as [Ni(H_2_O)_6_]^2+^, [Ni(pyrazole)_6_]^2+^, and [Ni(NH_3_)_6_]^2+^. The biocompatibility of these metal ions allows for the potential of sensing under aqueous conditions or even integrating spin qubits into biological architectures, such as DNA, where the orientation and environment of the electronic spin qubit can be controlled.^90–92^1 also possesses the correct electronic structure for optical initialization and readout, but suffers from low extinction coefficients. Incorporating ligands that increase the extinction coefficients (extended planar polypyridine ligands) and ligand field strengths (N-heterocyclic carbenes) of this general class of molecules,^93^ while maintaining the symmetric primary coordination sphere and lowest-lying excited states, will be the focus of future work.

## Data availability

The datasets supporting this article have been uploaded as part of the ESI.†

## Author contributions

Conceptualization: M. K. W. and D. E. F.; investigation and formal analysis: M. K. W., K. K., A. K., X. W., and A. O.; resources: X. W., A. O., and S. H.; supervision: D. E. F., S. H., and T. C. B.; all authors contributed to writing the manuscript.

## Conflicts of interest

There are no conflicts to declare.

## Supplementary Material

SC-015-D3SC04919A-s001

## References

[cit1] Atzori M., Sessoli R. (2019). The Second Quantum Revolution: Role and Challenges of Molecular Chemistry. J. Am. Chem. Soc..

[cit2] Wasielewski M. R., Forbes M. D. E., Frank N. L., Kowalski K., Scholes G. D., Yuen-Zhou J., Baldo M. A., Freedman D. E., Goldsmith R. H., Goodson T., Kirk M. L., McCusker J. K., Ogilvie J. P., Shultz D. A., Stoll S., Whaley K. B. (2020). Exploiting Chemistry and Molecular Systems for Quantum Information Science. Nat. Rev. Chem.

[cit3] Gruber A., Dräbenstedt A., Tietz C., Fleury L., Wrachtrup J., Borczyskowski C. von. (1997). Scanning Confocal Optical Microscopy and Magnetic Resonance on Single Defect Centers. Science.

[cit4] Casola F., van der Sar T., Yacoby A. (2018). Probing Condensed Matter Physics with Magnetometry Based on Nitrogen-Vacancy Centres in Diamond. Nat. Rev. Mater..

[cit5] Seo H., Falk A. L., Klimov P. V., Miao K. C., Galli G., Awschalom D. D. (2016). Quantum Decoherence Dynamics of Divacancy Spins in Silicon Carbide. Nat. Commun..

[cit6] Awschalom D. D., Hanson R., Wrachtrup J., Zhou B. B. (2018). Quantum Technologies with Optically Interfaced Solid-State Spins. Nat. Photonics.

[cit7] Bourassa A., Anderson C. P., Miao K. C., Onizhuk M., Ma H., Crook A. L., Abe H., Ul-Hassan J., Ohshima T., Son N. T., Galli G., Awschalom D. D. (2020). Entanglement and Control of Single Nuclear Spins in Isotopically Engineered Silicon Carbide. Nat. Mater..

[cit8] Kucsko G., Maurer P. C., Yao N. Y., Kubo M., Noh H. J., Lo P. K., Park H., Lukin M. D. (2013). Nanometre-Scale Thermometry in a Living Cell. Nature.

[cit9] Neumann P., Jakobi I., Dolde F., Burk C., Reuter R., Waldherr G., Honert J., Wolf T., Brunner A., Shim J. H., Suter D., Sumiya H., Isoya J., Wrachtrup J. (2013). High-Precision Nanoscale Temperature Sensing Using Single Defects in Diamond. Nano Lett..

[cit10] Doherty M. W., Struzhkin V. V., Simpson D. A., McGuinness L. P., Meng Y., Stacey A., Karle T. J., Hemley R. J., Manson N. B., Hollenberg L. C. L., Prawer S. (2014). Electronic Properties and Metrology Applications of the Diamond NV^−^ Center under Pressure. Phys. Rev. Lett..

[cit11] Balasubramanian G., Chan I. Y., Kolesov R., Al-Hmoud M., Tisler J., Shin C., Kim C., Wojcik A., Hemmer P. R., Krueger A., Hanke T., Leitenstorfer A., Bratschitsch R., Jelezko F., Wrachtrup J. (2008). Nanoscale Imaging Magnetometry with Diamond Spins under Ambient Conditions. Nature.

[cit12] Pelliccione M., Jenkins A., Ovartchaiyapong P., Reetz C., Emmanouilidou E., Ni N., Bleszynski Jayich A. C. (2016). Scanned Probe Imaging of Nanoscale Magnetism at Cryogenic Temperatures with a Single-Spin Quantum Sensor. Nat. Nanotechnol..

[cit13] Thiel L., Wang Z., Tschudin M. A., Rohner D., Gutiérrez-Lezama I., Ubrig N., Gibertini M., Giannini E., Morpurgo A. F., Maletinsky P. (2019). Probing Magnetism in 2D Materials at the Nanoscale with Single-Spin Microscopy. Science.

[cit14] Dolde F., Fedder H., Doherty M. W., Nöbauer T., Rempp F., Balasubramanian G., Wolf T., Reinhard F., Hollenberg L. C. L., Jelezko F., Wrachtrup J. (2011). Electric-Field Sensing Using Single Diamond Spins. Nat. Phys..

[cit15] Michl J., Steiner J., Denisenko A., Bülau A., Zimmermann A., Nakamura K., Sumiya H., Onoda S., Neumann P., Isoya J., Wrachtrup J. (2019). Robust and Accurate Electric Field Sensing with Solid State Spin Ensembles. Nano Lett..

[cit16] Bian K., Zheng W., Zeng X., Chen X., Stöhr R., Denisenko A., Yang S., Wrachtrup J., Jiang Y. (2021). Nanoscale Electric-Field Imaging Based on a Quantum Sensor and Its Charge-State Control under Ambient Condition. Nat. Commun..

[cit17] Zadrozny J. M., Niklas J., Poluektov O. G., Freedman D. E. (2015). Millisecond Coherence Time in a Tunable Molecular Electronic Spin Qubit. ACS Cent. Sci..

[cit18] Jackson C. E., Lin C.-Y., Johnson S. H., van Tol J., Zadrozny J. M. (2019). Nuclear-Spin-Pattern Control of Electron-Spin Dynamics in a Series of V(IV) Complexes. Chem. Sci..

[cit19] Yu C.-J., von Kugelgen S., Krzyaniak M. D., Ji W., Dichtel W. R., Wasielewski M. R., Freedman D. E. (2020). Spin and Phonon Design in Modular Arrays of Molecular Qubits. Chem. Mater..

[cit20] von Kugelgen S., Krzyaniak M. D., Gu M., Puggioni D., Rondinelli J. M., Wasielewski M. R., Freedman D. E. (2021). Spectral Addressability in a Modular Two Qubit System. J. Am. Chem. Soc..

[cit21] Bayliss S. L., Laorenza D. W., Mintun P. J., Kovos B. D., Freedman D. E., Awschalom D. D. (2020). Optically Addressable Molecular Spins for Quantum Information Processing. Science.

[cit22] Bayliss S. L., Deb P., Laorenza D. W., Onizhuk M., Galli G., Freedman D. E., Awschalom D. D. (2022). Enhancing Spin Coherence in Optically Addressable Molecular Qubits through Host-Matrix Control. Phys. Rev. X.

[cit23] Laorenza D. W., Freedman D. E. (2022). Could the Quantum Internet Be Comprised of Molecular Spins with Tunable Optical Interfaces?. J. Am. Chem. Soc..

[cit24] Kisgeropoulos E. C., Manesis A. C., Shafaat H. S. (2021). Ligand Field Inversion as a Mechanism to Gate Bioorganometallic Reactivity: Investigating a Biochemical Model of Acetyl CoA Synthase Using Spectroscopy and Computation. J. Am. Chem. Soc..

[cit25] Fataftah M. S., Freedman D. E. (2018). Progress towards Creating Optically Addressable Molecular Qubits. Chem. Commun..

[cit26] Reedijk J., Nieuwenhuijse B. (1972). Interpretation of E.P.R.-Spectra of Powdered Octahedral Nickel(II) Complexes with Nitrogen-Donor Ligands. Recl. Trav. Chim. Pays-Bas.

[cit27] Amrutha K., Kathirvelu V. (2022). Interpretation
of EPR and Optical Spectra of Ni(II) Ions in Crystalline Lattices at Ambient Temperature. Magn. Reson. Chem..

[cit28] Shrivastava K. N., Rubins R. S., Hutton S., Drumheller J. E. (1988). Temperature Dependence of the g Value of Ni^2+^ in ZnSiF_6_·6H_2_O. J. Chem. Phys..

[cit29] Rubins R. S., Jani S. K. (2008). Electron Paramagnetic Resonance of Divalent Nickel in Zinc Fluosilicate. J. Chem. Phys..

[cit30] Rubins R. S., Hutton S. L., Drumheller J. E. (1986). Temperature Dependence of the Zero-field Splitting of Ni^2+^ in ZnSiF_6_· 6H_2_O and ZnSiF_6_·6D_2_O at Low Temperatures. J. Chem. Phys..

[cit31] Trapp C., Shyr C. (1971). Paramagnetic Resonance in Transition-Metal Hexammine Complexes. I The Ni(II)(NH_3_)_6_ Complex Ion. J. Chem. Phys..

[cit32] Ochi J. A., Sano W., Isotani S., Hennies C. E. (1975). Phase Transition in Metal Hexammine Complexes. II. The EPR Spectra of Ni(NO_3_)_2_6NH_3_ and Ni^++^ Doped Zn(NO_3_)_2_6NH_3_ and Cd(NO_3_)_2_6NH_3_. J. Chem. Phys..

[cit33] Ochi J. A., Isotani S., Sano W. (1982). Crystal Field Distortion Parameter of Nickel Hexammine Complexes. J. Phys. Chem. Solids.

[cit34] Titiš J., Boča R., Magnetostructural D. (2010). Correlation in Nickel(II) Complexes: Reinvestigation of the Zero-Field Splitting. Inorg. Chem..

[cit35] Abragam A. and Bleaney B., Electron Paramagnetic Resonance of Transition Ions, Oxford University Press, 1970

[cit36] Coste S. C., Pearson T. J., Freedman D. E. (2019). Magnetic Anisotropy in Heterobimetallic Complexes. Inorg. Chem..

[cit37] Krzystek J., Park J.-H., Meisel M. W., Hitchman M. A., Stratemeier H., Brunel L.-C., Telser J. (2002). EPR Spectra from “EPR-Silent” Species: High-Frequency and High-Field EPR Spectroscopy of Pseudotetrahedral Complexes of Nickel(II). Inorg. Chem..

[cit38] Laorenza D. W., Kairalapova A., Bayliss S. L., Goldzak T., Greene S. M., Weiss L. R., Deb P., Mintun P. J., Collins K. A., Awschalom D. D., Berkelbach T. C., Freedman D. E. (2021). Tunable Cr^4+^ Molecular Color Centers. J. Am. Chem. Soc..

[cit39] Wojnar M. K., Laorenza D. W., Schaller R. D., Freedman D. E. (2020). Nickel(II) Metal Complexes as Optically Addressable Qubit Candidates. J. Am. Chem. Soc..

[cit40] Fataftah M. S., Bayliss S. L., Laorenza D. W., Wang X., Phelan B. T., Wilson C. B., Mintun P. J., Kovos B. D., Wasielewski M. R., Han S., Sherwin M. S., Awschalom D. D., Freedman D. E. (2020). Trigonal Bipyramidal V^3+^ Complex as an Optically Addressable Molecular Qubit Candidate. J. Am. Chem. Soc..

[cit41] Jørgensen C. K. (1958). The Interelectronic Repulsion and Partly Covalent Bonding in Transition-Group Complexes. Discuss. Faraday Soc..

[cit42] Schäffer C. E., Klixbüll Jørgensen C. (1958). The Nephelauxetic Series of Ligands Corresponding to Increasing Tendency of Partly Covalent Bonding. J. Inorg. Nucl. Chem..

[cit43] Figgis B. N. and Goodman I., Introduction to Ligand Fields, Interscience Publishers, New York, 1966

[cit44] Larsen C. B., Braun J. D., Lozada I. B., Kunnus K., Biasin E., Kolodziej C., Burda C., Cordones A. A., Gaffney K. J., Herbert D. E. (2021). Reduction of Electron Repulsion in Highly Covalent Fe-Amido Complexes Counteracts the Impact of a Weak Ligand Field on Excited-State Ordering. J. Am. Chem. Soc..

[cit45] Stein L., Boden P., Naumann R., Förster C., Niedner-Schatteburg G., Heinze K. (2022). The Overlooked NIR Luminescence of Cr(ppy)_3_. Chem. Commun..

[cit46] Martin L. Y., Sperati C. R., Busch D. H. (1977). The Spectrochemical Properties of Tetragonal Complexes of High Spin Nickel(II) Containing Macrocyclic Ligands. J. Am. Chem. Soc..

[cit47] Brik M. G. (2006). Crystal Field Analysis of the Absorption Spectra and Electron–Phonon Interaction in Ca_3_Sc_2_Ge_3_O_12_:Ni^2+^. J. Phys. Chem. Solids.

[cit48] Brik M. G., Avram N. M., Avram C. N., Rudowicz C., Yeung Y. Y., Gnutek P. (2007). Ground and Excited State Absorption of Ni^2+^ Ions in MgAl_2_O_4_: Crystal Field Analysis. J. Alloys Compd..

[cit49] Brik M. G., Avram C. N., Avram N. M. (2008). Comparative Study of Crystal Field Effects for Ni^2+^ Ion in LiGa_5_O_8_, MgF_2_ and AgCl Crystals. J. Phys. Chem. Solids.

[cit50] Mironova-Ulmane N., Brik M. G., Sildos I. (2013). Crystal Field Calculations of Energy Levels of the Ni^2+^ Ions in MgO. J. Lumin..

[cit51] Setzer W. N., Ogle C. A., Wilson G. S., Glass R. S. (1983). 1,4,7-Trithiacyclononane, a Novel Tridentate Thioether Ligand, and the Structure of Its Nickel(II), Cobalt(II), and Copper(II) Complexes. Inorg. Chem..

[cit52] Wieghardt K., Küppers H.-J., Weiss' J. (1985). Preparation and Crystal Structure of Bis(l,4,7-Trithiacyclononane)Iron(II) Bis(Hexafluorophosphate) Containing an Octahedral, Low-Spin Fe^II^S_6_ Core. Electrochemistry of [M([9]aneS_3_)_2_]^2+^ Complexes (M = Fe, Co, Ni). Inorg. Chem..

[cit53] Wilson G. S., Swanson D. D., Glass R. S. (1986). Cobalt(II) Bis(1,4,7-Trithiacyclononane): A Low-Spin Octahedral Complex. Inorg. Chem..

[cit54] Roos B. O., Taylor P. R., Sigbahn P. E. M. (1980). A Complete Active Space SCF Method (CASSCF) Using a Density Matrix Formulated Super-CI Approach. Chem. Phys..

[cit55] Angeli C., Cimiraglia R., Evangelisti S., Leininger T., Malrieu J.-P. (2001). Introduction of *n*-Electron Valence States for Multireference Perturbation Theory. J. Chem. Phys..

[cit56] Neese F., Wennmohs F., Becker U., Riplinger C. (2020). The ORCA Quantum Chemistry Program Package. J. Chem. Phys..

[cit57] Tanabe Y., Sugano S. (1954). On the Absorption Spectra of Complex Ions. I. J. Phys. Soc. Jpn..

[cit58] Dorn M., Mack K., Carrella L. M., Rentschler E., Förster C., Heinze K. (2018). Structure and Electronic Properties of an Expanded Terpyridine Complex of Nickel(II) [Ni(ddpd)_2_](BF_4_)_2_. Z. Anorg. Allg. Chem..

[cit59] Neese F., Solomon E. I. (1998). Calculation of Zero-Field Splittings, g-Values, and the Relativistic Nephelauxetic Effect in Transition Metal Complexes. Application to High-Spin Ferric Complexes. Inorg. Chem..

[cit60] Greer S. M., Gramigna K. M., Thomas C. M., Stoian S. A., Hill S. (2020). Insights into Molecular Magnetism in Metal–Metal Bonded Systems as Revealed by a Spectroscopic and Computational Analysis of Diiron Complexes. Inorg. Chem..

[cit61] El-Khatib F., Cahier B., López-Jordà M., Guillot R., Rivière E., Hafez H., Saad Z., Girerd J.-J., Guihéry N., Mallah T. (2017). Design of a Binuclear Ni(II) Complex with Large Ising-Type Anisotropy and Weak Anti-Ferromagnetic Coupling. Inorg. Chem..

[cit62] Suaud N., Rogez G., Rebilly J.-N., Bouammali M.-A., Guihéry N., Barra A.-L., Mallah T. (2020). Playing with Magnetic Anisotropy in Hexacoordinated Mononuclear Ni(II) Complexes, An Interplay Between Symmetry and Geometry. Appl. Magn. Reson..

[cit63] Maganas D., Krzystek J., Ferentinos E., Whyte A. M., Robertson N., Psycharis V., Terzis A., Neese F., Kyritsis P. (2012). Investigating Magnetostructural Correlations in the Pseudooctahedral *Trans*-[Ni^II^{(OPPh_2_)(EPPh_2_)N}_2_(Sol)_2_] Complexes (E = S, Se; Sol = DMF, THF) by Magnetometry, HFEPR, and *Ab Initio* Quantum Chemistry. Inorg. Chem..

[cit64] Stoll S., Schweiger A. (2006). EasySpin, a Comprehensive Software Package for Spectral Simulation and Analysis in EPR. J. Magn. Reson..

[cit65] Manson J. L., Lapidus S. H., Stephens P. W., Peterson P. K., Carreiro K. E., Southerland H. I., Lancaster T., Blundell S. J., Steele A. J., Goddard P. A., Pratt F. L., Singleton J., Kohama Y., McDonald R. D., Sesto R. E. D., Smith N. A., Bendix J., Zvyagin S. A., Kang J., Lee C., Whangbo M.-H., Zapf V. S., Plonczak A. (2011). Structural, Electronic, and Magnetic Properties of Quasi-1D Quantum Magnets [Ni(HF_2_)(Pyz)_2_]X (Pyz = Pyrazine; X = PF_6_^−^, SbF_6_^−^) Exhibiting Ni-FHF-Ni and Ni-Pyz-Ni Spin Interactions. Inorg. Chem..

[cit66] Shrivastava K. N. (1973). Zero-Field Splittings in NiSiF_6_·6H_2_O as Electron Paramagnetic Resonance Thermometer. Chem. Phys. Lett..

[cit67] Sczaniecki P. B., Lesiak J. (1982). EPR Fine Structure of Divalent Nickel Ions in Powders and Single Crystals of Hexakis-Imidazole-Nickel(II) Nitrate. J. Magn. Reson..

[cit68] Mašlejová A., Ivaniková R., Svoboda I., Papánková B., Dlháň L., Mikloš D., Fuess H., Boča R. (2006). Structural Characterization and Magnetic Properties of Hexakis(Imidazole)Nickel(II) Bis(Formate), Bis(Chloroacetate), Bis(2-Chloropropionate) and Hexakis(1-Methyl-Imidazole)Nickel(II) Chloride Dihydrate. Polyhedron.

[cit69] Krzystek J., Ozarowski A., Telser J. (2006). Multi-Frequency, High-Field EPR as a Powerful Tool to Accurately Determine Zero-Field Splitting in High-Spin Transition Metal Coordination Complexes. Coord. Chem. Rev..

[cit70] Hartman J. A. R., Cooper S. R. (1986). Crown Thioether Chemistry. Synthetic, Structural, and Physical Studies of the Copper(II) and Copper(I) Complexes of Hexathia-18-Crown-6. J. Am. Chem. Soc..

[cit71] Reinen D., Ozarowski A., Jakob B., Pebler J., Stratemeier H., Wieghardt K., Tolksdorf I. (1987). Spectroscopic and Magnetic Properties of Pseudooctahedral Cu^2+^ and Co^2+^ Complexes with 1,4,7-Triazacyclononane and Its Monooxa and Trithia Analogs as Ligands. Inorg. Chem..

[cit72] Blake A. J., Halcrow M. A., Holder A. J., Schroder M. (1992). Nickel Thioether Chemistry: A Re-Examination of the Electrochemistry of [Ni([9]aneS_3_)_2_I^2+^]. The Single-Crystal X-Rav Structure of a Nickel(III) Thioether Complex, [Ni^III^([9]aneS_3_)_2_][H_5_O_2_]_3_[CIO_4_]_6_ ([9]aneS_3_ = 1,4,7-Trithiacyclononane). J. Chem. Soc., Dalton Trans..

[cit73] Rubins R. S. (2005). Dynamic and Static Contributions to the Zero-Field Splitting Term of Divalent Nickel in Fluosilicate Crystals. J. Phys. Chem. Solids.

[cit74] Lee J. P., Grant G. J., Noll B. C. (2011). Bis(1,4,7-Trithiacyclononane)Nickel(II)
Bis-(Tetrafluoridoborate) Nitromethane Disolvate. Acta Crystallogr. E.

[cit75] Setzer W. N., Guo Q., Grant G. J., Hubbard J. L., Glass R. S., VanDerveer D. G. (1990). 1,4,7-Trithiacyclononane as a Tridentate Ligand for Complexation of Heavy-Metal Ions: Synthesis and Complexation Studies of Mesocyclic and Macrocyclic Polythioethers IV. Heteroat. Chem..

[cit76] Manson J. L., Manson Z. E., Sargent A., Villa D. Y., Etten N. L., Blackmore W. J. A., Curley S. P. M., Williams R. C., Brambleby J., Goddard P. A., Ozarowski A., Wilson M. N., Huddart B. M., Lancaster T., Johnson R. D., Blundell S. J., Bendix J., Wheeler K. A., Lapidus S. H., Xiao F., Birnbaum S., Singleton J. (2020). Enhancing Easy-Plane Anisotropy in Bespoke Ni(II) Quantum Magnets. Polyhedron.

[cit77] Manson J. L., Curley S. P. M., Williams R. C., Walker D., Goddard P. A., Ozarowski A., Johnson R. D., Vibhakar A. M., Villa D. Y., Rhodehouse M. L., Birnbaum S. M., Singleton J. (2021). Controlling Magnetic Anisotropy in a Zero-Dimensional *S* = 1 Magnet Using Isotropic Cation Substitution. J. Am. Chem. Soc..

[cit78] Rubín-Osanz M., Lambert F., Shao F., Rivière E., Guillot R., Suaud N., Guihéry N., Zueco D., Barra A.-L., Mallah T., Luis F. (2021). Chemical Tuning of Spin Clock Transitions in Molecular Monomers Based on Nuclear Spin-Free Ni(ii). Chem. Sci..

[cit79] Zadrozny J. M., Graham M. J., Krzyaniak M. D., Wasielewski M. R., Freedman D. E. (2016). Unexpected Suppression of Spin–Lattice Relaxation *via* High Magnetic Field in a High-Spin Iron(iii) Complex. Chem. Commun..

[cit80] Knox K., Shulman R. G., Sugano S. (1963). Covalency Effects in KNiF_3_. II. Optical Studies. Phys. Rev..

[cit81] Yang R., Zompa L. J. (1976). Metal Complexes of Cyclic Triamines. 1. Complexes of 1,4,7-Triazacyclononane ([9]aneN_3_) with Nickel(II), Copper(II), and Zinc(II). Inorg. Chem..

[cit82] Vander Griend D. A., Bediako D. K., DeVries M. J., DeJong N. A., Heeringa L. P. (2008). Detailed Spectroscopic, Thermodynamic, and Kinetic Characterization of Nickel(II) Complexes with 2,2′-Bipyridine and 1,10-Phenanthroline Attained *via* Equilibrium-Restricted Factor Analysis. Inorg. Chem..

[cit83] Tanabe Y., Sugano S. (1954). On the Absorption Spectra of Complex Ions II. J. Phys. Soc. Jpn..

[cit84] Racah G. (1942). Theory of Complex Spectra. II. Phys. Rev..

[cit85] Stranger R., Wallis S. C., Gahan L. R., Kennard C. H. L., Byriel K. A. (1992). Spectroscopic and Ligand-Field Analysis of the Spin–Orbit Interaction between the ^1^E_g_ and ^3^T_2g_ States in Bis(1,4,7-Triazacyclononane)Nickel(II). J. Chem. Soc., Dalton Trans..

[cit86] Brik M. G., Camardello S. J., Srivastava A. M., Avram N. M., Suchocki A. (2016). Spin-Forbidden Transitions in the Spectra of Transition Metal Ions and Nephelauxetic Effect. ECS J. Solid State Sci. Technol..

[cit87] Wenger O. S., Bénard S., Güdel H. U. (2002). Crystal Field Effects on the Optical Absorption and Luminescence Properties of Ni^2+^-Doped Chlorides and Bromides: Crossover in the Emitting Higher Excited State. Inorg. Chem..

[cit88] Brik M. G., Srivastava A. M., Avram N. M., Suchocki A. (2014). Empirical Relation between Covalence and the Energy Position of the Ni^2+ 1^E State in Octahedral Complexes. J. Lumin..

[cit89] Basore E. T., Liu X., Qiu J. (2019). Broadband Near-IR Photoluminescence in Ni^2+^ Doped Gallium Silicate Glass–Ceramics. J. Mater. Sci.: Mater. Electron..

[cit90] Rothemund P. W. K. (2006). Folding DNA to Create Nanoscale Shapes and Patterns. Nature.

[cit91] Veneziano R., Ratanalert S., Zhang K., Zhang F., Yan H., Chiu W., Bathe M. (2016). Designer Nanoscale DNA Assemblies Programmed from the Top Down. Science.

[cit92] Wamhoff E.-C., Banal J. L., Bricker W. P., Shepherd T. R., Parsons M. F., Veneziano R., Stone M. B., Jun H., Wang X., Bathe M. (2019). Programming Structured DNA Assemblies to Probe Biophysical Processes. Annu. Rev. Biophys..

[cit93] East N. R., Dab C., Förster C., Heinze K., Reber C. (2023). Coupled Potential Energy Surfaces Strongly Impact the Lowest-Energy Spin-Flip Transition in Six-Coordinate Nickel(II) Complexes. Inorg. Chem..

