# Supplementary material for: Ligand field design enables quantum manipulation of spins in Ni2+ complexes
Source: Chem Sci. 2023 Dec 8;15(4):1374–83. doi: 10.1039/d3sc04919a (PMC10806831; doi:10.1039/d3sc04919a)
Supplement: SC-015-D3SC04919A-s001 [file SC-015-D3SC04919A-s001.pdf]

Electronic Supplementary Material (ESI) for Chemical Science.
This journal is © The Royal Society of Chemistry 2023

Supporting Information for:

Ligand Field Design Enables Quantum Manipulation of Spins in Ni^{2+} Complexes

Michael K. Wojnar,[1] Krishnendu Kundu,[2a] Arailym Kairalapova,[3] Xiaoling Wang,[2a] Andrew Ozarowski,[2a] Timothy C. Berkelbach,[3*] Stephen Hill,[2a,2b*] Danna E. Freedman[1]*

1. Department of Chemistry, Massachusetts Institute of Technology, Cambridge, Massachusetts 02139, United States
2a. National High Magnetic Field Laboratory, Tallahassee, Florida 32310, United States
2b. Department of Physics, Florida State University, Florida 32306, United States
3. Department of Chemistry, Columbia University, New York, New York 10027, United States

Table of Contents

Full Experimental Details and Supplementary Text

Table S1 | Summary of spin Hamiltonian parameters for **1** simulating cw-EPR spectra at 295/406 GHz at 3 K. 13

Table S2 | Spin–spin (T_m) and spin–lattice (T_1) relaxation times for **1'** at W-band (94 GHz) and Q-band (34 GHz), using a monoexponential decay function. 14

Table S3 | Spin–lattice (*T1*) relaxation times for **1'** at W-band (94 GHz) and 1.33 T, modelled with a biexponential decay function. 15

Table S4 | Spin–lattice (*T1*) relaxation times for **1'** at W-band (94 GHz) and 2.50 T, modelled with a biexponential decay function. 16

Table S5 | Spin–lattice (*T1*) relaxation times for **1'** at Q-band (34 GHz) and 0.25 T, modelled with a biexponential decay function.. 17

Table S6 | Spin–lattice (*T1*) relaxation times for **1'** at Q-band (34 GHz) and 0.69 T, modelled with a biexponential decay function. 18

Table S7 | **Table S7** | Löwdin orbital population analysis of CASSCF (12,12) results. Compositions are given for Ni d-orbitals and S p-orbitals. Orbital count begins at 0. 19

Table S8 | Triplet and singlet transition energies from CASSCF(12,12) with NEVPT2. Individual NEVPT2 contributions to ZFS, $D = 1.147$ cm^{-1} and $E = 0.300$ cm^{-1} ($E/D = 0.261$). 20

Table S9 | CASSCF(12,12) triplet ground- and excited-state configurations. 21

Table S10 | CASSCF(12,12) singlet excited-state configurations. 23

Table S11 | Triplet and singlet transition energies from CASSCF(8,5) with NEVPT2. Individual NEVPT2 contributions to ZFS, $D = 1.075$ cm^{-1} and $E = 0.318$ cm^{-1} ($E/D = 0.296$). 25

Table S12 | Triplet and singlet NEVPT2 transition energies and ZFS contributions of **1** and $[Ni(tacn)_2]^{2+}$ with (8,5) active space. 26

Figure S1 | Molecular structure of **1.** Gray, yellow, and green spheres represent carbon, sulfur, and nickel atoms, respectively. Counterions, hydrogen atoms, and solvent molecules have been omitted for clarity. 27

Figure S2 | Simulated (silver) versus experimental **(**dark purple) powder X-ray diffraction data for **1.** Data were collected at room temperature with pure Cu Kα1 radiation (λ = 1.5405 Å) to confirm bulk purity. 28

Figure S3 | A view of the local environment around **1** restrained within a 10 Å distance from the nickel metal center. 29

Figure S4 | Zero-field-cooled (ZFC) variable-field magnetization for a polycrystalline sample of **1**. 30

Figure S5 | Variable-temperature dc magnetic susceptibility data for **1** collected from 1.8 K to 300 K under a dc field of 1.0 T. 31

Figure S6 | Variable-temperature dc magnetic susceptibility data for **1** collected from 1.8 K to 300 K under a dc field of 1.0 T. The black lines represent the simulations obtained from the best fits to the data using the spin Hamiltonian, $\hat{H} = \mu_B H \cdot \overleftrightarrow{g} \cdot \hat{S} + D[\hat{S}_z^2 - (S(S+1))/3] + E(\hat{S}_x^2 - \hat{S}_y^2)$, in DAVE 2.0. The following parameters were used in the simulation: g_{iso} = 2.044; D = –14.89 cm^{-1}; |E| = 0 cm^{-1}. with simulation. 32

Figure S7 | Variable field magnetization data for 1 at temperatures of 1.8 K, 5.0 K, and 10.0 K from 0 T to 7 T. 33

Figure S8 | Curie-Weiss fit of $1/\chi M$ (T) data for **1,** fit from 50 K to 300 K under a dc field of 1.0 T. 34

Figure S9 | (top) Variable-temperature dc magnetic *susceptibility data* (cM**)** for 1 collected from 1.8 K to 300 K under a dc field of 0.5 T. (bottom) Zoom of Variable-temperature dc magnetic susceptibility data (χ_M) for **1**, highlighting the maximum χ_M at 2 K. 35

Figure S10 | High frequency EPR spectra for **1** at 6 K. 36

Figure S11 | High frequency cw-EPR spectra for **1** at 3 K and 6 K and 295 GHz. 37

Figure S12 | High frequency cw-EPR spectra for **1** at 3 K, 6 K, and 20 K and 406 GHz. 38

Figure S13 | Best spectral simulations of the 295.0 GHz cw-EPR spectrum of **1** at 3 K. 39

Figure S14 | Best spectral simulations of the 406.0 GHz cw-EPR spectrum of **1** at 3 K. 40

Figure S15 | Best spectral simulations of the 406.0 GHz cw-EPR spectrum of **1** with positive and negative *D* values at 3 K. 41

Figure S16 | Best spectral simulations of the 406.0 GHz cw-EPR spectrum of **1** at 3 K, with negative *D* value and the following spin Hamiltonian parameters: g_x = 2.060, g_y = 2.155, g_z = 2.078, D = –0.55 cm^{-1}, |E| = 0.05 cm^{-1}, H-strain = [3000 2000 0]. 42

Figure S17 | Zeeman diagrams for 295.0 GHz EPR simulations of **1.** The Zeeman plots are simulated with the magnetic field oriented along the x-, y-, and z-molecular axes (top, middle, bottom, respectively) highlighting the transitions (vertical lines) observed in the spectrum. The −1, 0 and +1 M_S sublevels are labelled in the high field regime. The spin Hamiltonian parameters g, D, and E used for these simulations are given in the manuscript and SI. 43

Figure S18 | Echo-detected field-swept spectrum of **1'** at 5 K, collected at 94 GHz. 44

Figure S19 | Experimental (dark purple) and calculated (silver) absorptive cw mode of **1'** at 94 GHz, simulated using the cw-EPR parameters from **Table S1** (silver). 45

Figure S20 | Experimental and calculated absorptive cw mode of **1'** at 94 GHz, simulated as two distinct species using the following cw-EPR parameters: (1) g_{iso} = 2.18, D = +1.15 cm^{-1}, |E| = 0.10 cm^{-1} (2) g_{iso} =2.15, D = +0.9 cm^{-1}, |E| = 0.05 cm^{-1} (silver). 46

Figure S21 | Experimental and calculated absorptive cw mode of **1'** at 94 GHz, simulated as two distinct species using the following cw-EPR parameters: (1, light blue) g_{iso} = 2.18, D = +1.15 cm^{-1}, |E| = 0.10 cm^{-1} (2, light pink) 47

Figure S22 | Echo-detected field-swept spectrum of **1'** at 12 K, 20 K, and 30 K, collected at 34 GHz. 48

Figure S23 | Experimental and calculated absorptive cw mode of **1'** at 34 GHz, 49

Figure S24 | Hahn-echo decay curves for **1'** at 1.33 T from 5 K to 12 K at 94 GHz. The echo decay intensities (circles) were fit (black lines) using a monoexponential function to yield the T_m times given in **Table S2**. 50

Figure S25 | Saturation-recovery data for **1'** at 1.33 T from 5 K to 12 K at 94 GHz. The saturation recovery data (circles) were fit (black lines) using a monoexponential recovery function to yield the T_1 times given in **Table S2**. 51

Figure S26 | Hahn-echo decay curves for **1'** at 2.50 T from 5 K to 10 K at 94 GHz. The echo decay intensities (circles) were fit (black lines) using a monoexponential function to yield the T_m times given in **Table S2**. 52

Figure S27 | Saturation-recovery data for **1'** at 2.50 T at 5 K and 7 K at 94 GHz. The saturation recovery data (circles) were fit (black lines) using a monoexponential recovery function to yield the T_1 times given in **Table S2**. 53

Figure S28 | Hahn-echo decay curves for **1'** at 0.25 T from 12 K to 30 K at 34 GHz. The echo decay intensities (circles) were fit (black lines) using a monoexponential function to yield the T_m times given in **Table S2**. 54

Figure S29 | Inversion-recovery data for **1'** at 0.25 T from 12 K to 30 K at 34 GHz. The inversion recovery data (circles) were fit (black lines) using a monoexponential recovery function to yield the T_1 times given in **Table S2**. 55

Figure S30 | Hahn-echo decay curves for **1'** at 0.69 T from 12 K to 30 K at 34 GHz. The echo decay intensities (circles) were fit (black lines) using a monoexponential function to yield the T_m times given in **Table S2**. 56

Figure S31 | Inversion-recovery data for **1'** at 0.69 T from 12 K to 30 K at 34 GHz. The inversion recovery data (circles) were fit (black lines) using a monoexponential recovery function to yield the T_1 times given in **Table S2**. 57

Figure S32 | Electronic absorption (UV-vis-NIR) spectrum collected in MeCN solution at room temperature for **1.** 58

Figure S33 | Schematic view of the *D* tensor axes of **1** with z-axis along the pseudo three-fold axis of the complex. 59

Experimental Details.

General Considerations. $[Ni(ttcn)_2](BF_4)_2$ (**1**) and $[Zn(ttcn)_2](BF_4)_2$ were prepared according to literature procedure.[1,2] $[Ni(H_2O)_6](BF_4)_2$ and $[Zn(H_2O)_x](BF_4)_2$ were purchased from Strem Chemicals and used as received.

$[Ni_{0.01}Zn_{0.99}(ttcn)_2](BF_4)_2$ (1'). We dissolved $[Zn(ttcn)_2](BF_4)_2$ (297 mg, 0.50 mmol, 99.0 equiv.) with $[Ni(ttcn)_2](BF_4)_2$ (3 mg, 0.005 mmol, 1.0 equiv.) in a minimal amount (10 mL) of nitromethane and layered the solution under diethyl ether to yield light pink blocks after slow diffusion of the two layers overnight. We ground these crystals into fine microcrystalline powder for EPR measurements.

Magnetic Measurements. Magnetic data were acquired on a Quantum Design MPMS-XL SQUID (**S**uperconducting **Qu**antum **I**nterference **D**evice) magnetometer. Measurements for **1** were obtained on a finely ground polycrystalline powder restrained in a frozen eicosane matrix and wrapped tightly within a gelatin capsule. Prior to full characterization, magnetization data were acquired at 100 K from 0 to 30000 Oe (3 T) to ensure the absence of curvature associated with high-temperature ferromagnetic impurities. Dc (Direct-current) susceptibility measurements were collected in the temperature range of 1.8 K– 300 K at a dc field of 10000 Oe (1 T). Dc magnetic susceptibility data were corrected for diamagnetic contributions from the sample holder and eicosane as well as for the core diamagnetism, estimated using Pascal's constants.[3] The resulting paramagnetic dc susceptibility is reported as $\chi_M T$ (cm^3 K/mol Ni^{2+}).

EPR Measurements. All high-field EPR measurements were performed at the National High Magnetic Field Laboratory (NHMFL) in Tallahassee, Florida. High-frequency, high-field measurements were performed on finely ground powders restrained with eicosane. Samples were prepared in plastic caps for measurement. High-field, high-frequency continuous-wave EPR (cw-EPR) spectra on **1** was collected on an instrument described elsewhere.[4] Simulations of these spectra were carried out using the pepper function in Easyspin.[5] At low temperatures, the apparent noise in the spectra is due to signals from individual polycrystallites giving an incomplete signal that can be reproduced by computer simulation with an insufficiently fine grid, not a low signal/noise ratio (S/N). This is shown in the less "noisy" spectra at higher temperatures.[6,7] We note that determining small ZFS can be difficult and lead to small errors with high-field high-

frequency EPR as ZFS can be of the magnitude of *g* anisotropy).[8] While the spectra can be simulated with a negative *D* value, this leads to unrealistic values of *g* (**Figure S16**). The spin-forbidden $\Delta M_S = 2$ transition was not observed, along with the corresponding double-quantum transition,[9] attributed to the negligibly small value of transverse anisotropy due to the high symmetry of the complex, in addition to the magnitude of *E* being much smaller in magnitude in comparison to the Zeeman splitting.[10,11]

Pulse EPR data at W-band frequency (94 GHz) were obtained on HiPER at the NHMFL in Tallahassee, Florida. The architecture of the HiPER spectrometer has been previously reported.[12] Quasi-optical cw measurements were performed on HiPER using plastic sample holders. All pulse measurements were collected on solid-state dilutions in their respective diamagnetic analogues. Solid-state dilutions were prepared in a 1:100 (1%) ratio to suppress the influence of intermolecular electronic spin interactions on T_m and T_1. Echo-detected field-swept EPR spectra were recorded using a two-pulse Hahn-echo sequence (π/2–τ–π–τ–echo) with microwave (mw) π/2 and π pulse lengths of 50 ns and 100 ns, respectively, and an interpulse delay time τ = 300 ns. Hahn-echo decay curves were collected by application of the above sequence at the B_1-field of maximum echo intensity at variable temperatures using 50 ns and 100 ns π/2 and π pulses, respectively, with varying τ (starting from τ = 300 ns). All T_m decay curves were fit using the following exponential decay function, $I = I_0 e^{-2\tau/T_m}$, where I_0 is the initial intensity, 2τ is the interpulse delay time, and T_m is the phase memory time. Spin–lattice relaxation times were measured at the same magnetic fields using a three-pulse saturation recovery sequence (long pulse–T–π/2–τ–π–τ–echo) with τ of 300 ns chosen to minimize the effect of electron spin echo envelope modulation by surrounding nuclei.

Pulsed Q-band EPR spectra were collected on a Bruker E580 Elexsys spectrometer, equipped with a Bruker QT-II resonator and a 300 W TWT amplifier (Applied Systems Engineering, Model 177Ka). The temperature of the cavity was maintained using a Bruker/ColdEdge FlexLine Cryostat (Model ER 4118HV-CF100). The bridge is equipped with an Arbitrary Wave Generator to create shaped pulses for increased sensitivity.

The field-swept echo detected EPR spectra and T_m measurements were obtained with a $\pi/2 - \tau_1 - \pi - \tau_1 - echo$ sequence, with T_m measurements recorded as a function of time increment, $2\tau_1$. T_1 inversion recovery experiments were obtained with a $\pi - (\tau_2 + t) - \pi/$

$2 - \tau_1 - \pi - \tau_1 - echo$ sequence and recorded as a function of time increment, t. Optimized pulses durations, based on nutation measurements and echo amplitude, were 6 ns for $\pi/2$ pulses, and 10 ns for π pulses at 30 K, and 10 ns for $\pi/2$ pulses, and 16 ns for π pulses at 12 K and 20 K. τ_1 was chosen as 100 ns, for all experiments, with t = 2 ns for T_m measurements and τ_2= 40 ns and t = 10 ns for T_1 measurements. The data were acquired with 2-step phase cycling and signal averaged until a desirable SNR was achieved. Modulations in the decay curves are attributed to instrumental vibrations. As **1'** is composed of two distinct sites, T_1 can also be effectively modelled with a biexponential decay function (**Table S*3*–Table S*6***). **1'** exhibited electron spin echo envelope modulation (ESEEM) of the echo intensity from weakly coupled ^{1}H nuclei. Due to the inherent errors in adding the necessary fit parameters to the relaxation equation to model ESEEM, we chose to ignore the ESEEM in our fits.

Powder X-Ray Diffraction. **1** was sandwiched between pieces of Kapton tape and powder X-ray diffraction patterns were collected on a STOE STADI MP diffractometer equipped with CuKα1 radiation.

Other Physical Measurements. Electronic absorption (UV-vis-NIR) spectra were collected for **1** in MeCN with a Varian Cary 5000 spectrophotometer at room temperature.

Computational Details. The positions of H-atoms in the crystal structure geometry were optimized with the B3LYP hybrid functional[13–15] and def2-TZVP[16] basis set. A significant speedup is attained using RIJCOSX which stands for the resolution of the identity[17] and chain-of-spheres[18] approximations in the Coulomb and exchange terms, respectively. A supplementary auxiliary basis set is required for RIJCOSX for which def2/J[19] was selected. Dispersion interactions were accounted for using D3BJ correction involving Becke-Johnson damping.[20,21] A single-point PBE[22] calculation was performed to obtain unrestricted natural orbitals to be used as an initial guess for the subsequent complete active space self-consistent field (CASSCF) calculation. State-averaged CASSCF(8,5) and CASSCF(12,12) were performed, 10 triplet and 15 singlet roots were extracted in each case. The second-order n-electron valence state perturbation theory (NEVPT2) in its strongly contracted flavor was performed to account for dynamic correlations. Spin-orbit coupling (SOC) and g-tensor were calculated within the relative block of CASSCF, where spin-orbit mean-field (SOMF) approximation was used to account for SOC interactions.

Discussion of electronic absorption spectroscopy data. Unlike other Ni^{2+} complexes,[23] the spin-forbidden $^3A_{2g}\rightarrow{}^1E_g$ transition is not observed, which we attribute to either the broadness of the $^3A_{2g}\rightarrow{}^3T_{2g}$ transition or the energetic separation between $^3A_{2g}\rightarrow{}^3T_{2g}$ and the $^3A_{2g}\rightarrow{}^1E_g$. The ^1E_g energy for $[Ni(ttcn)_2]^{2+}$ was previously reported at 8500 cm^{-1} and is similar to that reported for another NiS_6 complex.[24] The forbidden intraconfigurational spin-flip gains intensity via SOC to the allowed transition; this intensity-gaining mechanism is inversely proportional to the energetic separation between the states.[25] Without observance of the $^3A_{2g}\rightarrow{}^1E_g$ transition, the Racah parameter, *C*, cannot be calculated. Additionally, photoluminescence experiments on single crystals of **1** at 5 K yielded no PL, presumably due to the low extinction coefficients ($\varepsilon \sim 30 - 40$ M^{-1} cm^{-1}) associated with the d-d transitions in **1**, energy gap law non-radiative decay for the expected low energy of the spin-flip state, and the small energy gap between the $^3T_2$ and ^1E states.[26] This leads to some ambiguity in the corresponding Tanabe-Sugano diagram, which is typically constructed with set *B* and *C* parameters ($B/C = 4.71$) which lead to a singlet-triplet crossing point at 17.3 *B*. We obtain a value of parameter *C* of 2190 cm^{-1} when we use the previously obtained energy for the $^3A_{2g}\rightarrow{}^1E_g$ transition (^1E = 8500 cm^{-1}; $C/B = 3.29$).[24] With the *B* value, we obtain a ligand field splitting parameter of 19.2 (Δ_o/B) for **1**.

Discussion of SQUID magnetometry data. To initially assess the ground-state magnetic properties and EPR addressability of Ni^{2+} in this soft coordination environment, we employed SQUID magnetometry. At 300 K, **1** exhibited a $\chi_M T$ value of 1.05 cm^3 K mol^{-1}, consistent with an $S = 1$ ion (**Figure S5**).[27] The proximity of this value to that of the spin-only value $g = 2.00$ for an $S = 1$ system ($\chi_M T = 1$ cm^3 K mol^{-1}) suggests a small orbital angular momentum contribution ($g_{iso\ simulation} = 2.05$). From 300 K to 100 K, the $\chi_M T$ value remains constant, then gradually begins to decrease. We observe a precipitous drop below 30 K, with a $\chi_M T$ value of 0.51 cm^3 K mol^{-1} at 1.8 K. Typically, for a non-interacting Curie-Weiss paramagnet, we attribute the decrease in $\chi_M T$ in a dc susceptibility curve to axial ZFS, as the thermal population (kT) is below the energy difference of spin sublevels (*D*). However, this drop usually starts below 50 K, while we observe a gradual decrease beginning around 100 K in **1**. Furthermore, simulation of this $\chi_M T$ curve yields a $|D| > 12$ cm^{-1} (**Figure S6**), well outside what is expected for Ni^{2+} in near perfectly octahedral geometries, where $|D|$ ranges from 0 to 4 cm^{-1}.[28] Intermolecular forces, such as spin-spin coupling, also contribute to behavior of magnetic susceptibility curves and their departure from Curie–Weiss paramagnetic behavior. In **1,** the only spin-spin coupling we expect to contribute to the magnetism

is the dipolar and exchange coupling between Ni^{2+} centers from neighboring molecules. In **1**, the shortest Ni–Ni distance is 8.92 Å, outside of the range for strong coupling. Yet, $[Ni(ttcn)_2]^{2+}$ previously displayed exchange interactions over long distances, where the large spin density on the sulfur atoms was hypothesized to lead to the observed magnetic properties.[29,30] A Curie-Weiss fit of $1/\chi_M$ (T) to the data for **1** results in a Curie-Weiss parameter (θ_{CW}) = –3.53 K (***Figure S8***). Although θ_{CW} is a mean-field approximation of the spin-spin interactions between neighboring Ni^{2+} centers, a magnitude of roughly 3.5 K confirms that, at low temperatures (≈ 3 K), the AFM exchange interactions contribute to the spin-behavior. A plot of χ_M vs. T (**Figure S9**) reaches a maximum value at T = 2 K (χ_M = 0.28 cm^3 mol^{-1}) and decreases again until T = 1.8 K (χ_M = 0.27 cm^3 mol^{-1}). The shape of the susceptibility curve is characteristic of other Ni^{2+}-based antiferromagnets.[31] To further probe the ground state spin, we performed low temperature magnetization experiments, in which the sample was cooled and the applied field was varied from 0 T to 7 T (**Figure S7**). At 2 K, as we increased the magnetic field to 7 T, the magnetization saturates to a value of 1.80 μ_B (close to the 1.98 μ_B for an S = 1 and g = 2), further confirming the S = 1 ground state in **1**.

Discussion of the *g*-factor Calculation. 1 is an example of an elongated octahedron ($r_{axial} > r_{equatorial}; E_{d_{x^2-y^2}} > E_{d_{z^2}}$). The sign of D is in agreement with magnetostructural correlations predicting positive D values for an elongated octahedron, confirming the M_S = 0 spin sublevel is lowest in energy.[32–34] The g-factor of the system can be calculated with the following expression: $g = g_e(1 - \frac{2\zeta^{SOC}}{E_{T2_g}})$, where g_e is the value for the free electron, ζ is the metal-based one-electron SOC constant, and E_{T2g} is the energy of the T_{2g} first excited state. Using the g value we obtain from EPR and the energy of the first triplet excited state we obtain from UV-vis spectroscopy, we calculate ζ = 320 cm^{-1}. This value is ~50% of the free ion value, further illustrating the covalency in **1**. Deviations of molecular g values from that of the free electron reflect the magnitude of SOC contributions in the ground state, represented by $2\zeta^{SOC}$ in the equation.

Discussion of Pulse EPR – Phase Memory Times (T_m). We determine the phase memory time (T_m), which encompasses all processes that contribute to decoherence or phase loss, including the electron spin T_2 and inhomogeneous dephasing time T_2^*. We extract these parameters from fitting the decay of the intensity of the two-pulse Hahn-echo sequence (π/2-τ-π-τ-echo) with increasing

interpulse delay time τ at both W- and Q-band. We fit the echo decays with mono-exponential functions ($I = I_0 e^{-2\tau/T_m}$). For **1'**, T_m values decay rapidly with increasing temperatures. The T_m values at both 1.33 T and 2.50 T (94 GHz) and 0.69 T (34 GHz) are similar, with spin–spin relaxation being relatively quick at low temperatures. At 5 K, T_m is 379 ns and 392 ns at 1.33 T and 2.50 T, respectively. At 12 K, T_m is 123 ns (1.33 T; 94 GHz) and 83 ns (0.69 T; 34 GHz). These values are in agreement with the magnetically noisy spin environment around the nickel ion, due to the protons on the ttcn ligand, as well as the BF_4^- counteranions (**Figure S3**). A spin echo is not observed after 10 K–12 K at W-band. Interestingly, T_m for **1'** at 0.25 T and 34 GHz is noticeably longer, with T_m of 317 ns at 12 K. This transition along the edge of the EDFS represents a canonical orientation, where g_z is coincident to the magnetic field, B_0. This increase in phase memory as a function of orientation has previously been observed in S = ½ V^{IV} molecular qubits.[35] The values of T_1 and T_m for **1'** are in agreement with previous pulse EPR data on Ni^{2+}; we attribute differences to a variation in host matrix (1% solid-state dilution for **1'** vs. 1 mM H_20/glycerol solution).[36]

Discussion of computational data. In a CASSCF calculation, we select an active space consisting of a fixed number of electrons, m, distributed over a fixed number of orbitals, n. CASSCF (m,n) couples all possible configurations in the active space in order to accurately account for static correlation. Even in our larger 12-orbital active space, electron excitations within only five metal d-orbitals account for all significant configurations contributing to the studied low-lying excited states, highlighting metal-centered interactions.

Due to the multi-configurational nature of the CASSCF method, the magnetic sublevels are explicitly represented allowing us to estimate the zero-field splitting (ZFS) parameters. The first order contribution to ZFS is due to the spin-spin coupling (SSC) between the unpaired electrons. The total SSC contribution to ZFS at the CASSCF /NEVPT2 levels for this system is miniscule (~0.04 cm^{-1}) and is, thus, not discussed further. The second order and leading contribution to ZFS is due to the spin-orbit coupling (SOC) between the ground state and excited states. The SOC operator has one-electron and two-electron contributions, the latter of which is difficult to treat exactly. Instead, we employ the spin-orbit mean-field (SOMF) approach to account for SOC, where an effective one-electron operator is used to account for the main two-electron contributions.[37] In SOMF approach as implemented in ORCA, the Coulomb interaction is

accounted for semi-numerically only between spin-own-orbit contributions, while the exchange interaction is estimated via a mean-field approximation accounting for spin-own-orbit and spin-other-orbit contributions.[38] Whereas the ZFS arises due to the interaction in the absence of an external magnetic field, the interaction of such a field with the spin magnetic moment of an electron from the SOC gives rise to the *g*-factor.[39] The *g*-tensor is directly proportional to the SOC and is calculated using the orbital Zeeman operator and SOC along with ZFS.[38] We extract the desired parameters using the effective Hamiltonian approach which, unlike perturbation theory, yields the entire *g*-tensor and *D*-tensor.

Due to the high symmetry of **1**, the transition dipole moments are on the order of 10^{-4} D. As a result, the calculated transitions have effectively zero oscillator strength, prohibiting us from obtaining a plot of the theoretical absorption spectrum. However, the spread of the excitation energies indicates that a potential peak corresponding to $^3T_{2g}$ is slightly broader than $^3T_{1g}$. Moreover, the ^1E_g state is very close in energy to $^3T_{2g}$, which may explain why the experimental spectrum is broad for $^3T_{2g}$ and a separate ^1E_g peak is not observed (**Figure S32**).

Two parameters that describe ZFS are axial and rhombic anisotropy, *D* and *E*, respectively. *D* is the energy difference between $M_s = 0$ and the average of $M_s = \pm 1$ sublevels, while *E* is the energy difference between $M_s = \pm 1$ sublevels and their average. *D* is negative when the $M_s = \pm 1$ sublevels are stabilized compared to $M_s = 0$. By perturbation theory, excited state energies and ZFS parameters are qualitatively inversely proportional.

To further understand the nature of the excited states, we examined their wavefunction compositions. The three triplet $^3T_{2g}$ roots and three singlet $^1T_{2g}$ roots that have major contributions to *D* share the same electron configurations albeit with slightly different proportions; the signs of triplet and singlet contributions to *D* are reversed.[40] When the ground and excited triplet states couple via the *z* component of the spin-orbit operator, states with the same M_s components couple with each other, whereas *x*/*y* components lead to coupling between states that differ by ±1. Coupling does not occur between $M_s = 0$ components due to spin symmetry.[41] Thus, when $M_s = \pm 1$ components couple with each other, the ground state $M_s = \pm 1$ sublevels are stabilized, leading to negative *D* contributions. When components that differ by ±1 couple between triplet states, the ground state $M_s = 0$ is stabilized due to the interaction with $M_s = +1$ and $M_s = -1$ components, leading to positive *D* contributions. Since the singlet excited state only has $M_s = 0$ level, coupling

of states differing by ±1 leads to stabilization of $M_s = \pm 1$ sublevels of the ground state and a negative D contribution. That is why we observe opposite signs for the singlet and triplet excited states despite nearly identical configurations (**Table S8**). As an example, the third root of the $^3T_{2g}$ state (contributing –28.585 cm^{-1} to D) and the third root of the $^1T_{2g}$ state (contributing +14.234 cm^{-1} to D) both consist of > 50% orbital 2 to orbital 5 excitation and >30% orbital 1 to orbital 4 transition; orbitals 1-5 refer to **Figure 2** in the main text, from lowest to highest energy. Thus, the electron configurations play an important role when assessing the contributions to the ZFS.

We examine the magnetic anisotropy axes of the D-tensor to get further insight. As has been previously observed for other clusters, the z component of the anisotropy axis is aligned along the highest symmetry axis corresponding to the ligand-field theory.[42] **Figure S33** also illustrates the anisotropy axes of the D-tensor and indeed places the z-axis (shown coming out of the plane in blue) along the pseudo C_3 symmetry axis. The magnetic axes corresponding to the g-tensor are closely aligned with the anisotropy axes and are, thus, omitted from the plot.[43] When the z-axis of the D-tensor is aligned with the metal-ligand bond, the analysis of the $x/y/z$ components of the spin-orbit operator is more straightforward as shown by Mallah and co-workers.[44] A similarly detailed analysis of the signs of contributions to D is prohibitively complicated for **1** due to multiconfigurational nature of the wavefunctions (**Figure 2**). However, based on the signs of D and the description above, we can estimate that the first two roots of the $^3T_{2g}$ and $^1T_{2g}$ have spin-orbit coupling operators of the x/y nature and the third roots are of z spin-orbit operator component.[41] Finally, the SOC calculation allows us to estimate the g-tensor matrix. The analysis yields an isotropic g-factor, $g_{iso} = 2.16$.

Table S1 | Summary of spin Hamiltonian parameters for **1** simulating cw-EPR spectra at 295/406 GHz at 3 K.

	**1**
g_x	2.1018(15)
g_y	2.1079(15)
g_z	2.0964(14)
D (cm^{-1})	+0.555(8)
$\|E\|$ (cm^{-1})	0.072(5)
Lorentzian Broadening	25

Table S2 | Spin–spin (T_m) and spin–lattice (T_1) relaxation times for **1'** at W-band (94 GHz) and Q-band (34 GHz), using a monoexponential decay function.

	1.33 T (94 GHz)		2.50 T (94 GHz)		0.25 T (34 GHz)		0.69 T (34 GHz)	
Temperature (K)	T_m (μs)	T_1 (μs)	T_m (μs)	T_1 (μs)	T_m (μs)	T_1 (μs)	T_m (μs)	T_1 (μs)
5	0.379(5)	7.5(2)	0.392(7)	7.8(3)	–	–	–	–
7	0.264(5)	4.6(2)	0.241(3)	5.4(2)	–	–	–	–
10	0.168(4)	3.5(1)	0.187(4)	3.97(3)	–	–	–	–
12	0.123(2)	3.26(5)	–	2.55(8)	0.370(9)	1.18(3)	0.200(2)	0.708(3)
15	–	1.18(6)	–	1.84(3)	–	–	–	–
20	–	–	–	–	0.217(4)	0.63(2)	0.113(1)	0.295(1)
30	–	–	–	–	0.105(4)	0.268(4)	0.0713(6)	0.147(1)

Table S3 | Spin–lattice (T_1) relaxation times for **1'** at W-band (94 GHz) and 1.33 T, modelled with a biexponential decay function.

Temperature (K)	$T_{1,long}$ (μs)	Weight (short)	$T_{1,short}$ (μs)	Weight (short)
5	12.8(8)	0.55	2.2(2)	0.43
7	5.5(2)	0.99	0.13(3)	0.15
10	3.8(3)	0.83	0.8(6)	0.15
12	3.6(2)	0.97	0.9(4)	0.09
15	1.4(3)	0.72	0.4(4)	0.21

Table S4 | Spin–lattice (T_1) relaxation times for **1'** at W-band (94 GHz) and 2.50 T, modelled with a biexponential decay function.

Temperature (K)	$T_{1,short}$ (μs)	Weight (short)	$T_{1,long}$ (μs)	Weight (short)
5	19(2)	0.46	3.0(2)	0.54
7	8.5(4)	0.55	1.4(1)	0.41
10	4.6(1)	1.02	1.0(1)	0.11
12	2.9(2)	1.01	0.3(2)	0.11
15	3(1)	0.85	0.9(4)	0.31

Table S5 | Spin–lattice (T_1) relaxation times for **1'** at Q-band (34 GHz) and 0.25 T, modelled with a biexponential decay function.

Temperature (K)	$T_{1,short}$ (µs)	Weight (short)	$T_{1,long}$ (µs)	Weight (short)
12	1.7(3)	1.00	0.28(9)	0.22
20	0.7(1)	0.91	0.2(2)	0.13
30	0.268(4)	1.00	–	–

Table S6 | Spin–lattice (T_1) relaxation times for **1'** at Q-band (34 GHz) and 0.69 T, modelled with a biexponential decay function.

Temperature (K)	$T_{1,short}$ (μs)	Weight (short)	$T_{1,long}$ (μs)	Weight (short)
12	1.2(2)	0.38	0.50(5)	0.45
20	0.38(3)	0.63	0.20(2)	0.53
30	0.21(2)	0.53	0.100(9)	0.81

Table S7 | Löwdin orbital population analysis of CASSCF (12,12) results. Compositions are given for Ni d-orbitals and S p-orbitals. Orbital count begins at 0.

	103	104	105	106	107	108	109	110	111	112	113	114
Ni dz^2	1.3	15.8	38.9	0.8	4.4	42.0	1.5	6.6	33.8	2.9	44.1	0.0
Ni dxz	3.1	1.2	11.8	58.6	14.4	4.7	6.2	11.9	45.4	18.5	11.7	2.8
Ni dyz	0.9	13.2	23.4	0.2	30.5	31.1	4.7	9.1	11.2	26.9	24.7	16.8
Ni $d_{x^2-y^2}$	26.0	0.2	3.4	10.1	3.6	0.0	65.3	15.5	0.0	3.8	4.9	61.7
Ni d_{xy}	0.5	1.0	20.4	28.3	44.8	1.9	1.7	47.1	0.1	38.4	0.4	4.3
S pz	1.7	1.1	0.1	0.0	0.1	0.1	0.4	0.0	0.0	0.1	0.0	0.0
S px	0.1	0.0	0.0	0.0	0.0	0.0	0.0	0.0	0.0	0.0	0.0	0.0
S py	8.6	7.1	0.0	0.0	0.0	1.6	2.9	0.0	0.0	0.0	0.1	0.7
S pz	0.7	15.5	0.0	0.0	0.0	4.3	0.0	0.0	0.0	0.0	0.6	0.0
S px	0.0	0.7	0.0	0.0	0.1	0.0	0.0	0.0	0.0	0.0	0.0	0.0
S py	0.0	1.8	0.0	0.0	0.0	0.6	0.0	0.0	0.0	0.0	0.1	0.0
S pz	0.4	0.1	0.0	0.0	0.0	0.0	0.1	0.0	0.1	0.0	0.0	0.0
S px	16.3	1.7	0.0	0.0	0.0	0.8	4.1	0.0	0.0	0.0	0.3	0.4
S py	0.1	0.0	0.0	0.0	0.1	0.0	0.0	0.0	0.0	0.0	0.0	0.0
S pz	1.7	1.1	0.1	0.0	0.1	0.1	0.4	0.0	0.0	0.1	0.0	0.0
S px	0.1	0.0	0.0	0.0	0.0	0.0	0.0	0.0	0.0	0.0	0.0	0.0
S py	8.6	7.1	0.0	0.0	0.0	1.6	2.9	0.0	0.0	0.0	0.1	0.7
S pz	0.7	15.5	0.0	0.0	0.0	4.3	0.0	0.0	0.0	0.0	0.6	0.0
S px	0.0	0.7	0.0	0.0	0.1	0.0	0.0	0.0	0.0	0.0	0.0	0.0
S py	0.0	1.8	0.0	0.0	0.0	0.6	0.0	0.0	0.0	0.0	0.1	0.0
S pz	0.4	0.1	0.0	0.0	0.0	0.0	0.1	0.0	0.1	0.0	0.0	0.0
S px	16.3	1.7	0.0	0.0	0.0	0.8	4.1	0.0	0.0	0.0	0.3	0.4
S py	0.1	0.0	0.0	0.0	0.1	0.0	0.0	0.0	0.0	0.0	0.0	0.0

Table S8 | Triplet and singlet transition energies from CASSCF(12,12) with NEVPT2. Individual NEVPT2 contributions to ZFS, $D = 1.147\ \mathrm{cm}^{-1}$ and $E = 0.300\ \mathrm{cm}^{-1}$ ($E/D = 0.261$).

Multiplicity	Root	CASSCF (cm^{-1})	NEVPT2 (cm^{-1})	State	D (cm^{-1})	E (cm^{-1})
3	1	11441.6	11966.3	$^3T_{2g}$	15.217	15.306
	2	11559.4	12087.7		14.892	–15.014
	3	11846.9	12421.0		–28.585	0.149
	4	18704.7	19057.4	$^3T_{1g}$	0.000	0.003
	5	18772.6	19117.2		0.005	0.004
	6	19055.3	19449.2		0.000	–0.002
	7	31086.4	30124.9	$^3T_{1g}$	0.000	–0.000
	8	31370.9	30412.2		0.000	0.001
	9	32519.9	31885.7		0.000	0.000
1	0	14666.0	11946.3	^1E_g	–0.001	0.001
	1	14674.9	11952.8		–0.002	0.000
	2	23523.0	19548.6	$^1A_{1g}$	0.082	–0.025
	3	26587.5	24796.7	$^1T_{2g}$	–7.344	–7.341
	4	26778.3	24994.2		–7.281	7.270
	5	27141.2	25437.4		14.234	–0.014
	6	31247.5	28998.6	$^1T_{1g}$	–0.004	–0.006
	7	31411.8	29187.7		0.010	–0.000
	8	31634.9	29447.1		–0.003	–0.003
	9	43159.5	42714.2	$^1T_{2g}$	–0.024	0.025
	10	43193.4	42698.4		0.204	–0.003
	11	43267.1	42787.6		0.110	–0.014
	12	44281.2	44054.2	^1E_g	–0.149	0.129
	13	44309.2	44074.6		–0.162	–0.143
	14	57716.4	43101.5	$^1A_{1g}$	0.001	0.000

Table S9 | CASSCF(12,12) triplet ground- and excited-state configurations. The configurations are distinguished based on singly-occupied orbitals 1-5 (105-109 in **Table S7**); the remaining orbitals are doubly-occupied. Only the configurations that contribute more than 10% of the weight are listed.

Root	CASSCF (cm^{-1})	Weight	1	2	3	4	5	State
0	0	0.95167				1	1	$^3A_{2g}$
		0.42750			1		1	
1	11441.6	0.25202		1			1	
		0.21745	1			1		
2	11559.4	0.41316	1				1	$^3T_{2g}$
		0.41117			1	1		
3	11846.9	0.60571		1		1		
		0.30191	1				1	
		0.39815		1			1	
		0.13165	1		1			
4	18704.7	0.12835	1	1				
		0.12320			1		1	
		0.10819		1	1			
		0.46929	1			1		
5	18772.6	0.20102	1	1				$^3T_{1g}$
		0.14632	1		1			
		0.27394		1	1			
		0.18973			1	1		
6	19055.3	0.15042		1		1		
		0.14358	1				1	
		0.11100	1		1			
7	31086.4	0.29686		1	1			$^3T_{1g}$

		0.26364	1		1		
		0.11056			1	1	
		0.31101	1		1		
8	31370.9	0.27405		1	1		
		0.11492			1		1
		0.61465	1	1			
9	32519.9	0.18208	1			1	
		0.15544		1			1

Table S10 | CASSCF(12,12) singlet excited-state configurations. The configurations are distinguished based on empty (-) and singly-occupied (↿/⇂) orbitals 1-5 (105-109 in **Table S7**); the remaining orbitals are doubly-occupied. Only the configurations that contribute more than 10% of the weight are listed.

Root	CASSCF (cm^{-1})	Weight	1	2	3	4	5	State
0	14666.0	0.41746				↿	⇂	^1E_g
		0.28199					-	
		0.24521				-		
1	14674.9	0.52709				⇂	↿	
		0.21306					-	
		0.20455				-		
2	23523.0	0.45352				-		$^1A_{1g}$
		0.40935					-	
3	26587.5	0.41253			↿		⇂	$^1T_{2g}$
		0.23732		↿			⇂	
		0.19942	↿			⇂		
4	26778.3	0.39299			↿	⇂		
		0.35641	↿				⇂	
5	27141.2	0.54724		↿		⇂		
		0.33376	↿				⇂	
6	31247.5	0.42180			↿		⇂	$^1T_{1g}$
		0.41999		↿			⇂	
7	31411.8	0.43672			↿	⇂		
		0.25691		↿		⇂		
		0.21136	↿				⇂	
8	31634.9	0.62355	↿			⇂		
		0.28403		↿			⇂	

9	43159.5	0.63402		↿	⇂			
		0.30214	↿		⇂			
10	43193.4	0.37961			-			
		0.21337	↿		⇂			
		0.13042		↿	⇂			$^1T_{2g}$
		0.10250		-				
11	43267.1	0.39389	↿		⇂			
		0.24631			-			
		0.16025		↿	⇂			
12	44281.2	0.40942		-				
		0.40890	-					^1E_g
		0.10411	↿	⇂				
13	44309.2	0.77786	↿	⇂				
14	57716.4	0.47036	*					$^1A_{1g}$
		0.46273						

* Singlet 14 root has single occupancies in the Ni-S π-bonding orbitals (103 and 104 in **Table S7**)

$\pi 1$	$\pi 2$	1	2	3	4	5
	↿					⇂
↿					⇂	

Table S11 | Triplet and singlet transition energies from CASSCF(8,5) with NEVPT2. Individual NEVPT2 contributions to ZFS, $D = 1.075$ cm^{-1} and $E = 0.318$ cm^{-1} ($E/D = 0.296$).

Multiplicity	Root	CASSCF (cm^{-1})	NEVPT2 (cm^{-1})	State	D (cm^{-1})	E (cm^{-1})
	1	8215.2	14179.0		13.935	13.835
	2	8304.3	14334.0	$^3T_{2g}$	13.823	–13.630
	3	8562.9	14750.4		–26.426	0.204
	4	14153.9	23185.7		0.004	0.006
3	5	14340.3	23453.7	$^3T_{1g}$	0.001	–0.001
	6	14448.5	23452.0		0.001	–0.001
	7	28855.4	33957.2		0.000	0.000
	8	29067.3	34252.3	$^3T_{1g}$	0.001	0.001
	9	29766.9	35622.8		0.000	0.000
	0	17325.3	15326.0	^1E_g	–0.001	0.001
	1	17335.8	15338.8		–0.001	0.000
	2	25223.2	29462.2		–6.198	–6.175
	3	25384.7	29689.3	$^1T_{2g}$	–6.084	6.096
	4	25635.3	30190.4		11.944	0.014
	5	28157.9	27246.7	$^1A_{1g}$	–0.001	–0.002
	6	31658.2	35069.3		0.001	–0.001
1	7	31777.8	35233.0	$^1T_{1g}$	0.003	0.000
	8	32019.7	35586.8		–0.001	0.000
	9	38083.4	46689.2		–0.055	0.056
	10	38146.8	46769.2	$^1T_{2g}$	–0.021	–0.039
	11	38453.5	46742.2		0.991	0.005
	12	39206.2	48099.7	^1E_g	–0.397	0.337
	13	39241.9	48105.3		–0.426	–0.366
	14	70724.7	70594.3	$^1A_{1g}$	0.000	0.000

Table S12 | Triplet and singlet NEVPT2 transition energies and ZFS contributions of **1** and $[Ni(tacn)_2]^{2+}$ with (8,5) active space.

Multiplicity	Root	State	**1** (cm^{-1})	$[Ni(tacn)_2]^{2+}$ (cm^{-1})	*D* **1** (cm^{-1})	*D* $[Ni(tacn)_2]^{2+}$ (cm^{-1})
3	1	$^3T_{2g}$	14179.0	13252.3	13.935	13.738
	2		14334.0	13408.3	13.823	7.447
	3		14750.4	13630.6	–26.426	–16.272
	4	$^3T_{1g}$	23185.7	20540.1	0.004	0.003
	5		23453.7	22580.4	0.001	0.002
	6		23452.0	22800.4	0.001	0.009
	7	$^3T_{1g}$	33957.2	33128.1	0.000	0.000
	8		34252.3	33276.1	0.001	–0.000
	9		35622.8	35617.8	0.000	0.000
1	0	^1E_g	15326.0	16216.4	–0.001	–0.006
	1		15338.8	16245.4	–0.001	–0.006
	2	$^1T_{2g}$	29462.2	28915.3	–6.198	–6.583
	3		29689.3	29113.9	–6.084	–6.272
	4		30190.4	29381.9	11.944	12.354
	5	$^1A_{1g}$	27246.7	28985.2	–0.001	0.324
	6	$^1T_{1g}$	35069.3	35035.7	0.001	–0.037
	7		35233.0	35343.7	0.003	–0.056
	8		35586.8	35467.4	–0.001	–0.006
	9	$^1T_{2g}$	46689.2	45451.7	–0.055	–0.053
	10		46769.2	45502.3	–0.021	–0.043
	11		46742.2	45565.1	0.991	0.928
	12	^1E_g	48099.7	46797.6	–0.397	–0.442
	13		48105.3	46817.6	–0.426	–0.446
	14	$^1A_{1g}$	70594.3	70939.5	0.000	0.002

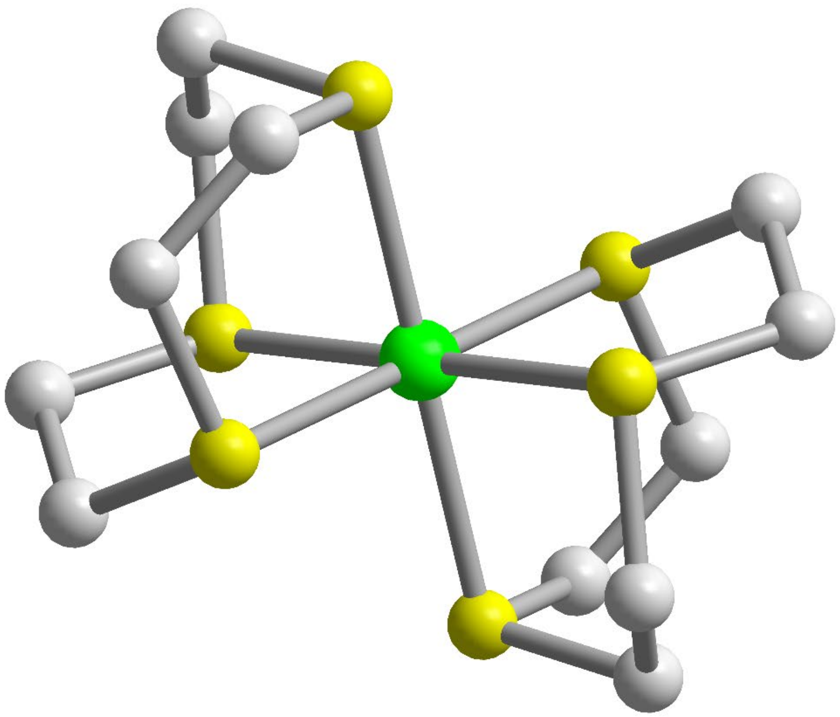

Figure S1 | Molecular structure of **1**. Gray, yellow, and green spheres represent carbon, sulfur, and nickel atoms, respectively. Counterions, hydrogen atoms, and solvent molecules have been omitted for clarity.

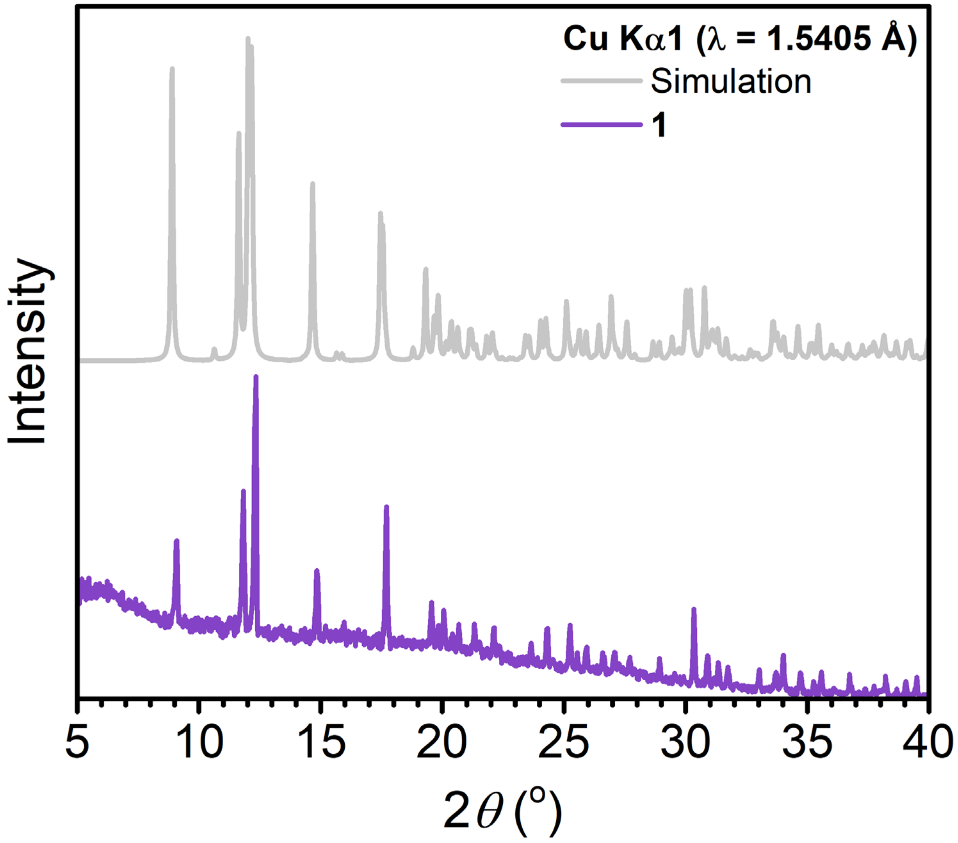

Figure S2 | Simulated (silver) versus experimental (dark purple) powder X-ray diffraction data for **1**. Data were collected at room temperature with pure Cu Kα1 radiation (λ = 1.5405 Å) to confirm bulk purity. Simulation of powder X-ray diffraction data was based on single crystal XRD data reported in Reference 45.

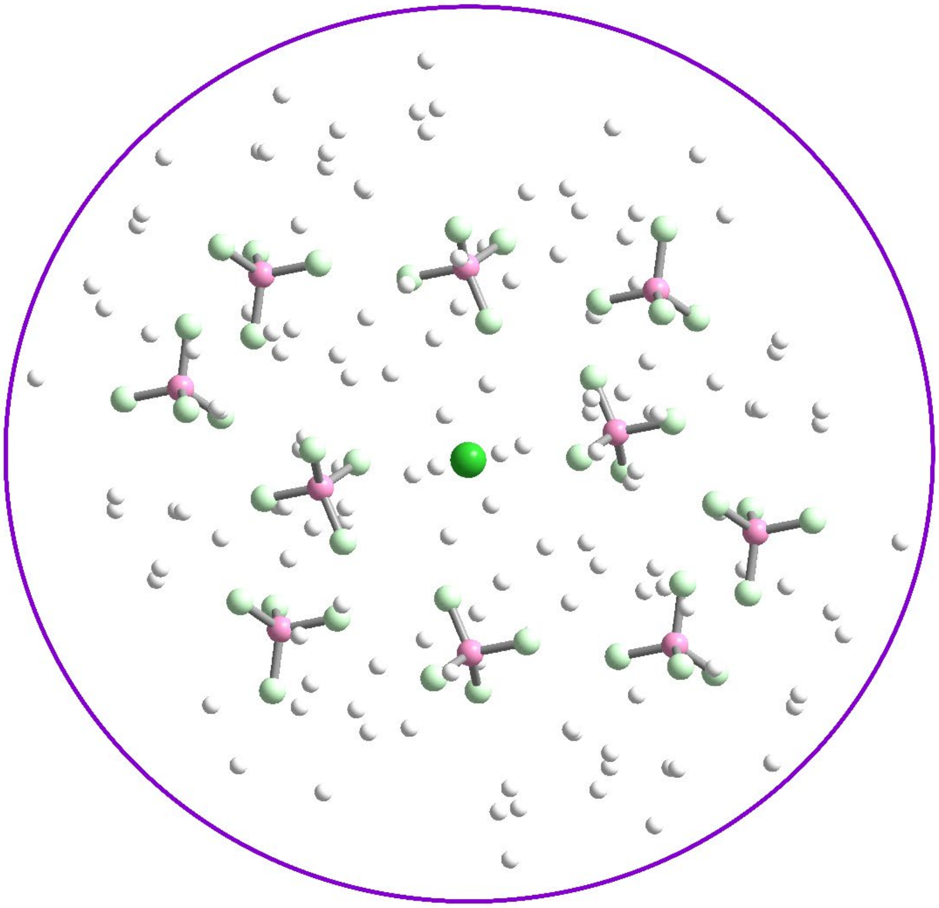

Figure S3 | A view of the local environment around **1** restrained within a 10 Å distance from the nickel metal center (dark green). The circle (solid purple line) is a guide to the eye with a radius of 10 Å. All hydrogen (white), boron (light pink), and fluorine (light green) atoms are presented within this distance.

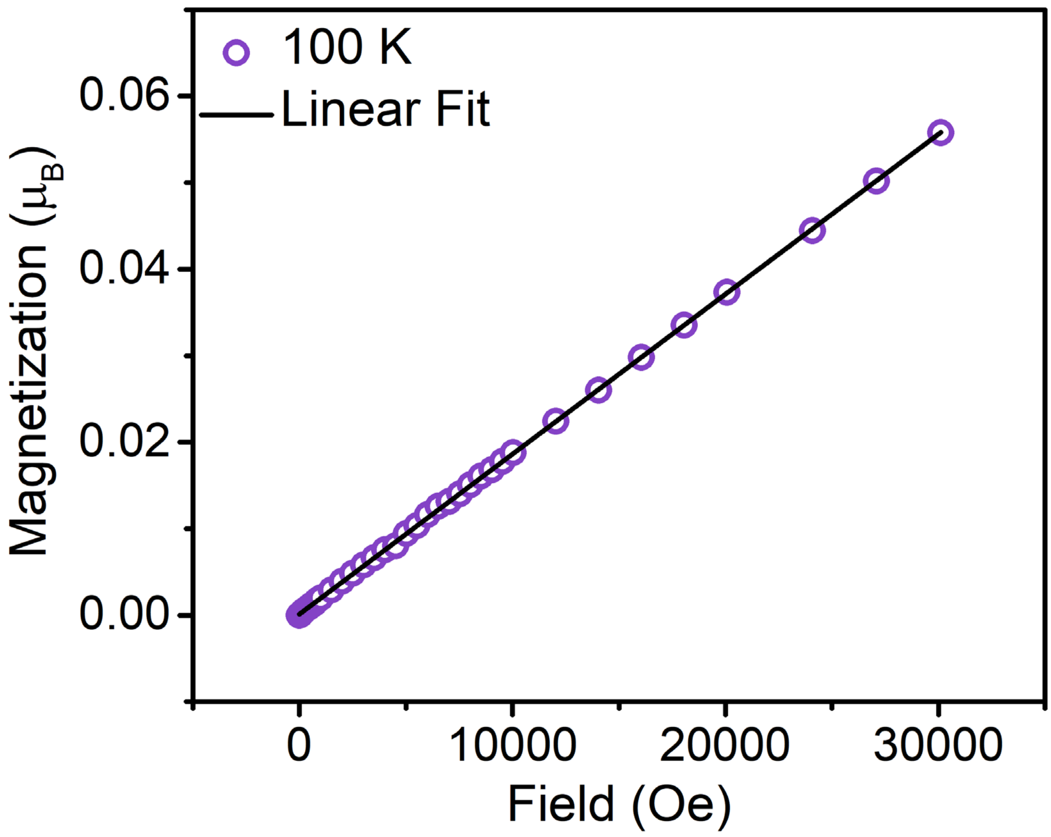

Figure S4 | Zero-field-cooled (ZFC) variable-field magnetization for a polycrystalline sample of **1** restrained under eicosane acquired at 100 K (dark purple open circles). The black line is a least-squares linear fit to the data illustrating the absence of high-temperature ferromagnetic impurities.

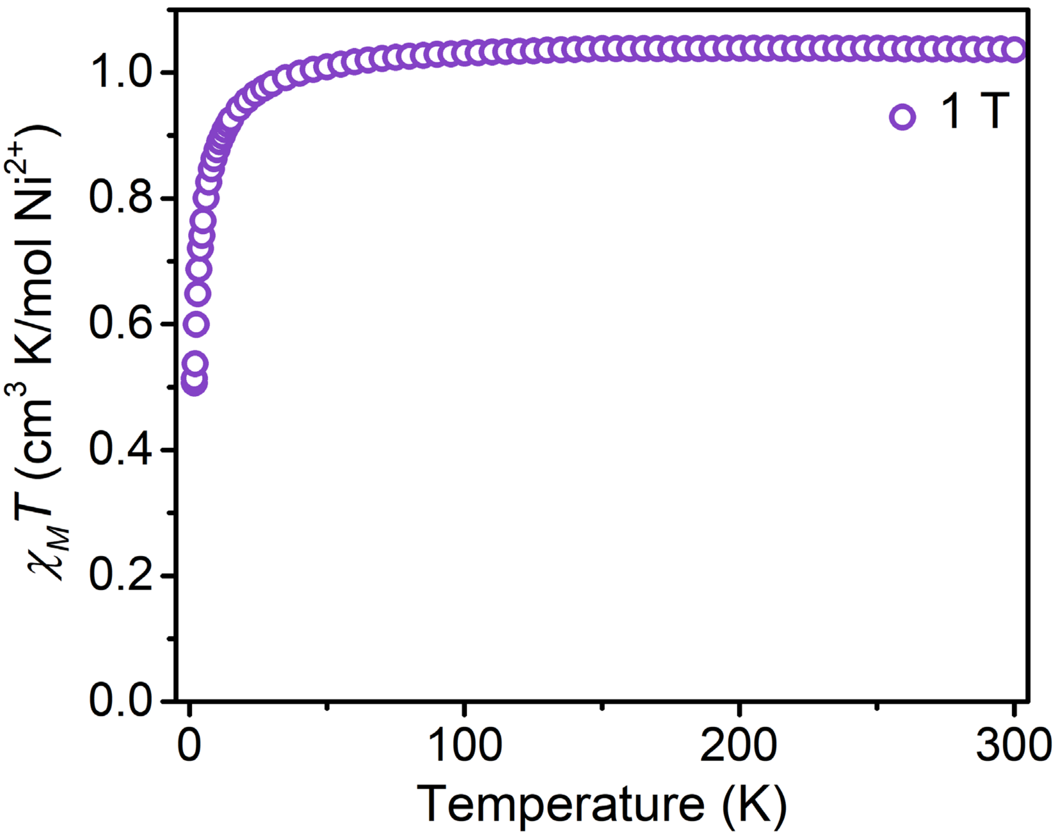

Figure S5 | Variable-temperature dc magnetic susceptibility data for **1** collected from 1.8 K to 300 K under a dc field of 1.0 T.

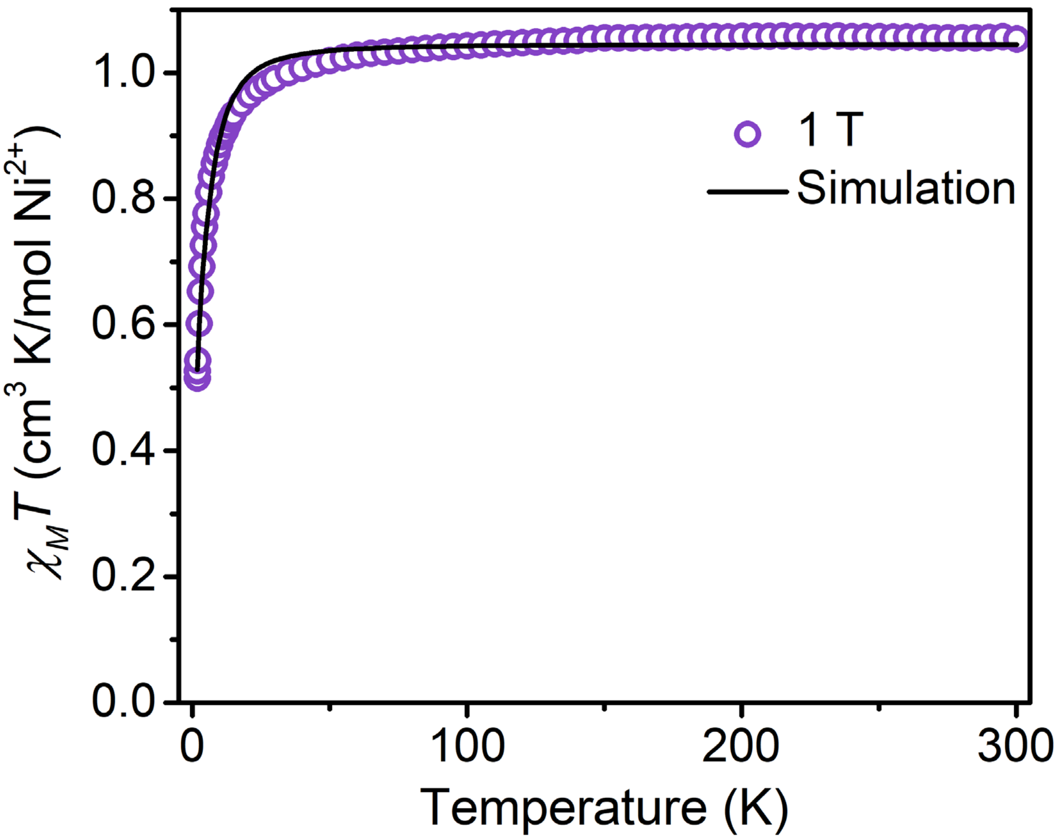

Figure S6 | Variable-temperature dc magnetic susceptibility data for **1** collected from 1.8 K to 300 K under a dc field of 1.0 T. The black lines represent the simulations obtained from the best fits to the data using the spin Hamiltonian, $\widehat{H} = \mu_B \boldsymbol{H} \cdot \overleftrightarrow{\boldsymbol{g}} \cdot \widehat{\boldsymbol{S}} + D[\hat{S}_z^2 - (S(S+1))/3] + E(\hat{S}_x^2 - \hat{S}_y^2)$, in DAVE 2.0. The following parameters were used in the simulation: g_{iso} = 2.044; D = –14.89 cm^{-1}; $|E|$ = 0 cm^{-1}.

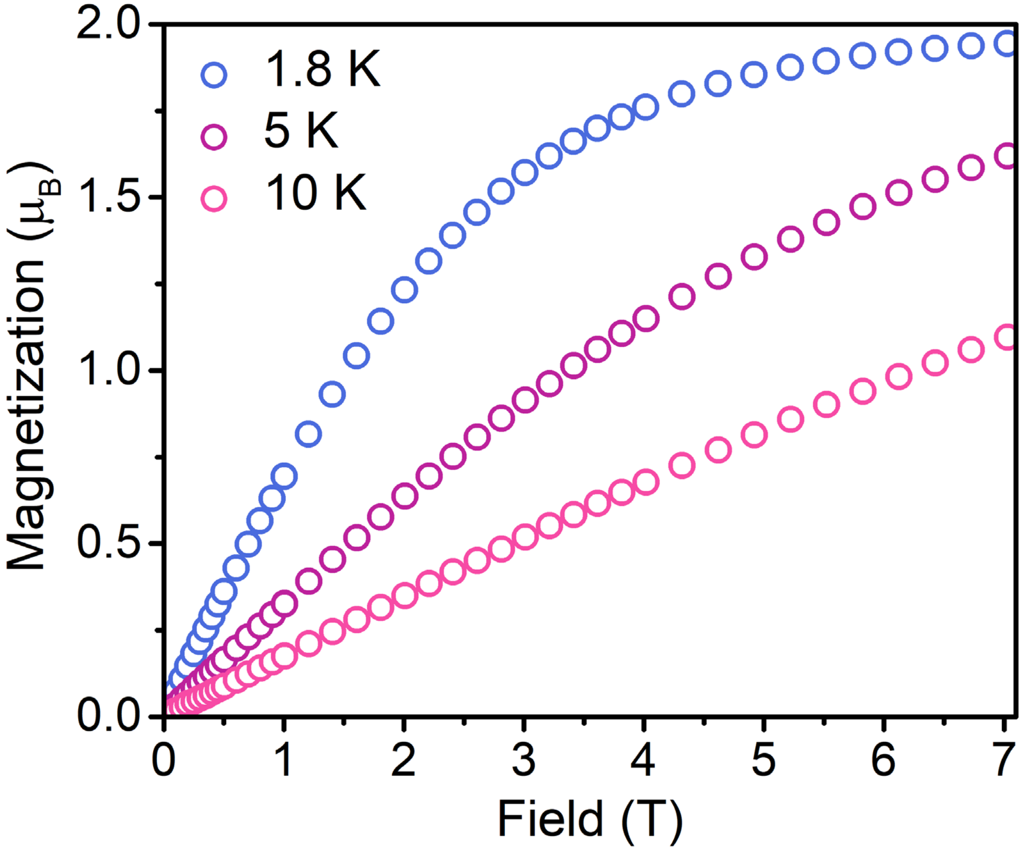

Figure S7 | Variable field magnetization data for **1** at temperatures of 1.8 K, 5.0 K, and 10.0 K from 0 T to 7 T.

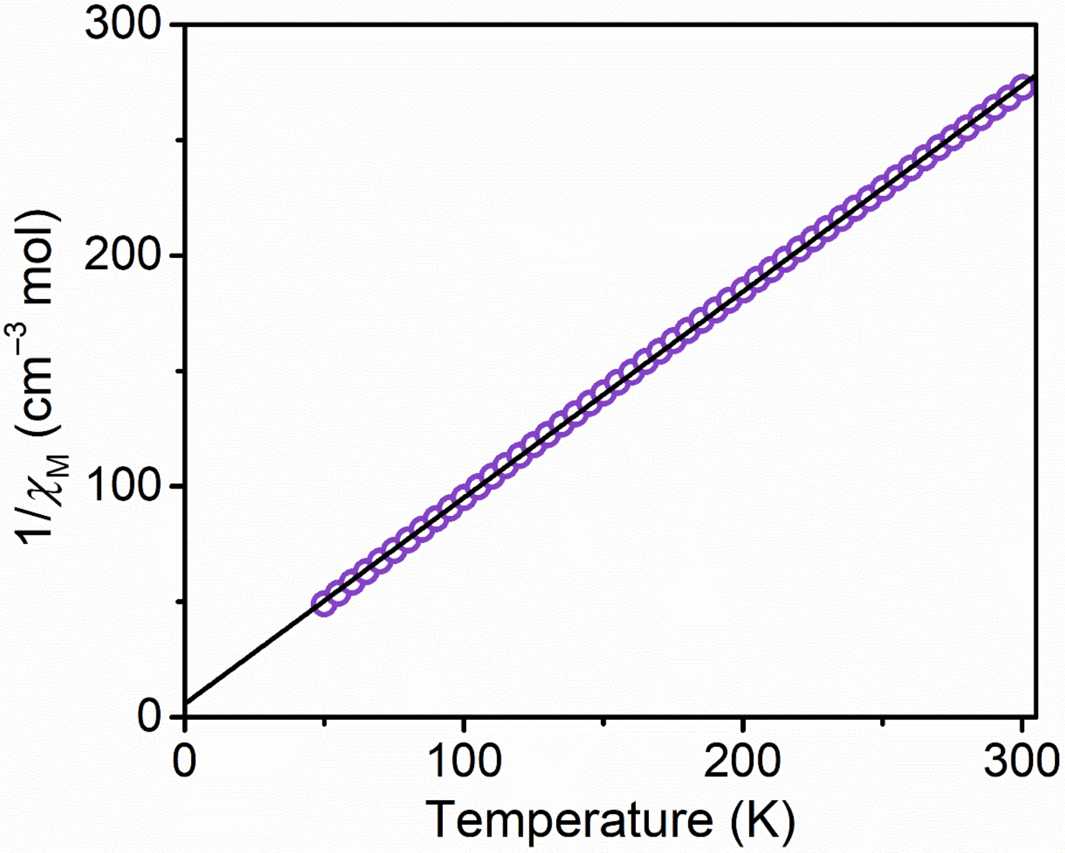

Figure S8 | Curie-Weiss fit of $1/\chi_M$ (T) data for **1**, fit from 50 K to 300 K under a dc field of 1.0 T.

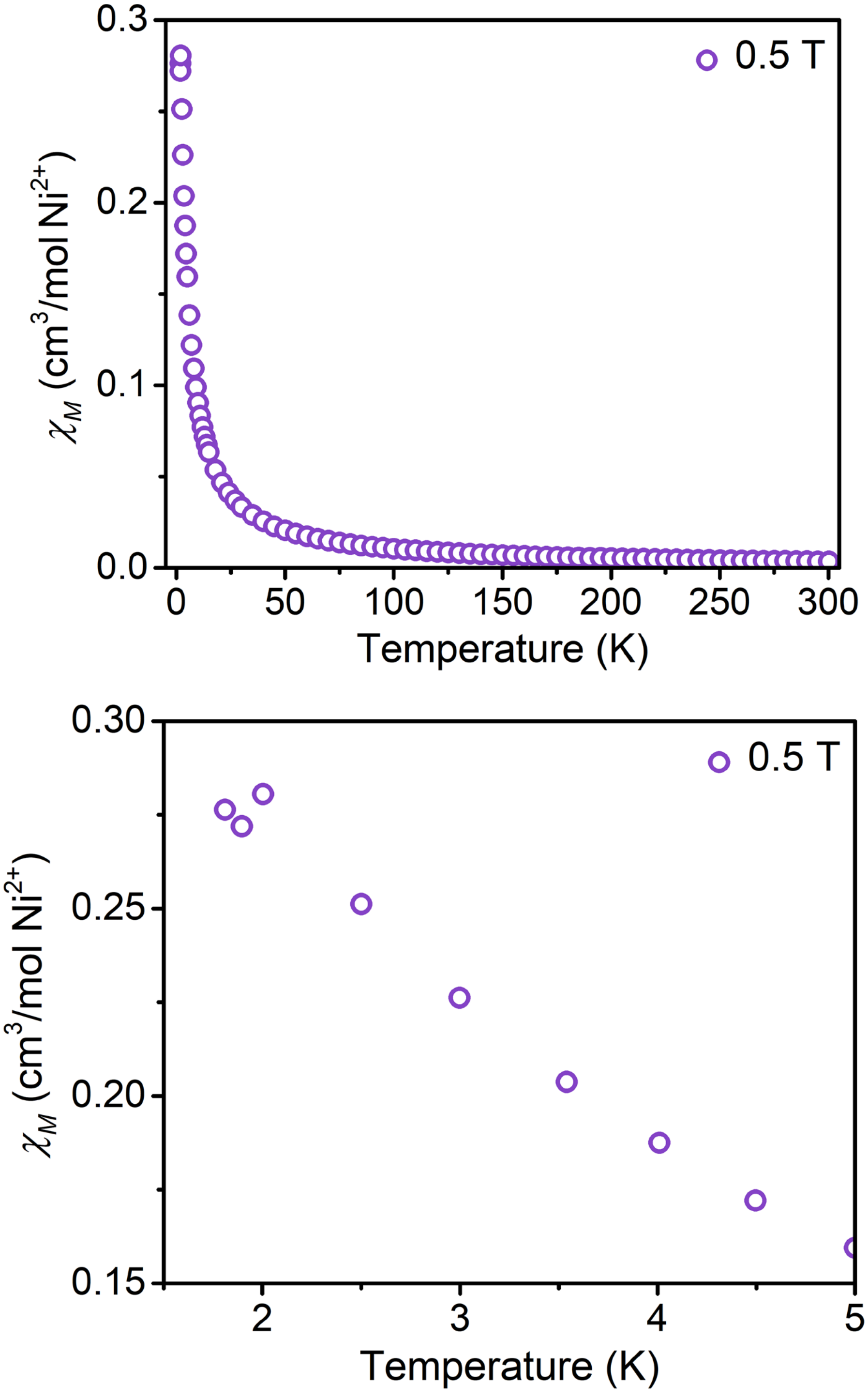

Figure S9 | (top) Variable-temperature dc magnetic susceptibility data (χ_M) for **1** collected from 1.8 K to 300 K under a dc field of 0.5 T. (bottom) Zoom of Variable-temperature dc magnetic susceptibility data (χ_M) for **1**, highlighting the maximum χ_M at 2 K.

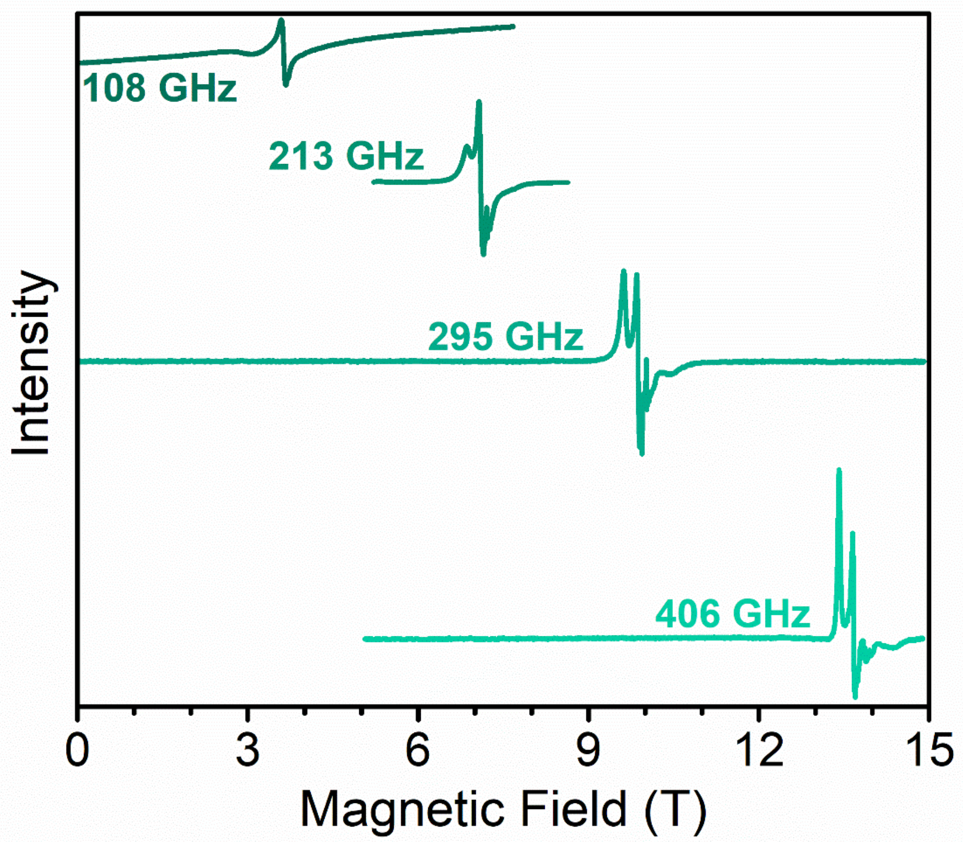

Figure S10 | High frequency EPR spectra for **1** at 6 K.

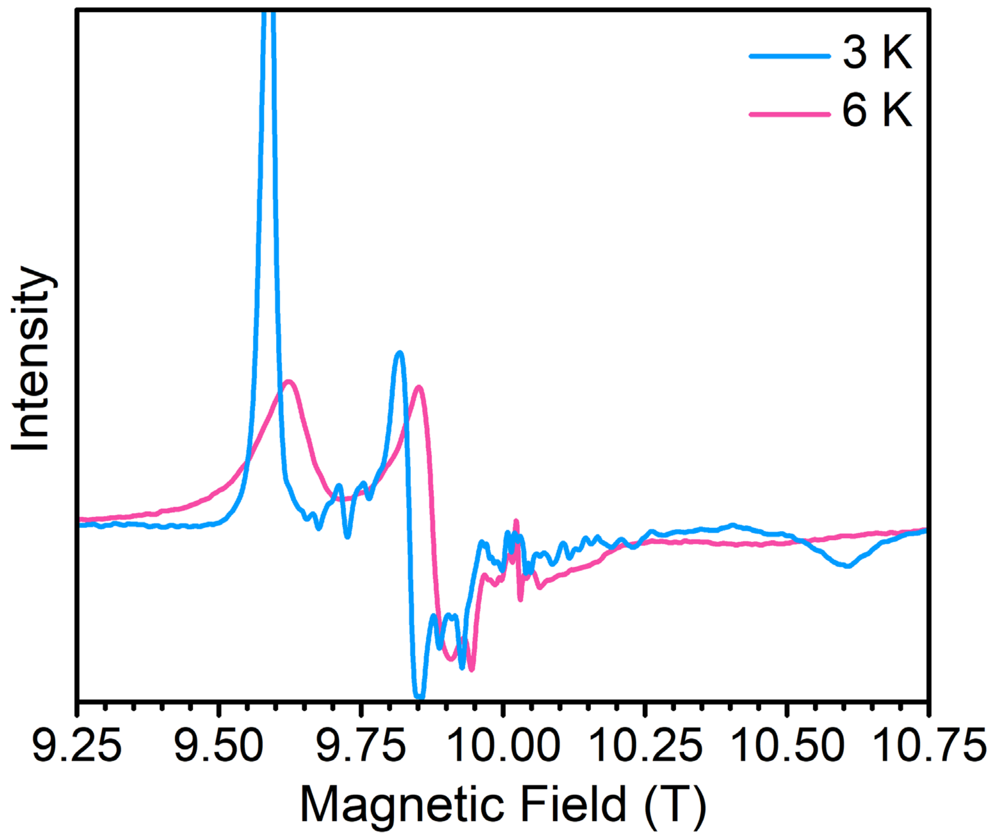

Figure S11 | High frequency cw-EPR spectra for **1** at 3 K and 6 K and 295 GHz.

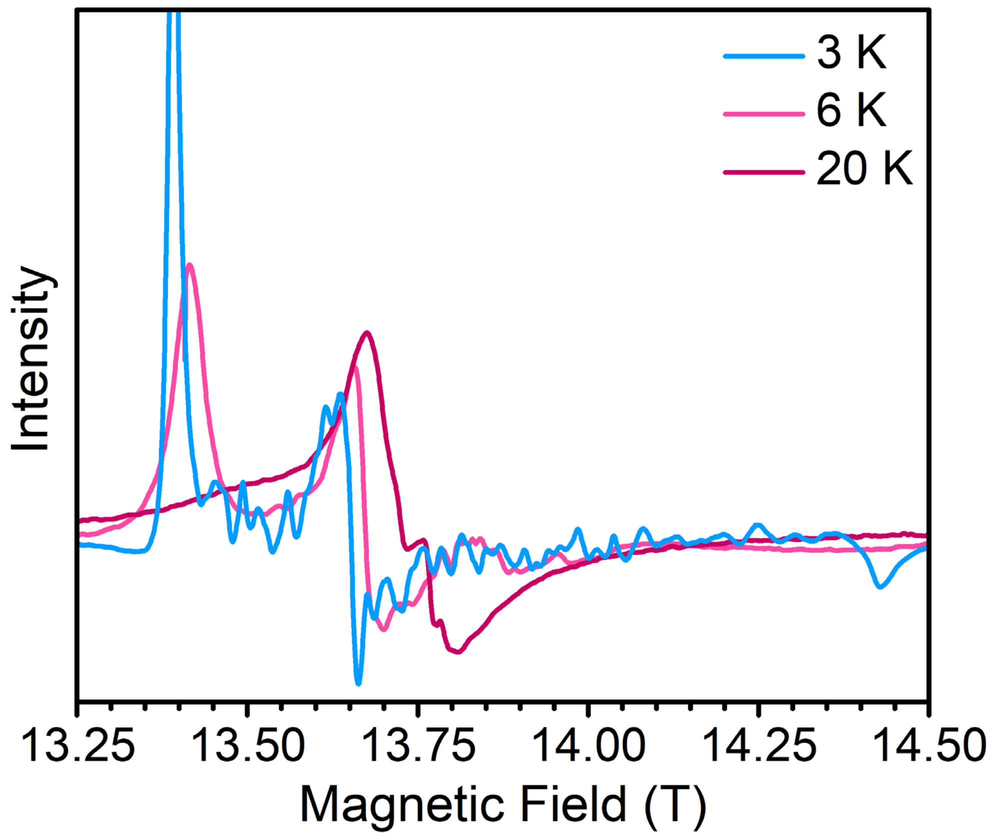

Figure S12 | High frequency cw-EPR spectra for **1** at 3 K, 6 K, and 20 K and 406 GHz.

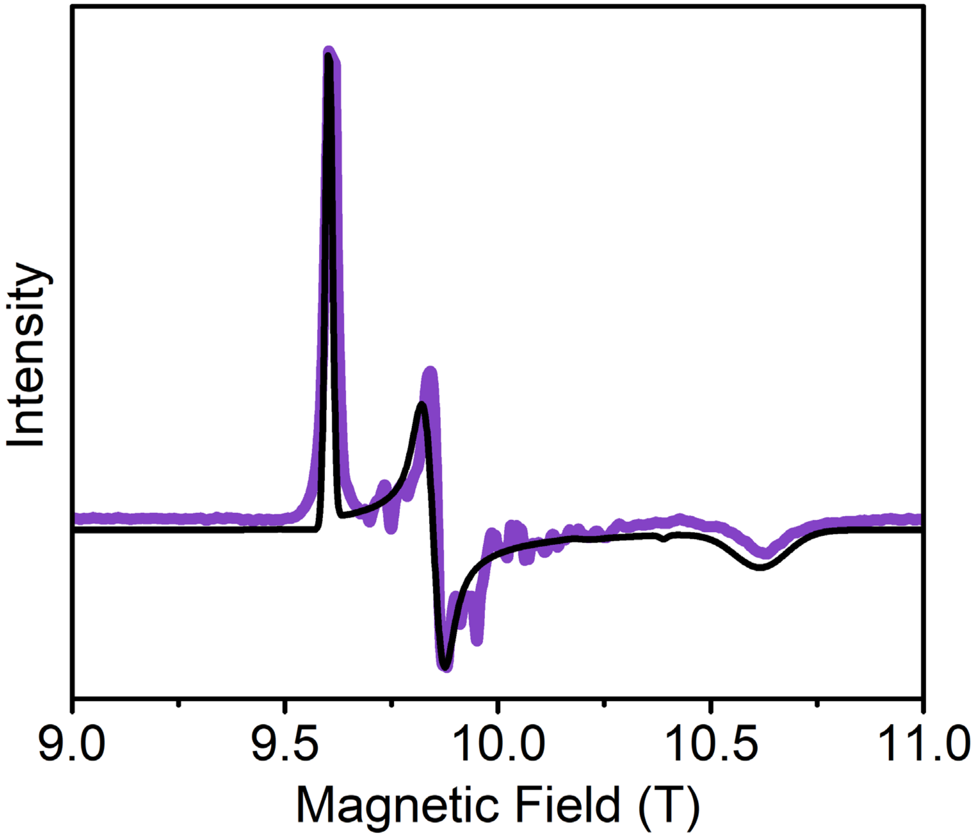

Figure S13 | Best spectral simulations of the 295.0 GHz cw-EPR spectrum of **1** at 3 K.

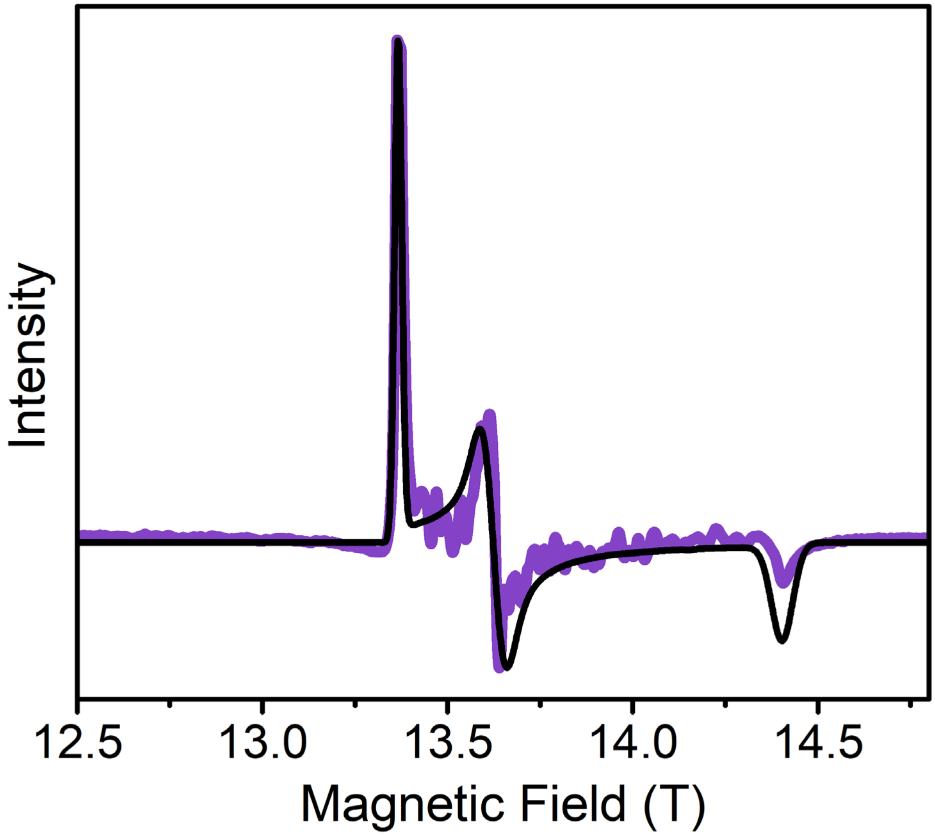

Figure S14 | Best spectral simulations of the 406.0 GHz cw-EPR spectrum of **1** at 3 K.

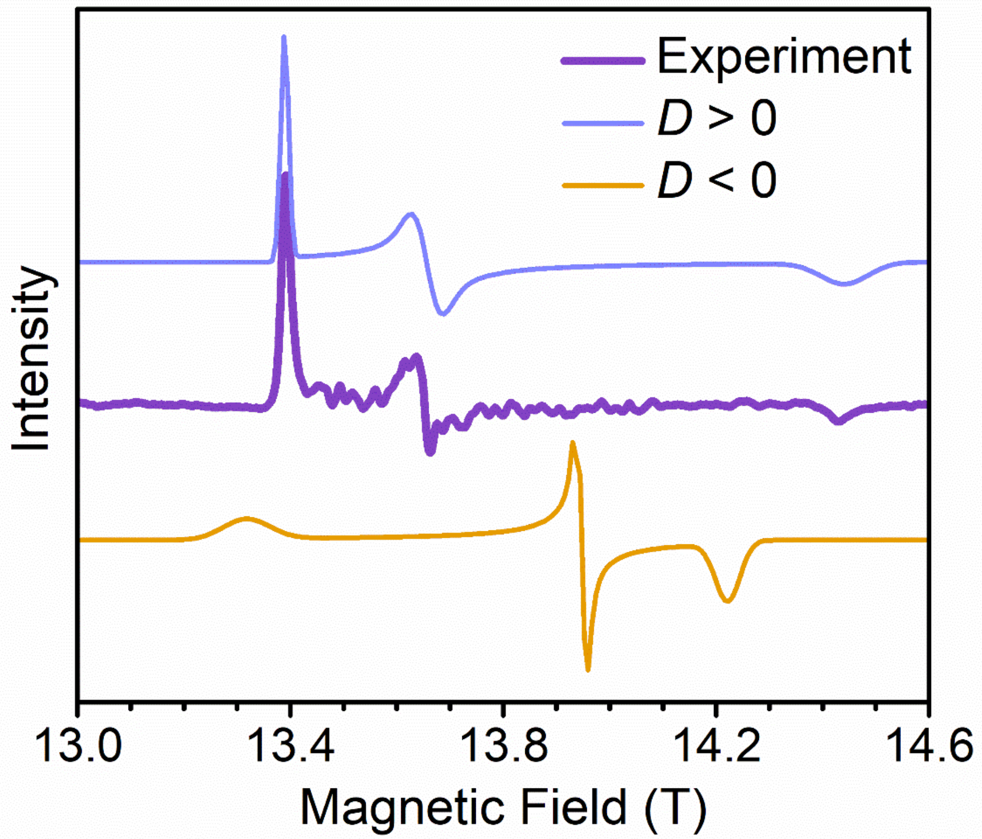

Figure S15 | Best spectral simulations of the 406.0 GHz cw-EPR spectrum of **1** with positive and negative *D* values at 3 K.

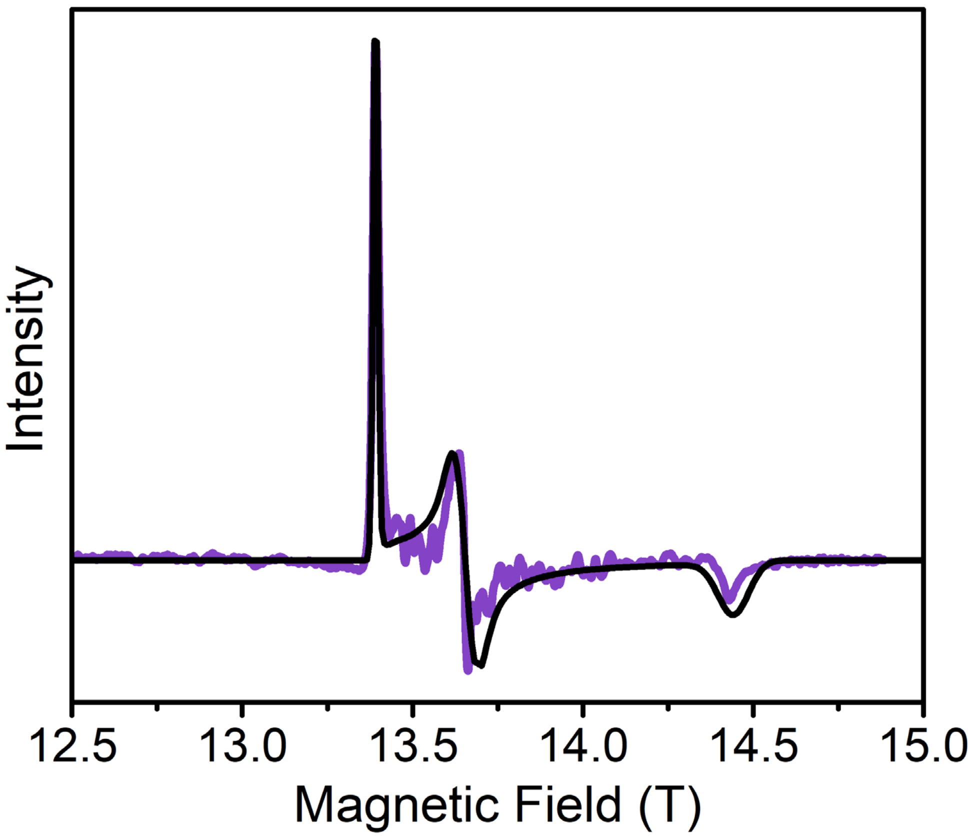

Figure S16 | Best spectral simulations of the 406.0 GHz cw-EPR spectrum of **1** at 3 K, with negative D value and the following spin Hamiltonian parameters: $g_x = 2.060$, $g_y = 2.155$, $g_z = 2.078$, $D = –0.55$ cm^{-1}, $|E| = 0.05$ cm^{-1}, H-strain = [3000 2000 0].

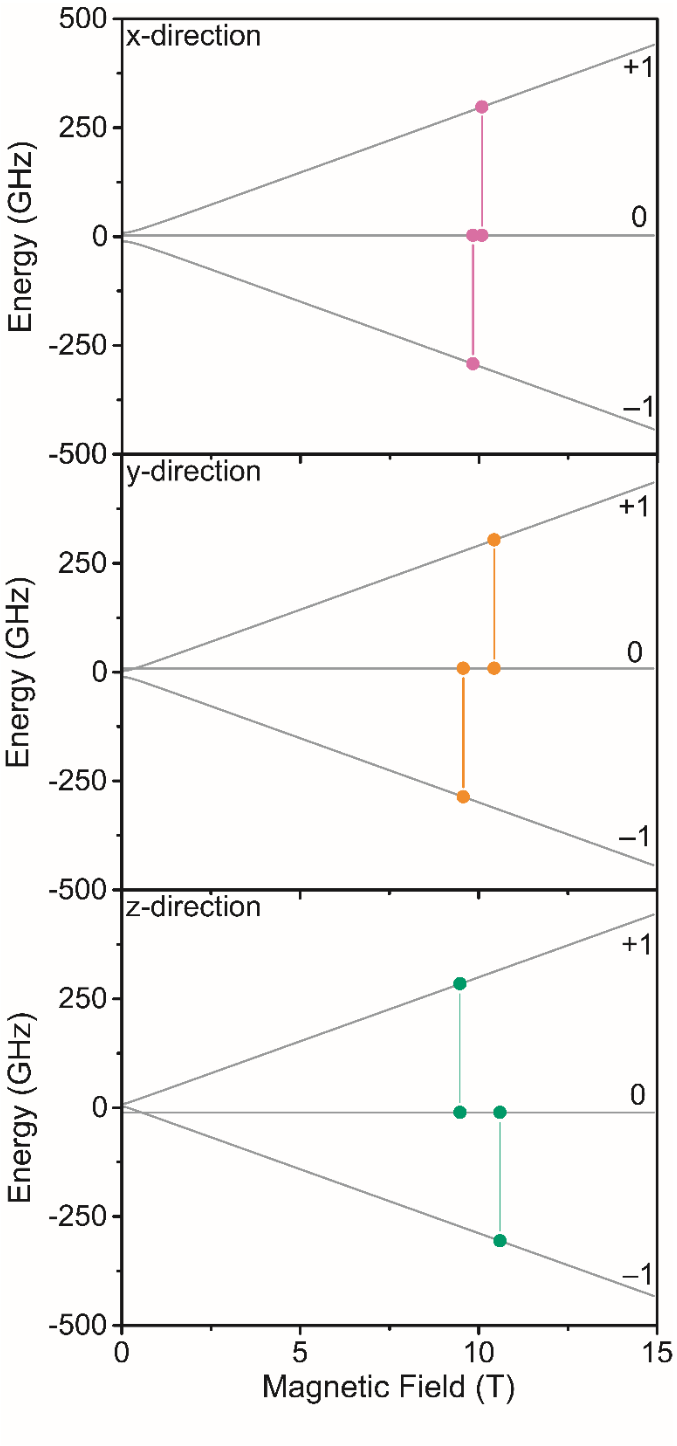

Figure S17 | Zeeman diagrams for 295.0 GHz EPR simulations of **1**. The Zeeman plots are simulated with the magnetic field oriented along the *x*-, *y*-, and *z*-molecular axes (top, middle, bottom, respectively) highlighting the transitions (vertical lines) observed in the spectrum. The −1, 0 and +1 M_S sublevels are labelled in the high field regime. The spin Hamiltonian parameters *g*, *D*, and *E* used for these simulations are given in the manuscript and SI.

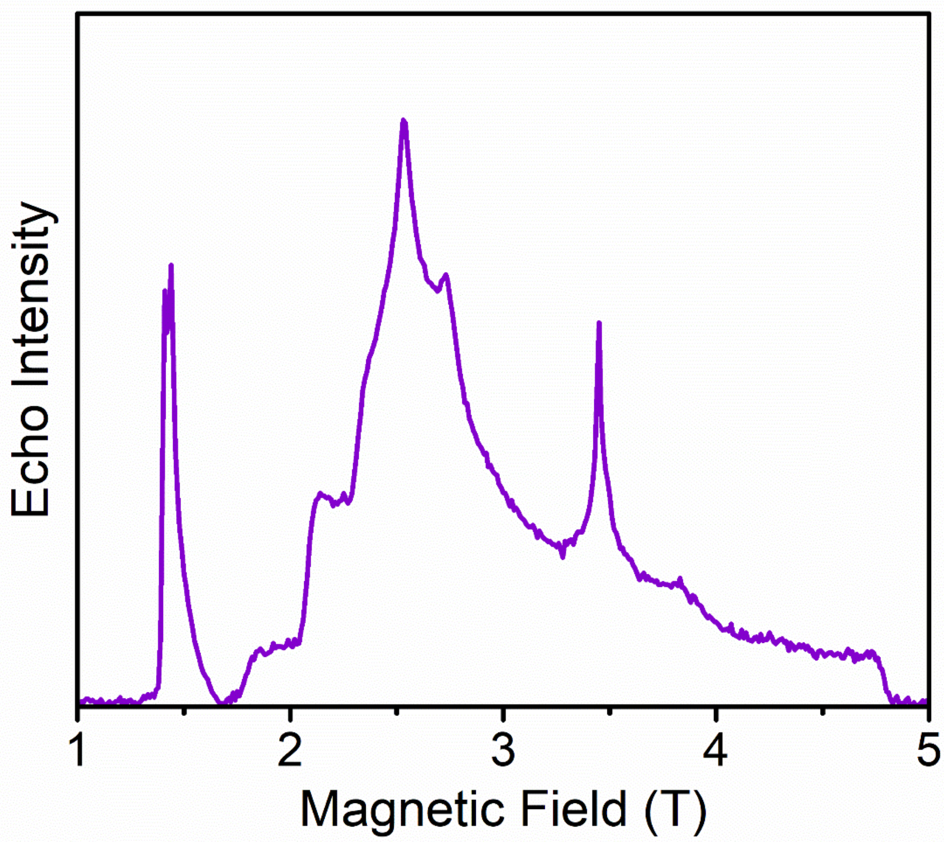

Figure S18 | Echo-detected field-swept spectrum of **1'** at 5 K, collected at 94 GHz.

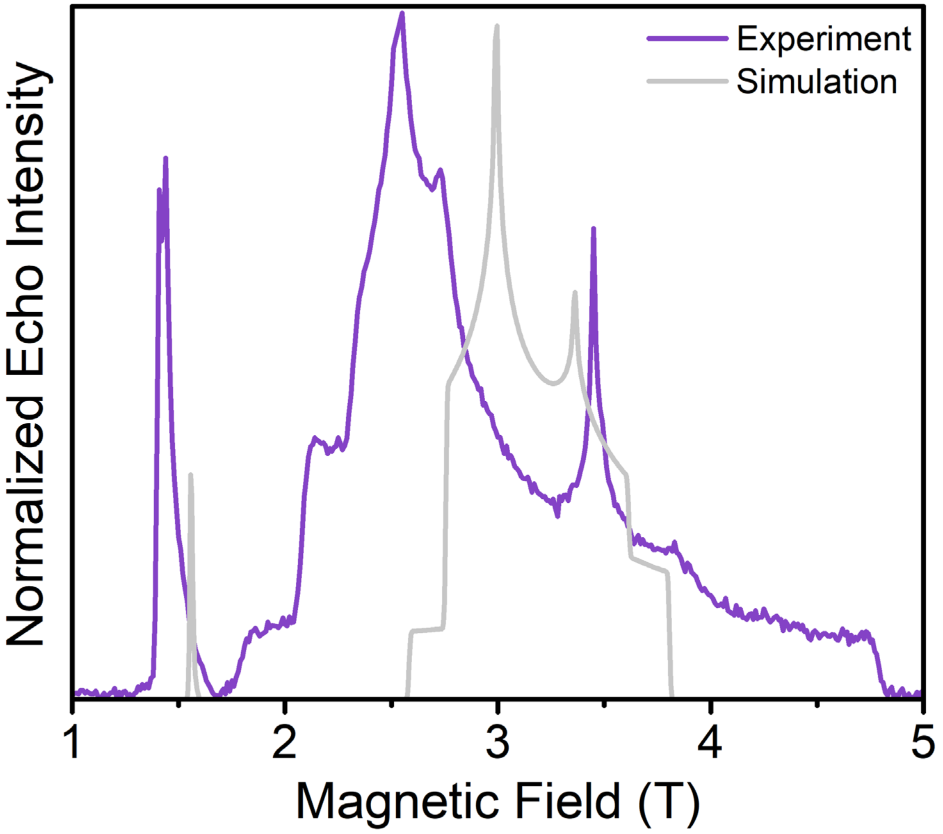

Figure S19 | Experimental (dark purple) and calculated (silver) absorptive cw mode of **1'** at 94 GHz, simulated using the cw-EPR parameters from **Table S1** (silver).

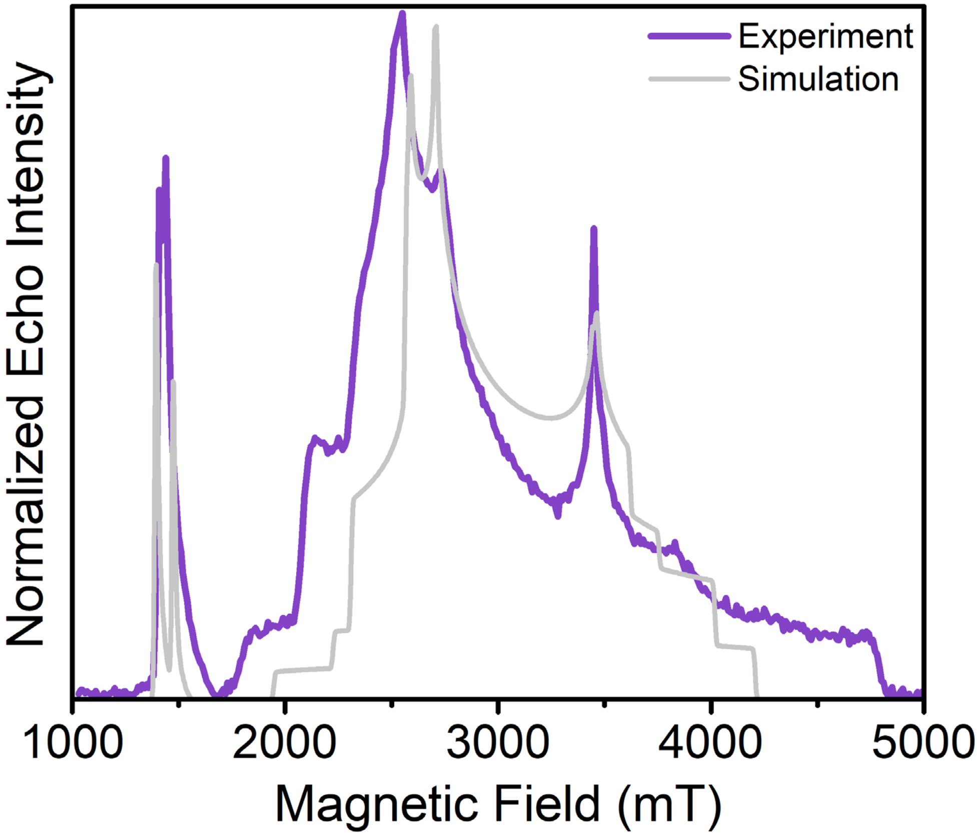

Figure S20 | Experimental and calculated absorptive cw mode of **1'** at 94 GHz, simulated as two distinct species using the following cw-EPR parameters: (1) g_{iso} = 2.18, D = +1.15 cm^{-1}, $|E|$ = 0.10 cm^{-1} (2) g_{iso} =2.15, D = +0.9 cm^{-1}, $|E|$ = 0.05 cm^{-1} (silver).

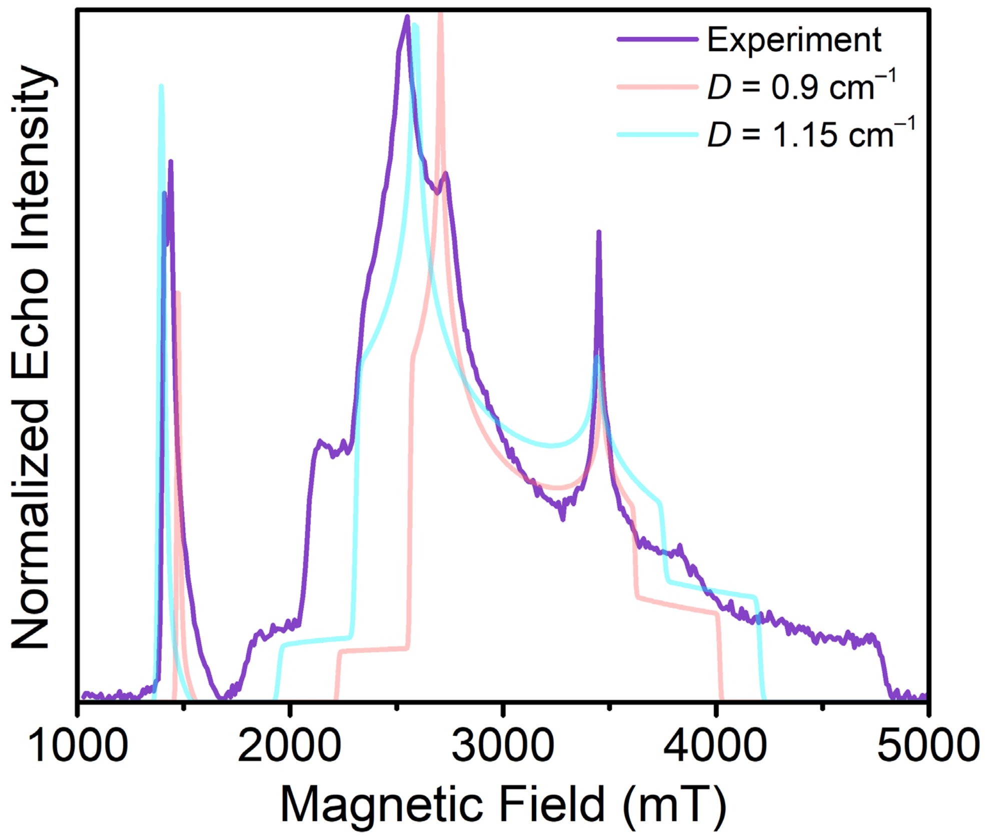

Figure S21 | Experimental and calculated absorptive cw mode of **1'** at 94 GHz, simulated as two distinct species using the following cw-EPR parameters: (1, light blue) g_{iso} = 2.18, D = +1.15 cm^{-1}, $|E|$ = 0.10 cm^{-1} (2, light pink) g_{iso} = 2.15, D = +0.9 cm^{-1}, $|E|$ = 0.05 cm^{-1}.

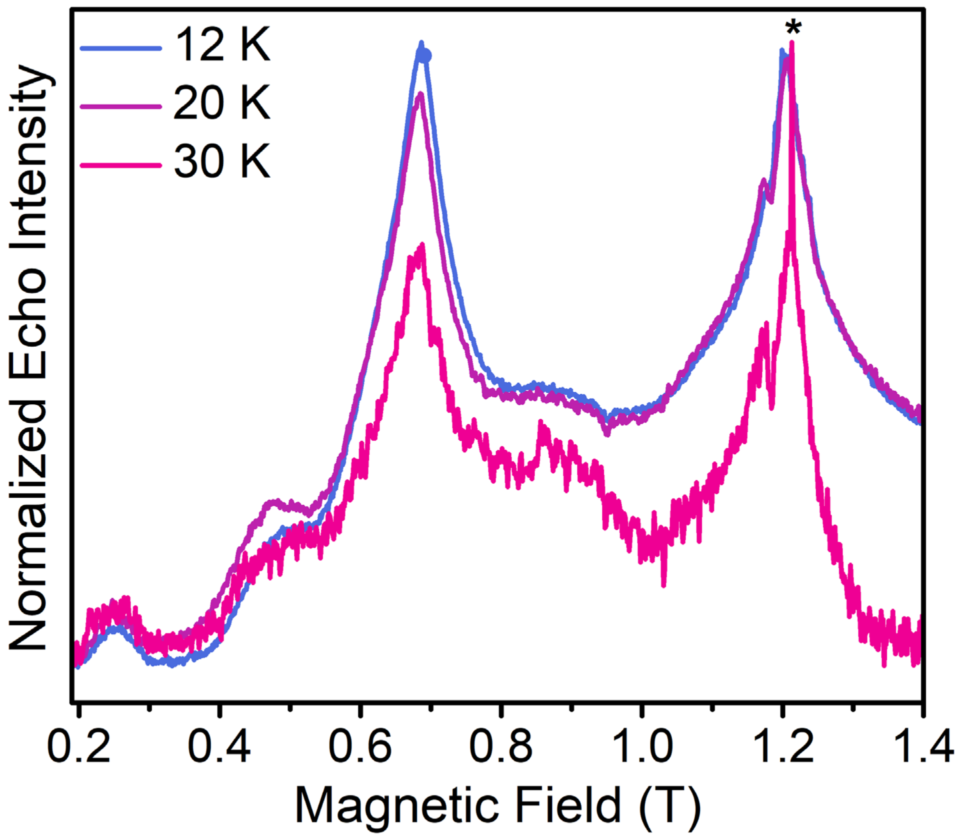

Figure S22 | Echo-detected field-swept spectrum of **1'** at 12 K, 20 K, and 30 K, collected at 34 GHz. * denotes a $g = 2$ impurity, which only becomes visible at 30 K when the T_m of **1'** becomes very short in comparison to the T_m of the $g = 2$ impurity.

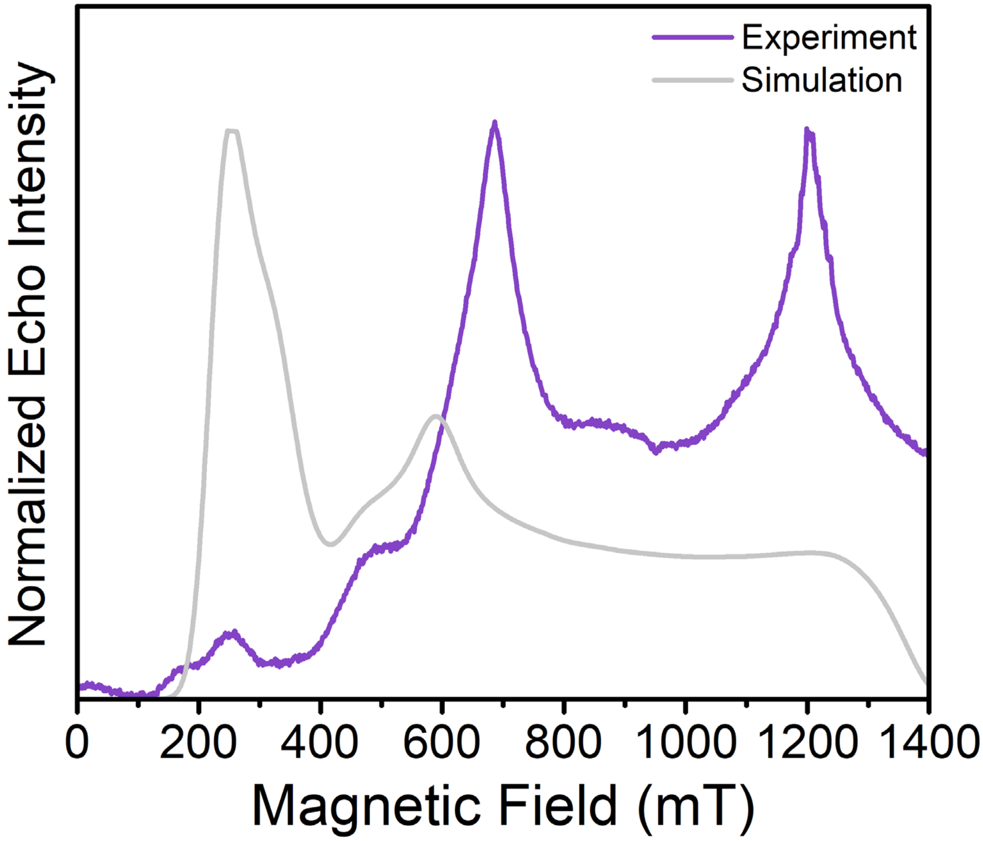

Figure S23 | Experimental and calculated absorptive cw mode of **1'** at 34 GHz, simulated as two distinct species using the following cw-EPR parameters: (1) g_{iso} = 2.18, D = +1.15 cm^{-1}, |E| = 0.10 cm^{-1} (2) g_{iso} = 2.15, D = +0.9 cm^{-1}, |E| = 0.05 cm^{-1} (silver).

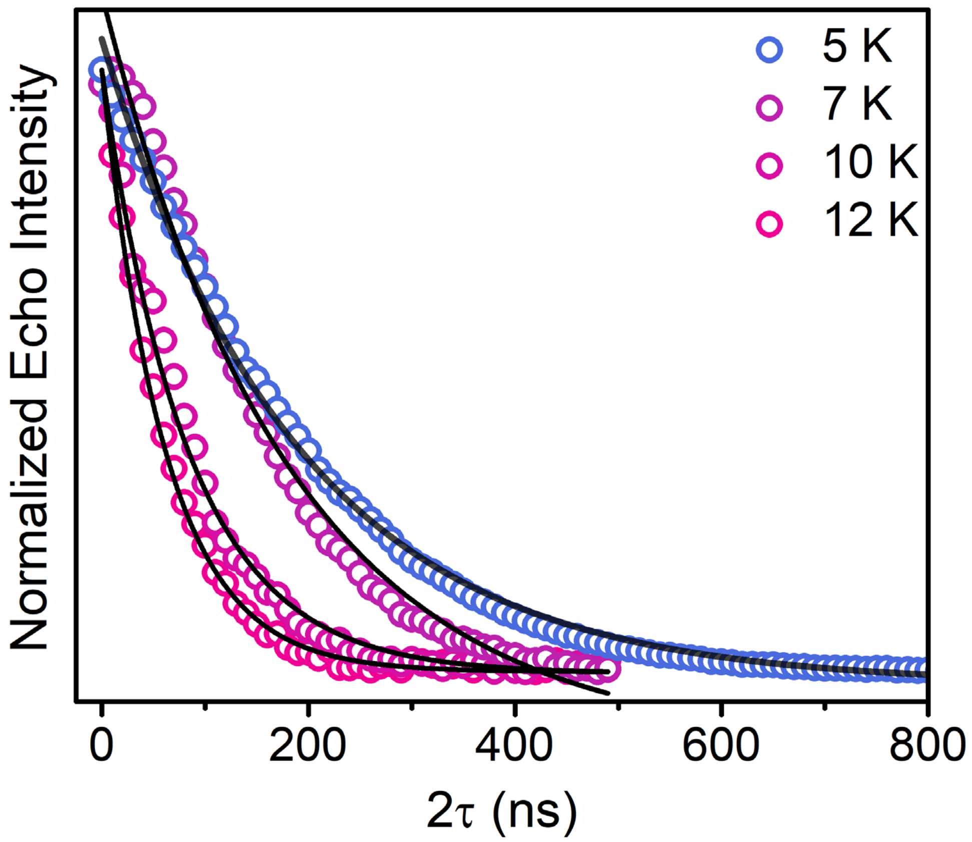

Figure S24 | Hahn-echo decay curves for **1'** at 1.33 T from 5 K to 12 K at 94 GHz. The echo decay intensities (circles) were fit (black lines) using a monoexponential function to yield the T_m times given in **Table S2**.

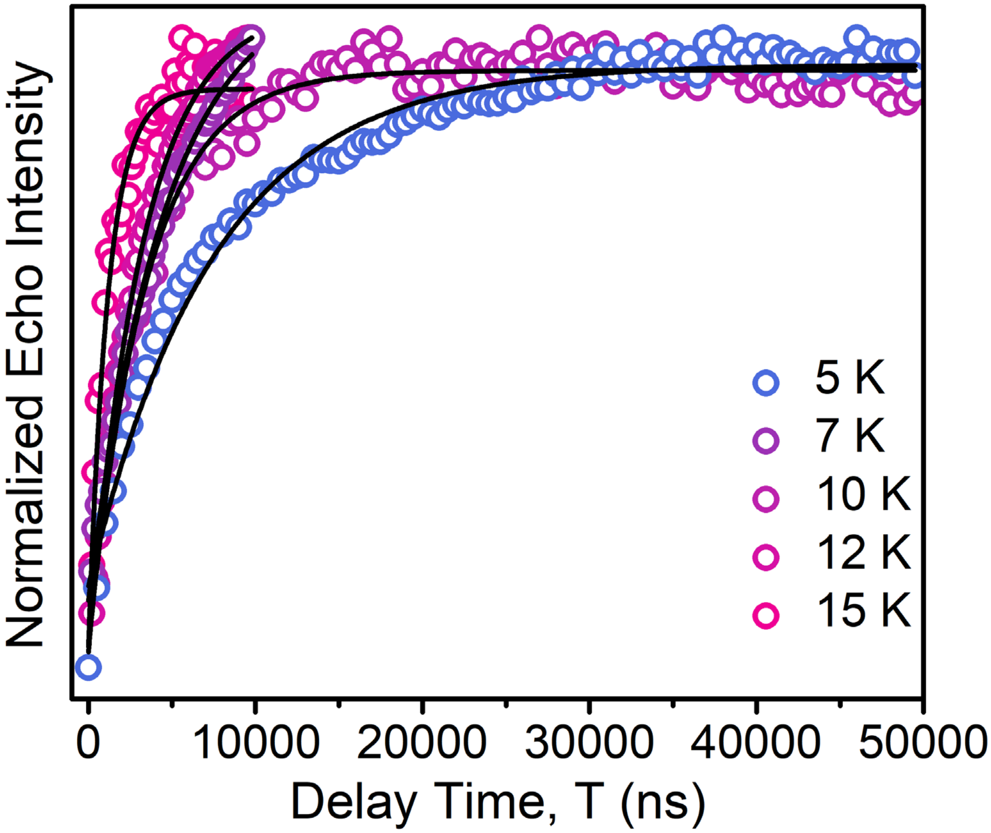

Figure S25 | Saturation-recovery data for **1'** at 1.33 T from 5 K to 12 K at 94 GHz. The saturation recovery data (circles) were fit (black lines) using a monoexponential recovery function to yield the T_1 times given in **Table S2**.

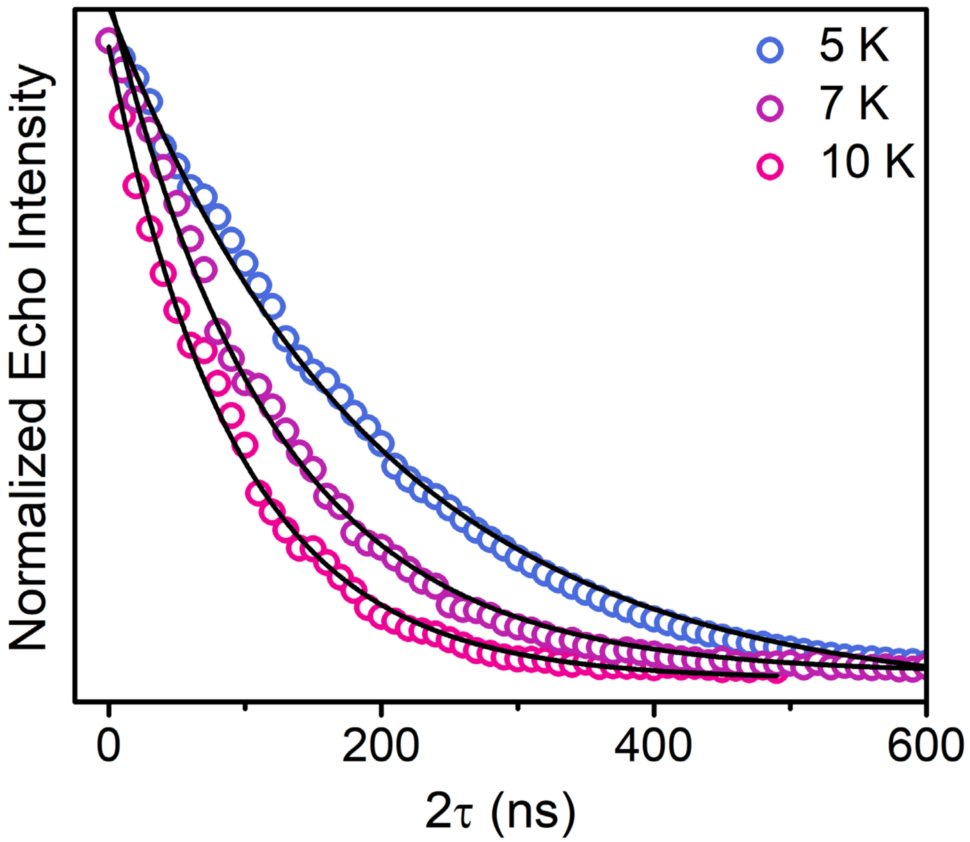

Figure S26 | Hahn-echo decay curves for **1'** at 2.50 T from 5 K to 10 K at 94 GHz. The echo decay intensities (circles) were fit (black lines) using a monoexponential function to yield the T_m times given in **Table S2**.

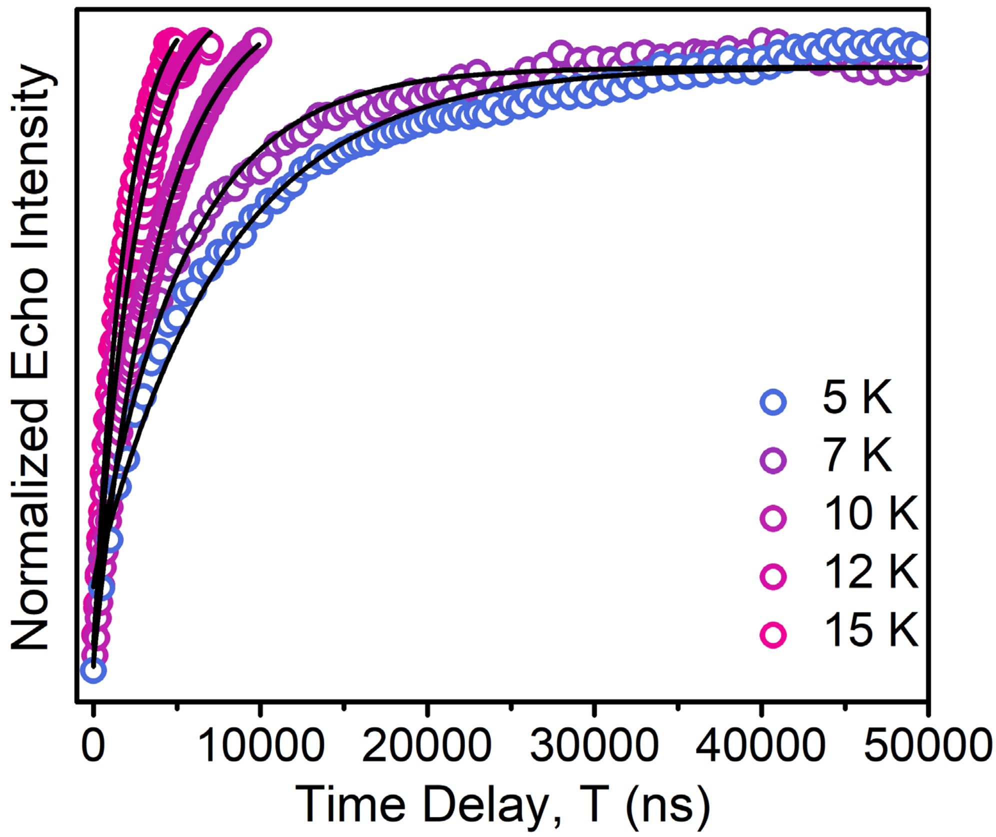

Figure S27 | Saturation-recovery data for **1'** at 2.50 T at 5 K and 7 K at 94 GHz. The saturation recovery data (circles) were fit (black lines) using a monoexponential recovery function to yield the T_1 times given in **Table S2**.

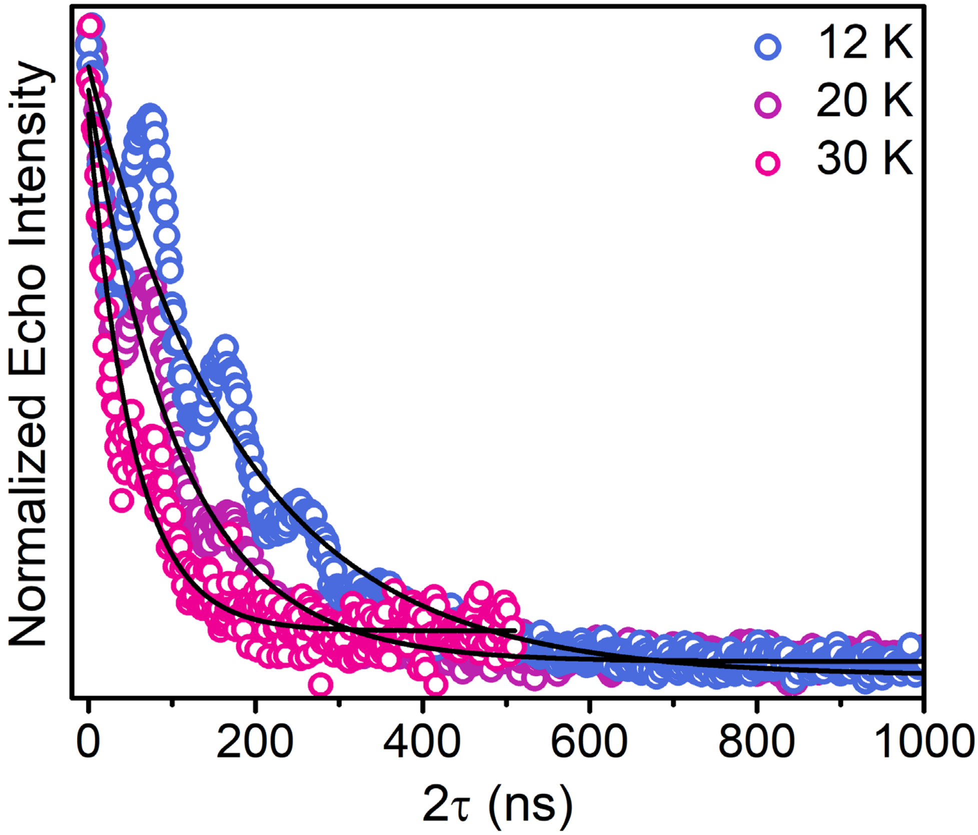

Figure S28 | Hahn-echo decay curves for **1'** at 0.25 T from 12 K to 30 K at 34 GHz. The echo decay intensities (circles) were fit (black lines) using a monoexponential function to yield the T_m times given in **Table S2**. Modulations in the decay curves are attributed to a combination of ^1H ESEEM and instrumental vibrations.

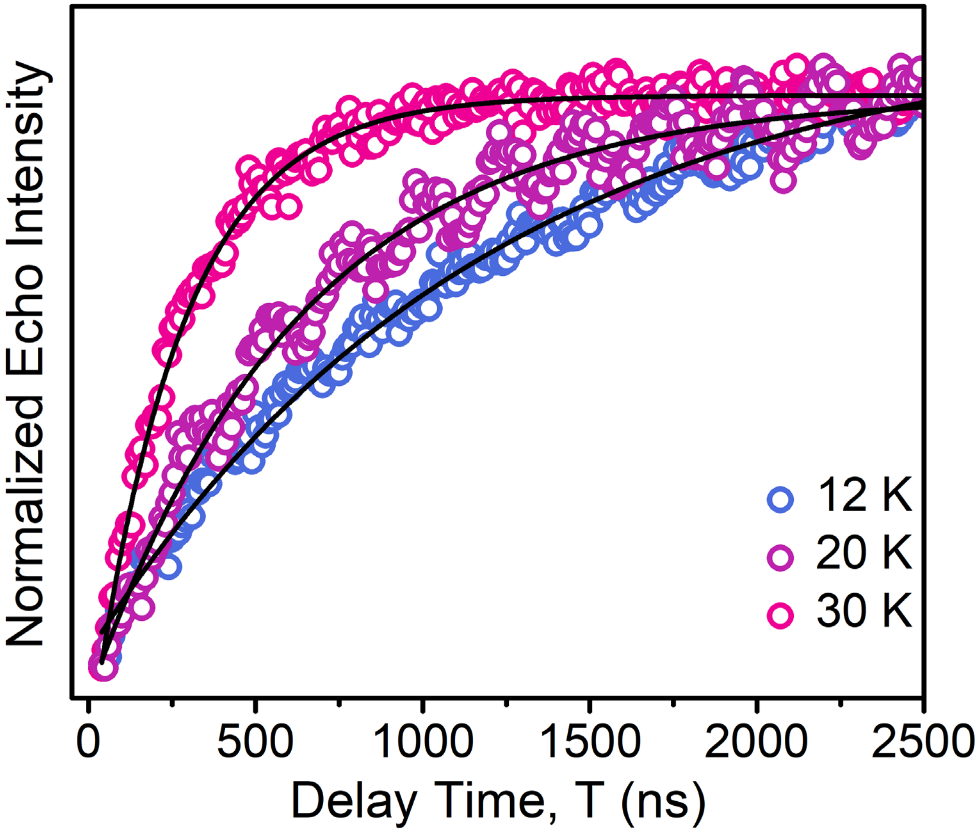

Figure S29 | Inversion-recovery data for **1'** at 0.25 T from 12 K to 30 K at 34 GHz. The inversion recovery data (circles) were fit (black lines) using a monoexponential recovery function to yield the T_1 times given in **Table S2**. Modulations in the decay curves are attributed to a combination of ^{1}H ESEEM and instrumental vibrations.

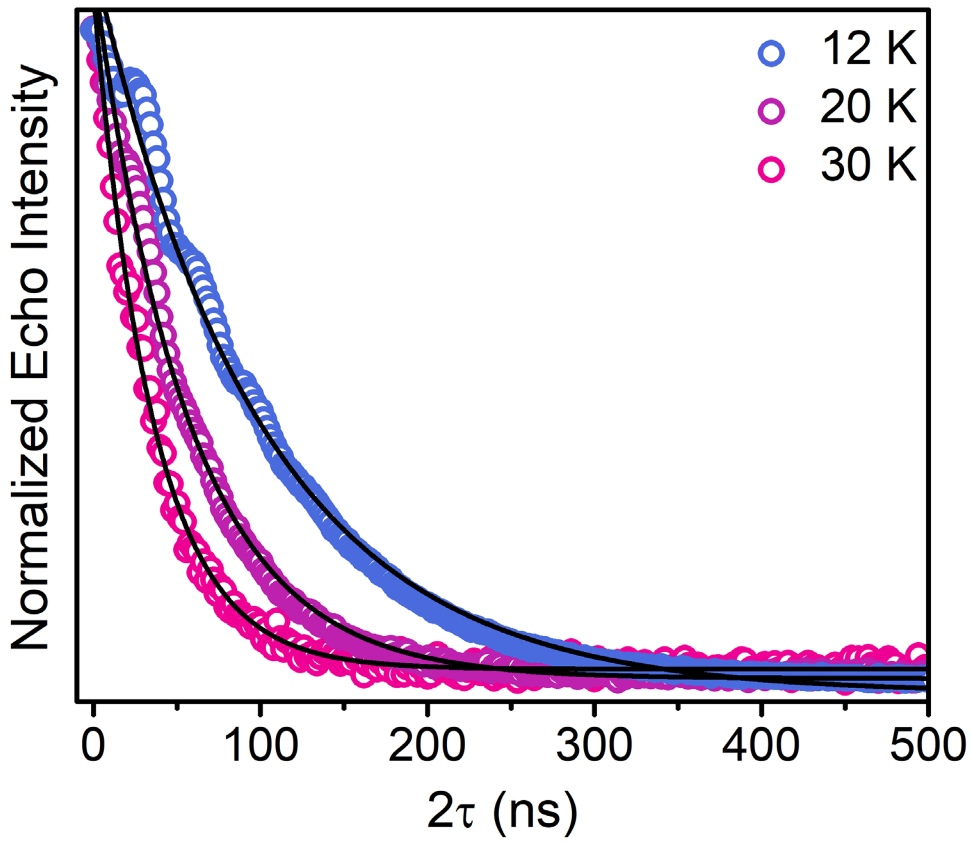

Figure S30 | Hahn-echo decay curves for **1'** at 0.69 T from 12 K to 30 K at 34 GHz. The echo decay intensities (circles) were fit (black lines) using a monoexponential function to yield the T_m times given in **Table S2**. Modulations in the decay curves are attributed to a combination of ^1H ESEEM and instrumental vibrations.

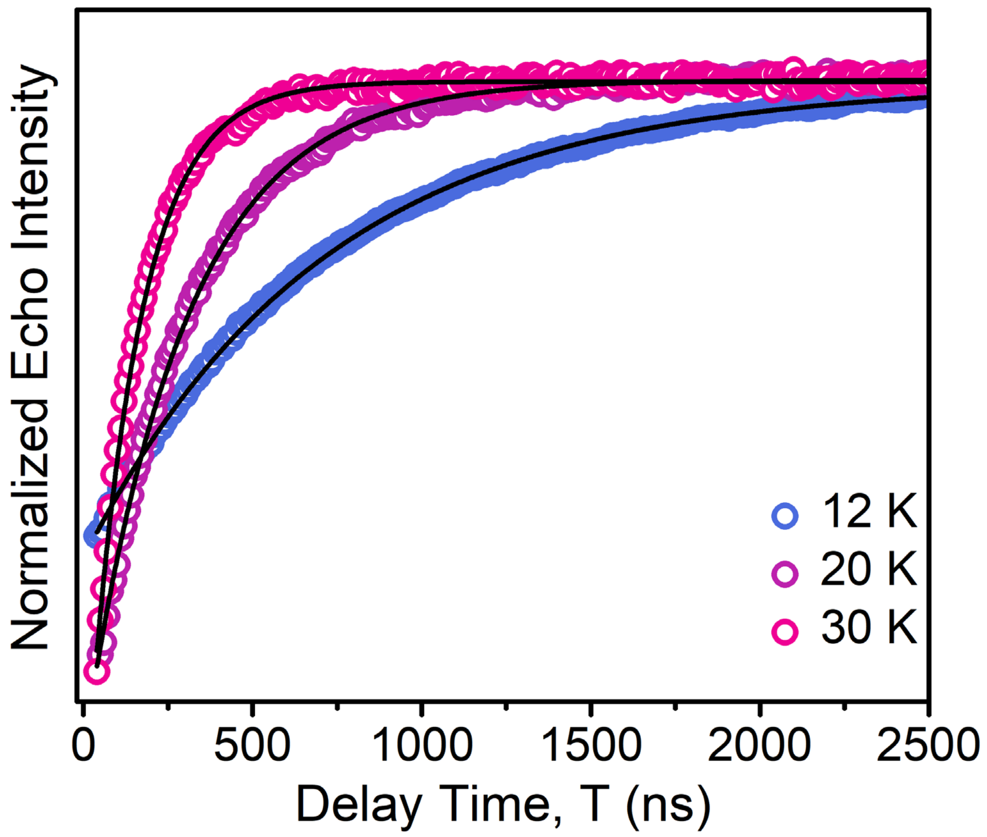

Figure S31 | Inversion-recovery data for **1'** at 0.69 T from 12 K to 30 K at 34 GHz. The inversion recovery data (circles) were fit (black lines) using a monoexponential recovery function to yield the T_1 times given in **Table S2**.

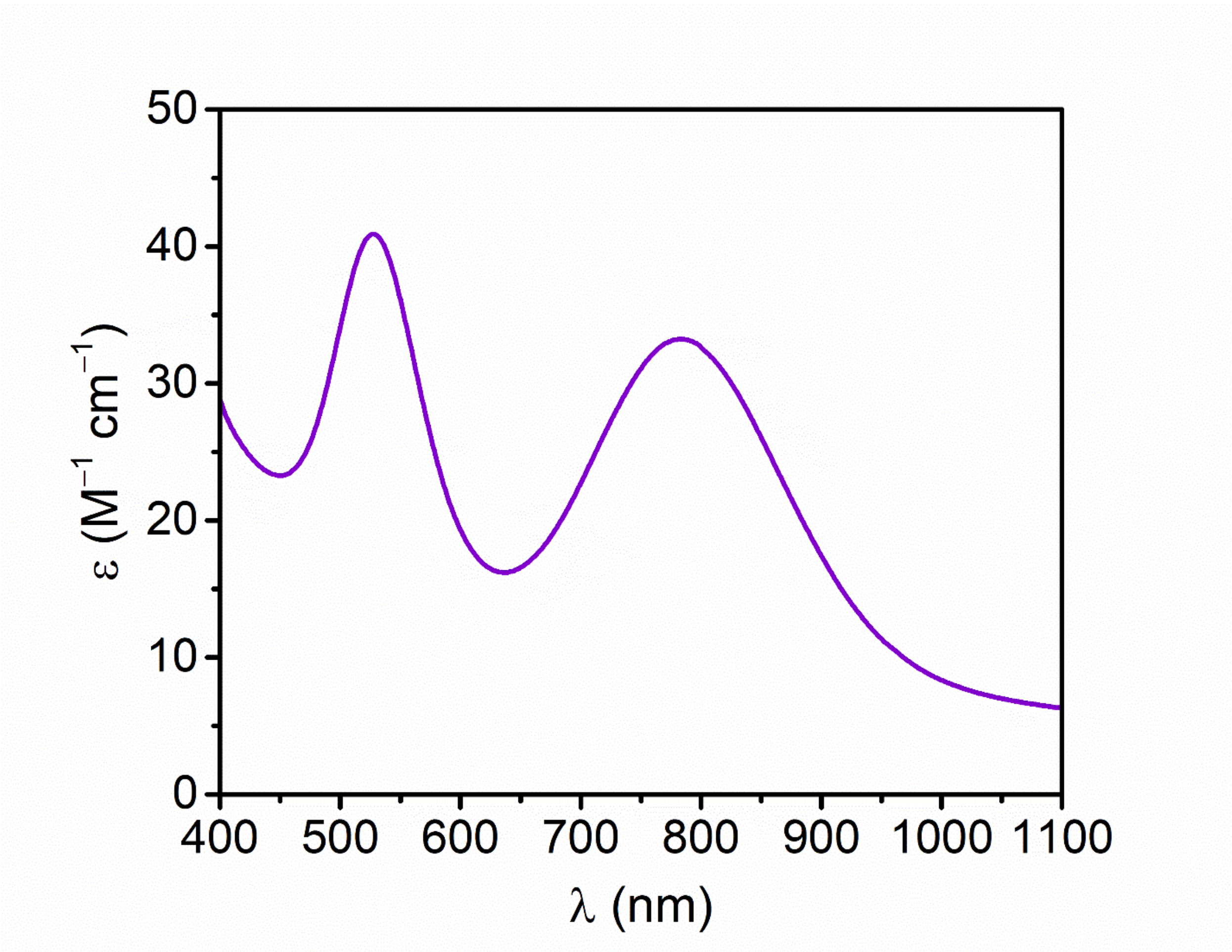

Figure S32 | Electronic absorption (UV-vis-NIR) spectrum collected in MeCN solution at room temperature for **1**.

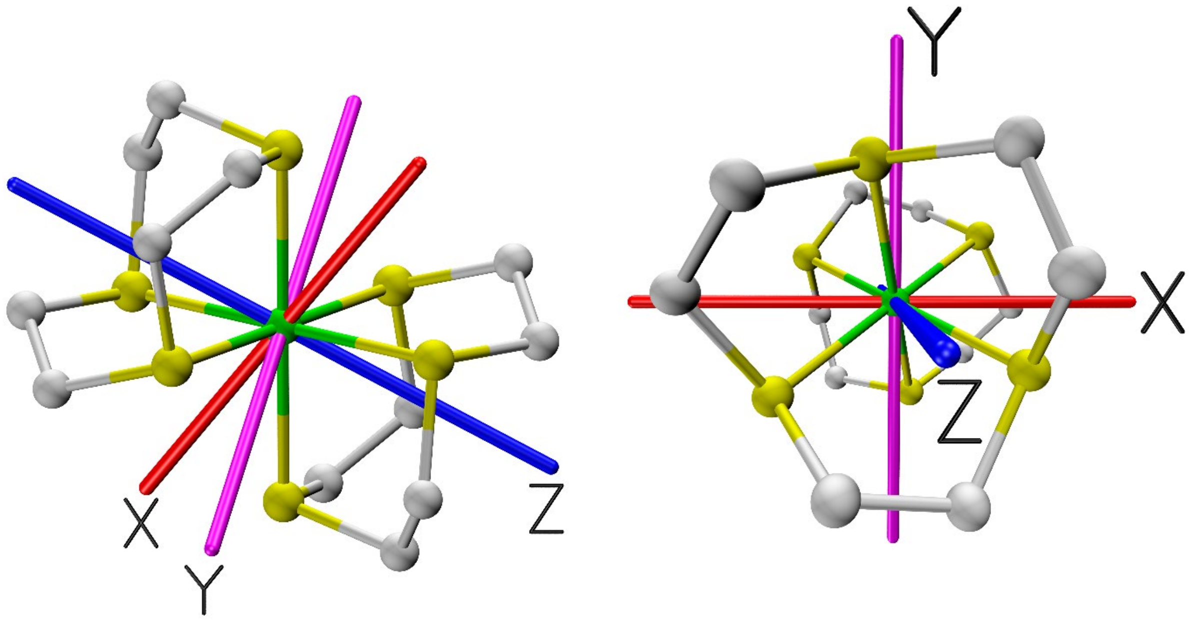

Figure S33 | Schematic view of the *D* tensor axes of **1** with *z*-axis along the pseudo three-fold axis of the complex.

References

(1) Setzer, W. N.; Ogle, C. A.; Wilson, G. S.; Glass, R. S. 1,4,7-Trithiacyclononane, a Novel Tridentate Thioether Ligand, and the Structure of Its Nickel(II), Cobalt(II), and Copper(II) Complexes. *Inorg. Chem.* **1983**, *22* (2), 266–271.

(2) Setzer, W. N.; Guo, Q.; Grant, G. J.; Hubbard, J. L.; Glass, R. S.; VanDerveer, D. G. 1,4,7-Trithiacyclononane as a Tridentate Ligand for Complexation of Heavy-Metal Ions: Synthesis and Complexation Studies of Mesocyclic and Macrocyclic Polythioethers IV. *Heteroat. Chem.* **1990**, *1* (4), 317–325.

(3) Bain, G. A.; Berry, J. F. Diamagnetic Corrections and Pascal's Constants. *J. Chem. Educ.* **2008**, *85* (4), 532.

(4) Hassan, A. K.; Pardi, L. A.; Krzystek, J.; Sienkiewicz, A.; Goy, P.; Rohrer, M.; Brunel, L.-C. Ultrawide Band Multifrequency High-Field EMR Technique: A Methodology for Increasing Spectroscopic Information. *J. Magn. Reson.* **2000**, *142* (2), 300–312.

(5) Stoll, S.; Schweiger, A. EasySpin, a Comprehensive Software Package for Spectral Simulation and Analysis in EPR. *J. Magn. Reson.* **2006**, *178* (1), 42–55.

(6) Mombourquette, M. J.; Weil, J. A. Simulation of Magnetic Resonance Powder Spectra. *J. Magn. Reson.* **1992**, *99* (1), 37–44.

(7) Krzystek, J.; Park, J.-H.; Meisel, M. W.; Hitchman, M. A.; Stratemeier, H.; Brunel, L.-C.; Telser, J. EPR Spectra from "EPR-Silent" Species: High-Frequency and High-Field EPR Spectroscopy of Pseudotetrahedral Complexes of Nickel(II). *Inorg. Chem.* **2002**, *41* (17), 4478–4487.

(8) Janas, Z.; Jezierska, J.; Ozarowski, A.; Bieńko, A.; Lis, T.; Jezierski, A.; Krawczyk, M. Investigation of Vanadium(III) and Vanadium(IV) Compounds Supported by the Linear Diaminebis(Phenolate) Ligands: Correlation between Structures and Magnetic Properties. *Dalton Trans.* **2021**, *50*, 5184–5196.

(9) Collison, D. Single and Double Quantum Transitions in the Multi-Frequency Continuous Wave Electron Paramagnetic Resonance (CwEPR) of Three Six Co-Ordinate Nickel(II) Complexes: [Ni(EtL)$_2$(Me$_5$Dien)] and [Ni(5-Methylpyrazole)$_6$]X$_2$, X = $(ClO_4)^-$ or $(BF_4)^-$. The Single Crystal X-Ray Structure at Room Temperature of [Ni(5-Methylpyrazole)$_6$](ClO$_4$)$_2$. *J. Chem. Soc., Faraday Trans.* **1998**, *94*, 3019–3025.

(10) Fataftah, M. S.; Zadrozny, J. M.; Rogers, D. M.; Freedman, D. E. A Mononuclear Transition Metal Single-Molecule Magnet in a Nuclear Spin-Free Ligand Environment. *Inorg. Chem.* **2014**, *53* (19), 10716–10721.

(11) Fataftah, M. S.; Zadrozny, J. M.; Coste, S. C.; Graham, M. J.; Rogers, D. M.; Freedman, D. E. Employing Forbidden Transitions as Qubits in a Nuclear Spin-Free Chromium Complex. *J. Am. Chem. Soc.* **2016**, *138* (4), 1344–1348.

(12) Cruickshank, P. A. S.; Bolton, D. R.; Robertson, D. A.; Hunter, R. I.; Wylde, R. J.; Smith, G. M. A Kilowatt Pulsed 94 GHz Electron Paramagnetic Resonance Spectrometer with High Concentration Sensitivity, High Instantaneous Bandwidth, and Low Dead Time. *Rev. Sci. Instrum.* **2009**, *80* (10), 103102.

(13) Lee, C.; Yang, W.; Parr, R. G. Development of the Colle-Salvetti Correlation-Energy Formula into a Functional of the Electron Density. *Phys. Rev. B* **1988**, *37* (2), 785–789.

(14) Becke, A. D. Density-functional Thermochemistry. III. The Role of Exact Exchange. *J. Chem. Phys.* **1993**, *98* (7), 5648–5652.

(15) Stephens, P. J.; Devlin, F. J.; Chabalowski, C. F.; Frisch, M. J. Ab Initio Calculation of Vibrational Absorption and Circular Dichroism Spectra Using Density Functional Force Fields. *J. Phys. Chem.* **1994**, *98* (45), 11623–11627.
(16) Weigend, F.; Ahlrichs, R. Balanced Basis Sets of Split Valence, Triple Zeta Valence and Quadruple Zeta Valence Quality for H to Rn: Design and Assessment of Accuracy. *Phys. Chem. Chem. Phys.* **2005**, *7* (18), 3297–3305.
(17) Neese, F. An Improvement of the Resolution of the Identity Approximation for the Formation of the Coulomb Matrix. *J. Comput. Chem.* **2003**, *24* (14), 1740–1747.
(18) Neese, F.; Wennmohs, F.; Hansen, A.; Becker, U. Efficient, Approximate and Parallel Hartree–Fock and Hybrid DFT Calculations. A 'Chain-of-Spheres' Algorithm for the Hartree–Fock Exchange. *Chem. Phys.* **2009**, *356* (1), 98–109.
(19) Weigend, F. Accurate Coulomb-Fitting Basis Sets for H to Rn. *Phys. Chem. Chem. Phys.* **2006**, *8* (9), 1057–1065.
(20) Grimme, S.; Ehrlich, S.; Goerigk, L. Effect of the Damping Function in Dispersion Corrected Density Functional Theory. *J. Comput. Chem.* **2011**, *32* (7), 1456–1465.
(21) Grimme, S.; Antony, J.; Ehrlich, S.; Krieg, H. A Consistent and Accurate Ab Initio Parametrization of Density Functional Dispersion Correction (DFT-D) for the 94 Elements H-Pu. *J. Chem. Phys.* **2010**, *132* (15), 154104.
(22) Perdew, J. P.; Burke, K.; Ernzerhof, M. Generalized Gradient Approximation Made Simple. *Phys. Rev. Lett.* **1996**, *77* (18), 3865–3868.
(23) González, E.; Rodrigue-Witchel, A.; Reber, C. Absorption Spectroscopy of Octahedral Nickel(II) Complexes: A Case Studyof Interactions between Multiple Electronic Excited States. *Coord. Chem. Rev.* **2007**, *251*, 351–363.
(24) Stranger, R.; McMahon, K. L.; Gahan, L. R.; Bruce, J. I.; Hambley, T. W. Spin−Orbit Mixing and Nephelauxetic Effects in the Electronic Spectra of Nickel(II)-Encapsulating Complexes Involving Nitrogen and Sulfur Donors. *Inorg. Chem.* **1997**, *36* (16), 3466–3475.
(25) Brik, M. G.; Camardello, S. J.; Srivastava, A. M.; Avram, N. M.; Suchocki, A. Spin-Forbidden Transitions in the Spectra of Transition Metal Ions and Nephelauxetic Effect. *ECS J. Solid State Sci. Technol.* **2016**, *5* (1), R3067–R3077.
(26) Caspar, J. V.; Meyer, T. J. Application of the Energy Gap Law to Nonradiative, Excited-State Decay. *J. Phys. Chem.* **1983**, *87* (6), 952–957.
(27) Cooper, S. R.; Rawle, S. C.; Hartman, J. R.; Hintsa, E. J.; Admans, G. A. Crown Thioether Chemistry: Homoleptic Hexakis(Thioether) Complexes of Nickel(II). *Inorg. Chem.* **1988**, *27* (7), 1209–1214.
(28) Boča, R. Zero-Field Splitting in Metal Complexes. *Coord. Chem. Rev.* **2004**, *248* (9), 757–815.
(29) Nishijo, J.; Miyazaki, A.; Enoki, T. Structure and Physical Properties of Molecule-Based Magnets Including Transition Metal Complexes of Crown Thioethers. *Bull. Chem. Soc. Jpn.* **2004**, *77* (4), 715–727.
(30) Nishijo, J.; Miyazaki, A.; Enoki, T. Weak-Ferromagnetism in Molecular Magnets Based on Transition Metal Complexes of Crown Thioether. *Polyhedron* **2003**, *22* (14), 1755–1758.
(31) Manson, J. L.; Lapidus, S. H.; Stephens, P. W.; Peterson, P. K.; Carreiro, K. E.; Southerland, H. I.; Lancaster, T.; Blundell, S. J.; Steele, A. J.; Goddard, P. A.; Pratt, F. L.; Singleton, J.; Kohama, Y.; McDonald, R. D.; Sesto, R. E. D.; Smith, N. A.; Bendix, J.; Zvyagin, S. A.; Kang, J.; Lee, C.; Whangbo, M.-H.; Zapf, V. S.; Plonczak, A. Structural, Electronic, and Magnetic Properties of Quasi-1D Quantum Magnets $[Ni(HF_2)(Pyz)_2]X$ (Pyz = Pyrazine; X

= PF_6^-, SbF_6^-) Exhibiting Ni-FHF-Ni and Ni-Pyz-Ni Spin Interactions. *Inorg. Chem.* **2011**, *50* (13), 5990–6009.
(32) Abragam, A.; Bleaney, B. *Electron Paramagnetic Resonance of Transition Ions*, Illustrated edition.; Oxford University Press: Oxford, 2012.
(33) Singh, S. K.; Gupta, T.; Badkur, P.; Rajaraman, G. Magnetic Anisotropy of Mononuclear Ni^{II} Complexes: On the Importance of Structural Diversity and the Structural Distortions. *Chem. Eur. J.* **2014**, *20* (33), 10305–10313.
(34) Titiš, J.; Boča, R. Magnetostructural *D* Correlation in Nickel(II) Complexes: Reinvestigation of the Zero-Field Splitting. *Inorg. Chem.* **2010**, *49* (9), 3971–3973.
(35) Jackson, C. E.; Lin, C.-Y.; van Tol, J.; Zadrozny, J. M. Orientation Dependence of Phase Memory Relaxation in the V(IV) Ion at High Frequencies. *Chem. Phys. Lett.* **2020**, *739*, 137034.
(36) Wojnar, M. K.; Laorenza, D. W.; Schaller, R. D.; Freedman, D. E. Nickel(II) Metal Complexes as Optically Addressable Qubit Candidates. *J. Am. Chem. Soc.* **2020**, *142* (35), 14826–14830.
(37) Heß, B. A.; Marian, C. M.; Wahlgren, U.; Gropen, O. A Mean-Field Spin-Orbit Method Applicable to Correlated Wavefunctions. *Chem. Phys. Lett.* **1996**, *251* (5), 365–371.
(38) Neese, F. Efficient and Accurate Approximations to the Molecular Spin-Orbit Coupling Operator and Their Use in Molecular g-Tensor Calculations. *J. Chem. Phys.* **2005**, *122* (3), 034107.
(39) Neese, F. Prediction of Electron Paramagnetic Resonance *g* Values Using Coupled Perturbed Hartree–Fock and Kohn–Sham Theory. *J. Chem. Phys.* **2001**, *115* (24), 11080–11096.
(40) Suaud, N.; Rogez, G.; Rebilly, J.-N.; Bouammali, M.-A.; Guihéry, N.; Barra, A.-L.; Mallah, T. Playing with Magnetic Anisotropy in Hexacoordinated Mononuclear Ni(II) Complexes, An Interplay Between Symmetry and Geometry. *Appl. Magn. Reson.* **2020**, *51* (11), 1215–1231.
(41) Maganas, D.; Krzystek, J.; Ferentinos, E.; Whyte, A. M.; Robertson, N.; Psycharis, V.; Terzis, A.; Neese, F.; Kyritsis, P. Investigating Magnetostructural Correlations in the Pseudooctahedral *Trans*-[Ni^{II}{($OPPh_2$)($EPPh_2$)N}$_2$($Sol)_2$] Complexes (E = S, Se; Sol = DMF, THF) by Magnetometry, HFEPR, and Ab Initio Quantum Chemistry. *Inorg. Chem.* **2012**, *51* (13), 7218–7231.
(42) Cahier, B.; Perfetti, M.; Zakhia, G.; Naoufal, D.; El-Khatib, F.; Guillot, R.; Rivière, E.; Sessoli, R.; Barra, A.-L.; Guihéry, N.; Mallah, T. Magnetic Anisotropy in Pentacoordinate Ni^{II} and Co^{II} Complexes: Unraveling Electronic and Geometrical Contributions. *Chem. – Eur. J.* **2017**, *23* (15), 3648–3657.
(43) Singh, S. K.; Rajaraman, G. Deciphering the Origin of Giant Magnetic Anisotropy and Fast Quantum Tunnelling in Rhenium(IV) Single-Molecule Magnets. *Nat. Commun.* **2016**, *7* (1), 10669.
(44) Ruamps, R.; Batchelor, L. J.; Maurice, R.; Gogoi, N.; Jiménez-Lozano, P.; Guihéry, N.; de Graaf, C.; Barra, A.-L.; Sutter, J.-P.; Mallah, T. Origin of the Magnetic Anisotropy in Heptacoordinate Ni^{II} and Co^{II} Complexes. *Chem. – Eur. J.* **2013**, *19* (3), 950–956.
(45) Lee, J. P.; Grant, G. J.; Noll, B. C. Bis(1,4,7-Trithia-cyclo-nona-ne)Nickel(II) Bis-(Tetra-fluorido-borate) Nitro-methane Disolvate. *Acta Crystallogr. Sect. E Struct. Rep. Online* **2011**, *67* (10), m1417–m1418.
